# Effects of Coenzyme Q10 on Lipid, Glycemic, and Inflammatory Markers in Metabolic Disorders: A Systematic Review and Meta‐Analysis

**DOI:** 10.1155/jdr/5587445

**Published:** 2026-05-26

**Authors:** Zhuo Zhang, Zhuo Liu, Yuli Geng, Yanjing Huang, Runan Hu, Fan Li, Yufan Song, Mingmin Zhang

**Affiliations:** ^1^ Institute of Integrated Traditional Chinese and Western Medicine, Tongji Hospital, Tongji Medical College, Huazhong University of Science and Technology, Wuhan, China, hust.edu.cn; ^2^ Department of Integrated Traditional Chinese and Western Medicine, Tongji Hospital, Tongji Medical College, Huazhong University of Science and Technology, Wuhan, China, hust.edu.cn

**Keywords:** coenzyme Q10, glucose metabolism, inflammation, lipid, meta-analysis

## Abstract

**Background:**

The rising prevalence of metabolic disorders characterized by dyslipidemia and impaired glucose metabolism has increased interest in effective nutritional supplements. This systematic review and meta‐analysis was conducted to evaluate the effects of coenzyme Q10 (CoQ10) on lipid profile, glycemic control, and inflammatory markers.

**Methods:**

PubMed, Web of Science, Scopus, Embase, Cochrane Library, and ClinicalTrials.gov were searched for eligible randomized controlled trials (RCTs). Random‐effects models were applied to pool weighted mean differences (WMDs) or standardized mean differences (SMDs) with 95% confidence intervals (CIs).

**Results:**

A total of 64 RCTs (3422 participants) were included. Lipid profile: CoQ10 supplementation significantly improved blood triglyceride (TG, WMD = −5.67 mg/dL; 95% CI: −10.57, −0.77; *p* = 0.023), total cholesterol (TC, WMD = −4.86 mg/dL; 95% CI: −8.41, −1.30; *p* = 0.007), high‐density lipoprotein cholesterol (HDL‐C, WMD = 1.07 mg/dL; 95% CI: 0.22, 1.92; *p* = 0.013), and low‐density lipoprotein cholesterol (LDL‐C, WMD = −3.98 mg/dL; 95% CI: −7.00, −0.97; *p* = 0.010). Glycemic control: CoQ10 significantly reduced hemoglobin A1C (HbA1c, WMD = −0.22*%*; 95% CI: −0.37, −0.06; *p* = 0.006), fasting glucose (WMD = −10.07 mg/dL; 95% CI: −14.75, −5.39; *p* < 0.001), fasting insulin (FINS, WMD = −2.94 *μ*IU/mL; 95% CI: −4.63, −1.25; *p* = 0.001), and homeostasis model assessment–insulin resistance (HOMA‐IR, WMD = −0.82; 95% CI: −1.36, −0.28; *p* = 0.003). Inflammatory markers: CoQ10 significantly decreased C‐reactive protein (CRP, WMD = −0.44 mg/L; 95% CI: −0.79, −0.09; *p* = 0.013), tumor necrosis factor‐*α* (TNF‐*α*, SMD = −1.01; 95% CI: −1.56, −0.64; *p* = 0.013), and interleukin‐6 (IL‐6, SMD = −0.42; 95% CI: −0.79, −0.05; *p* = 0.027) levels.

**Conclusions:**

CoQ10 supplementation has beneficial effects on lipid metabolism, glycemic control, and inflammation among individuals with metabolic disorders.

## 1. Introduction

Abnormal lipid and glycemic metabolism is closely linked to the occurrence and development of various metabolic diseases, including dyslipidemia, diabetes, coronary artery disease (CAD), and metabolic syndrome, and poses a significant global health burden. Importantly, disturbances in lipid and glucose metabolism are interrelated and may exacerbate one another. For instance, hyperglycemia and insulin resistance (IR) enhance hepatic de novo lipogenesis and increase adipose fatty acid metabolism, contributing to dyslipidemia in diabetes [1, 2]. Conversely, ectopic lipid accumulation and elevated circulating free fatty acids can further impair insulin signaling and worsen IR [3, 4]. Increasing evidence also suggests that dysregulated lipid and glucose metabolism can aggravate chronic inflammation, which in return exacerbates metabolic disorders, forming a vicious cycle. Elevated blood low‐density lipoprotein cholesterol (LDL‐C) and glucose levels can trigger inflammatory responses, whereas inflammation‐induced cytokines suppress high‐density lipoprotein cholesterol (HDL‐C) expressions and impair reverse cholesterol transport [5–7]. Proinflammatory cytokines such as tumor necrosis factor‐*α* (TNF‐*α*) and interleukin‐6 (IL‐6) can also disrupt insulin signaling, further promoting IR [8–9]. Collectively, these findings highlight the need for safe and effective interventions that can simultaneously modulate metabolic and inflammatory pathways.

Coenzyme Q10 (CoQ10) is an essential component of mitochondrial oxidative phosphorylation and a lipid‐soluble antioxidant that protects cell membranes and circulating lipoproteins [10, 11]. CoQ10 exists in two interconvertible forms: ubiquinone (oxidized) and ubiquinol (reduced). During or after intestinal absorption, ubiquinone is readily reduced to ubiquinol, which accounts for more than 95% of circulating CoQ10. Therefore, its physiological functions are not substantially influenced by the form in which it is consumed [12, 13]. Although CoQ10 is synthesized endogenously, its levels decline with age and in several metabolic conditions [11, 14]. Besides, it can be obtained from various foods. The primary dietary sources of CoQ10 include meat, fish, and certain oils. Among these, organ meats such as heart and liver provide the highest concentrations, typically exceeding 100 mg/kg, followed by fatty fishes such as herrings and sardines, as well as vegetable oils including soybean and corn oil. In contrast, most dairy products, vegetables, and fruits contain substantially lower amounts, usually less than 5 mg/kg [13]. Typical dietary intake in adults is approximately 3–6 mg/day and may be insufficient to offset CoQ10 depletion associated with aging or metabolic disorders [13, 15]. Exogenous CoQ10 supplementation has been reported to exert antioxidant and anti‐inflammatory effects and may improve lipid metabolism and insulin sensitivity, supporting its potential role in individuals with dyslipidemia or impaired glucose metabolism [16, 17].

Previous meta‐analyses have mostly focused on isolated outcomes (e.g., lipid profile [18, 19], glycemic indices [20], or inflammatory markers [21–24]) or specific populations (e.g., cardiovascular disease) [24]. In contrast, we conducted a comprehensive systematic review and meta‐analysis across broader populations and evaluated multiple biochemical domains, including lipid markers (triglyceride [TG], total cholesterol [TC], HDL‐C, and LDL‐C) [25], glycemic outcomes (hemoglobin A1C [HbA1c], fasting glucose, fasting insulin [FINS], and homeostasis model assessment–insulin resistance [HOMA‐IR]) [26, 27], and inflammatory biomarkers (TNF‐*α*, IL‐6, and C‐reactive protein [CRP]) [28]. By integrating these outcomes, our meta‐analysis is aimed at providing an overall assessment of the metabolic and inflammatory effects of CoQ10 supplementation to inform clinical practice and future research.

## 2. Methods

This systematic review was reported in accordance with the Preferred Reporting Items for Systematic Reviews and Meta‐Analysis (PRISMA) 2020 statement [29]. The study protocol was prospectively registered in PROSPERO (CRD42022313682). Deviations from the registered protocol are described in Supporting File S1.

### 2.1. Data Sources and Search Strategy

We searched PubMed, Web of Science, Scopus, Embase, Cochrane Library, and ClinicalTrials.gov from inception to January 2026. The language was limited to English. A combination of “coenzyme Q10,” “glucose,” “lipid”, “triacylglycerol,” “cholesterol,” “insulin,” “glycated hemoglobin,” “hemoglobin A1c protein,” “inflammation,” “interleukin‐6,” “tumor necrosis factor‐alpha,” and “C‐reactive protein” was applied for search, using both free words and index terms. Further manual retrieval of the reference lists of the included studies and relevant meta‐analyses was conducted to identify additional eligible studies. Full search strategies are available in the Supporting File S2.

### 2.2. Eligibility Criteria and Selection Process

Studies were included if they met the following criteria: (1) randomized controlled trials (RCTs) evaluating CoQ10 alone or CoQ10 plus another therapy (any dose, formulation, or duration) compared with placebo, no treatment, or the same cointervention without CoQ10; (2) adults with disorders of lipid and/or glucose metabolism; and (3) reporting at least one of the primary outcomes, including TG, TC, HDL‐C, LDL‐C, HbA1c, fasting glucose, FINS, HOMA‐IR, CRP, TNF‐*α*, and IL‐6. Studies were excluded for the following reasons: (1) review articles, commentaries, case reports, observational studies, retrospective studies, nonclinical trials, and cell or animal studies; and (2) pregnant or lactating women, healthy individuals, or individuals without metabolic disorders.

Two reviewers independently screened titles and abstracts and then assessed full texts for eligibility. Disagreements were resolved by discussion or consultation with another author.

### 2.3. Data Extraction

Two reviewers independently extracted data using a standardized, prespecified form. Discrepancies were resolved by discussion and consultation with a third reviewer. Extracted items included: (1) study characteristics (first author, year, region, funding, and design); (2) participant characteristics (health status, body mass index [BMI], sex, age, and sample size per group); (3) intervention details (CoQ10 form, dose, cointerventions, and duration); and (4) outcomes (mean change from baseline or baseline and endpoint means with standard deviations [SDs]) and units for intervention and control groups. The missing or uncalculable data were initially examined for possible derivation using reported statistical information. When the required parameters could not be calculated, those datasets were excluded from the quantitative synthesis but retained for qualitative reference. For multiarm parallel studies, each arm was treated as an independent comparison, and cross‐over design studies were treated similarly. When outcomes were reported at multiple time points, we extracted data corresponding to the end of the intervention.

### 2.4. Data Analysis

Data analyses were conducted in Stata software (Version 16.0). For continuous variables, pooled effect size estimates were expressed as weighted mean difference (WMD) or standardized mean difference (SMD) with 95% confidence intervals (CIs). *p* values were used to measure statistical differences, and *p* < 0.05 was considered statistically significant. When mean changes were not reported, SDs were calculated from baseline and endpoint values. Given expected clinical and methodological heterogeneity, we applied the random‐effects inverse‐variance model with the DerSimonian–Laird estimator of tau^2^. Duplicate data were excluded.

Subgroup analyses explored heterogeneity by: region (Asia, Europe, Oceania, or North America), intervention type (CoQ10, CoQ10 plus statin, CoQ10 plus fenofibrate, CoQ10 plus another adjunct therapy, or CoQ10 plus exercise), dosage (< 100, ≥ 100 and < 200, ≥ 200 and < 300, or ≥ 300 mg/day), duration (< 12 or ≥ 12 weeks), baseline BMI (≥ 18.5 and < 25, ≥ 25 and < 30, or ≥ 30 kg/m^2^), age (< 45, ≥ 45 and < 60, or ≥ 60 years), disease, and baseline lipid levels. Given the exploratory nature of these analyses, *p* values were not adjusted for multiple comparisons.

Heterogeneity was assessed using Cochran′s Q test and I^2^ index. Meta‐regression analyses were performed to examine potential moderators (dosage, duration, baseline BMI, age, and lipid baseline). Results were interpreted as exploratory. Robustness was evaluated using leave‐one‐out sensitivity analyses. Publication bias was assessed by visual inspection of funnel plots and Egger′s and Begg′s tests. The trim and fill methods would be used if there was any potential risk of publication bias.

### 2.5. Certainty Assessment

The certainty of the evidence was assessed according to the Grading of Recommendations, Assessment, Development, and Evaluation (GRADE) method, using GRADEpro Guideline Development Tool [30]. Certainty was rated as high, moderate, low, or very low based on risk of bias, inconsistency, indirectness, imprecision, and other considerations.

### 2.6. Risk of Bias Assessment

Risk of bias was assessed using the Cochrane risk‐of‐bias tool for randomized trials, Version 2 (RoB 2) [31]. Each article was evaluated based on the following domains: randomization process, deviations from intended interventions, missing outcome data, measurement of the outcome, and selection of the reported result. Judgment can be “low” or “high” risk of bias, or can express “some concerns.”

## 3. Results

### 3.1. Search Results

**Figure 1:**
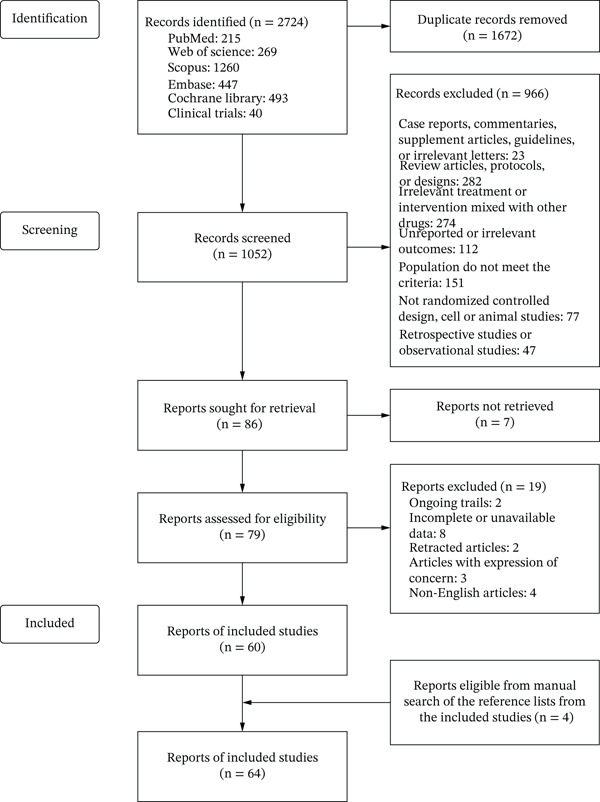
Flow diagram for this meta‐analysis.

A total of 2724 records were identified from PubMed, Web of Science, Scopus, Embase, Cochrane Library, and ClinicalTrials.gov. After removing duplicates, 1052 records were screened by title and abstract, and 966 were excluded. The full texts of 79 reports were assessed for eligibility, of which 60 met the inclusion criteria. Additional four eligible reports were identified by manually screening reference lists of included studies and relevant meta‐analyses. In total, 64 reports were included. Figure 1 shows the detailed flow diagram of selection processes.

### 3.2. Characteristics of Included Studies

A total of 74 arms from 64 reports were included, involving 3422 participants. Mean age ranged from 24.5 to 79.2 years, and mean BMI ranged from 23 to 36.44 kg/m^2^. The studies were published between 1994 and 2026 and were conducted in North America (*n* = 3) [32–34], Oceania (*n* = 6) [35–40], Europe (*n* = 14) [41–54], and Asia (*n* = 41) [55–95], with Iran contributing the largest number (*n* = 24). Participants had various metabolic disorders, including dyslipidemia (*n* = 16), diabetes or impaired glucose tolerance (IGT) (*n* = 23), polycystic ovary syndrome (PCOS) (*n* = 6), CAD (*n* = 6), metabolic syndrome (*n* = 1), nonalcoholic fatty liver disease (NAFLD) (*n* = 4), and other comorbid conditions (*n* = 8). CoQ10 doses ranged from 60 to 600 mg/day, and intervention duration ranged from 4 to 24 weeks. Most trials used ubiquinone; two trials used ubiquinol [67, 77]. In addition to CoQ10 monotherapy, several trials evaluated CoQ10 combined with other interventions (e.g., statins, fenofibrate, and vitamin E). Table 1 outlines the characteristics of the trials identified in our analysis.

**Table 1:** Study characteristics of trials included in the analysis.

Study, year (country)	Design	Population	*n*(I/C)	BMI, kg/m^2^ (I/C)	Age, years (I/C)	Intervention	Dose (mg/day)	Duration (weeks)	Industry funding	Outcomes
AbdulKareem, 2022 (Iraq)	Randomized, open‐label, parallel	Diabetic neuropathy	17/16	NA	NA	CoQ10+gabapentin	200	12	N	HbA1c, fasting glucose, TNF‐*α*, IL‐6
Aldean, 2026 (Iraq)	Randomized, single‐blind, parallel	PCOS	30/30	27.90 ± 1.70/27.70 ± 1.37	30.90 ± 3.12/30.33 ± 4.06	CoQ10+metformin and alpha‐lipoic acid	200	12	N	TG, TC, HDL‐C, LDL‐C
Aljawad, 2015 (Iraq)	Randomized, parallel	T2DM and hyperlipidemia	14/13	NA	NA	CoQ10	150	12	N	TG, TC, HDL‐C, LDL‐C, HbA1c, fasting glucose
Andersen, 1997 (Denmark)	Randomized, double‐blind, parallel	DM	17/17	23.5 ± 0.7/24.0 ± 0.6	^∗^35 ± 2.0/35.3 ± 2.4	CoQ10	100	12	N	HDL‐C, LDL‐C, HbA1c, fasting glucose
Bader, 2022 (Iraq)	Randomized, double‐blind, parallel	PCOS	50/50	29.14 ± 6.96/30.10 ± 5.40	NA	CoQ10	200	12	N	TG, TC, HDL‐C, LDL‐C, HbA1c, fasting glucose
Bargossi, 1994 (Italy)	Randomized, crossover	Hypercholesterolemia	15/15	NA	NA	CoQ10+simvastatin	100	12	Y	TC
Belardinelli, 2006I (Italy)	Randomized, double‐blind, crossover	CAD	23/23	NA	59 ± 9	CoQ10	300	4	N	TG, TC, HDL‐C, LDL‐C
Belardinelli, 2006II (Italy)	Randomized, double‐blind, crossover	CAD	23/23	NA	59 ± 9	CoQ10+exercise	300	4	N	TG, TC, HDL‐C, LDL‐C
Chew, 2008I (Australia)	Randomized, double‐blind, parallel	T2DM	16/20	30.1 ± 4.6/30.7 ± 5.0	61.3 ± 4.1/62.4 ± 8.8	CoQ10	200	24	Y	TG, TC, HDL‐C, LDL‐C, HbA1c, fasting glucose
Chew, 2008II (Australia)	Randomized, double‐blind, parallel	T2DM	19/19	28.7 ± 3.4/29.9 ± 5.6	63.0 ± 9.4/64.8 ± 7.3	CoQ10+fenofibrate	200	24	Y	TG, TC, HDL‐C, LDL‐C, HbA1c, fasting glucose
Dai, 2011 (China)	Randomized, double‐blind, parallel	CAD	28/28	25.3 ± 3.2/24.7 ± 3.2	67.7 ± 9.4/70.1 ± 9.8	CoQ10	300	8	Y	TG, TC, HDL‐C, LDL‐C, HbA1c, fasting glucose, CRP
Derosa, 2019 (Italy)	Randomized, double‐blind, parallel	Dyslipidemia	29/27	27.2 ± 1.8/27.5 ± 2.0	59.8 ± 8.3/58.3 ± 7.9	CoQ10	100	12	Y	TG, TC, HDL‐C, LDL‐C
Eriksson, 1999 (Denmark)	Randomized, double‐blind, parallel	T2DM	12/11	29.0 ± 4.2/29.8 ± 3.4	65 ± 5/64 ± 7	CoQ10	200	24	Y	TG, TC, HDL‐C, HbA1c, fasting glucose
Fakhrabadi, 2014 (Iran)	Randomized, double‐blind, parallel	Diabetic neuropathy	32/30	28.7 ± 4.1/29.6 ± 3.1	56.7 ± 6.4/54.8 ± 6.7	CoQ10	200	12	N	TC, HDL‐C, LDL‐C, fasting glucose, FINS, HOMA‐IR, CRP
Fallah, 2019 (Iran)	Randomized, double‐blind, parallel	Diabetic hemodialysis	30/30	NA	64.8 ± 11.5/59.4 ± 12.2	CoQ10	120	12	N	CRP
Farhangi, 2014 (Iran)	Randomized, double‐blind, parallel	NAFLD	20/21	30.59 ± 3.98/28.75 ± 4.02	42.73 ± 10.77/42.18 ± 10.80	CoQ10	100	4	N	Fasting glucose, FINS, HOMA‐IR
Farsi, 2016 (Iran)	Randomized, double‐blind, parallel	NAFLD	20/21	28.23 ± 3.60/29.69 ± 5.76	NA	CoQ10	100	12	N	CRP, TNF‐*α*, IL‐6
Gholami, 2018 (Iran)	Randomized, double‐blind, parallel	T2DM	34/34	29.44 ± 0.60/28.53 ± 0.53	53.35 ± 6.56/53.1 ± 6.23	CoQ10	100	12	N	TG, TC, HDL‐C, LDL‐C, HbA1c, fasting glucose, FINS, HOMA‐IR
Gholami, 2019 (Iran)	Randomized, double‐blind, parallel	T2DM	35/35	29.30 ± 0.6/28.51 ± 0.52	52.97 ± 1.04/53.68 ± 1.14	CoQ10	100	12	N	TG, TC, HDL‐C, LDL‐C, HbA1c, fasting glucose, FINS, HOMA‐IR, CRP
Hamilton, 2009 (Australia)	Randomized, double‐blind, crossover	Statin‐treated T2DM	23/23	29 ± 4	68 ± 6	CoQ10	200	12	Y	LDL‐C, HbA1c
Hansen, 2019 (Denmark)	Randomized, double‐blind, parallel	Hypercholesterolemia	18/17	28 ± 1/29 ± 1	62 ± 1/64 ± 2	CoQ10	400	8,9	Y	TG, TC, HDL‐C, LDL‐C, CRP, TNF‐*α*, IL‐6
Henriksen, 1999 (Denmark)	Randomized, double‐blind, parallel	T1DM	17/17	23.5 ± 2.7/24.0 ± 2.6	35.5 ± 8.2/35.3 ± 10.0	CoQ10	100	12	Y	TG, TC, HDL‐C, LDL‐C, HbA1c, fasting glucose
Hernandez‐Ojeda, 2012 (Mexico)	Randomized, double‐blind, parallel	Diabetic polyneuropathy	24/25	29.4 ± 7.3/29.3 ± 4.3	55.3 ± 8.4/57.0 ± 8.9	CoQ10	400	12	N	TG, TC, HDL‐C, LDL‐C, HbA1c, fasting glucose
Hodgson, 2002I (Australia)	Randomized, double‐blind, parallel	T2DM and dyslipidemia	19/18	NA	^∗∗^52.3 ± 1.4/55.2 ± 2.3	CoQ10	200	12	N	TG, TC, HDL‐C, LDL‐C, HbA1c, fasting glucose, FINS
Hodgson, 2002II (Australia)	Randomized, double‐blind, parallel	T2DM and dyslipidemia	19/18	NA	^∗∗^51.7 ± 1.6/53.6 ± 2.4	CoQ10+fenofibrate	200	12	N	TG, TC, HDL‐C, LDL‐C, HbA1c, fasting glucose, FINS
Hosseinzadeh‐Attar, 2015 (Iran)	Randomized, double‐blind, parallel	T2DM	31/33	29.52±2.8/29.47 ± 3.24	45.2 ± 7.6/47.1 ± 8.3	CoQ10	200	12	N	TG, TC, HDL‐C, LDL‐C, HbA1c, fasting glucose
Izadi, 2018I (Iran)	Randomized, double‐blind, parallel	PCOS	22/21	28.97 ± 2.95/28.73 ± 3.39	27.64±5.2/26.0±4.53	CoQ10	200	8	N	Fasting glucose, FINS, HOMA‐IR
Izadi, 2018II (Iran)	Randomized, double‐blind, parallel	PCOS	21/22	29.28 ± 3.23/29.28 ± 4.24	28.33 ± 5.52/27.18 ± 5.77	CoQ10+VitaminE	200	8	N	Fasting glucose, FINS, HOMA‐IR
Izadi, 2019I (Iran)	Randomized, double‐blind, parallel	PCOS	22/21	28.97 ± 2.95/28.73 ± 3.39	27.64 ± 5.2/26.0 ± 4.53	CoQ10	200	8	Y	TG, TC, HDL‐C, LDL‐C
Izadi, 2019II (Iran)	Randomized, double‐blind, parallel	PCOS	21/22	29.28 ± 3.23/29.28 ± 4.24	28.33 ± 5.52/27.18 ± 5.77	CoQ10+VitaminE	200	8	Y	TG, TC, HDL‐C, LDL‐C
Jafarvand, 2016 (Iran)	Randomized, double‐blind, parallel	NAFLD	20/21	30.5 ± 3.9/28.7 ± 4.02	42.7 ± 10.2/42.2 ± 10.8	CoQ10	100	4	N	TG, TC, HDL‐C, LDL‐C
Kaikkonen, 2000I (Finland)	Randomized, double‐blind, parallel	Dyslipidemia	10/10	NA	NA	CoQ10	200	12	Y	TG, TC, HDL‐C, LDL‐C
Kaikkonen, 2000II (Finland)	Randomized, double‐blind, parallel	Dyslipidemia	10/10	NA	NA	CoQ10+oil‐based‐d‐*α*‐tocopherol	200	12	Y	TG, TC, HDL‐C, LDL‐C
Karamali, 2021 (Iran)	Randomized, double‐blind, parallel	PCOS	28/27	27.2 ± 3.9/28.2 ± 4.8	27.1 ± 6.5/29.0 ± 6.9	CoQ10	100	12	N	CRP
Kuhlman, 2019 (Denmark)	Randomized, double‐blind, parallel	Dyslipidemia	18/17	27.7 ± 0.6/28.8 ± 0.7	62 ± 1/64 ± 2	CoQ10	400	8	Y	TG, TC, HDL‐C, LDL‐C, HbA1c, fasting glucose, FINS
Kuhlman, 2022 (Denmark)	Randomized, double‐blind, parallel	Dyslipidemia	18/17	28.6 ± 2.4/27.5 ± 2.2	56 ± 8/56 ± 9	CoQ10+simvastatin	400	8	Y	TG, TC, HDL‐C, LDL‐C, HbA1c, fasting glucose, FINS
Lankin, 2000 (Russian)	Randomized, double‐blind, parallel	Dyslipidemia and CAD	9/10	NA	49 ± 2.5	CoQ10+Pravastatin	60	24	N	TC, HDL‐C, LDL‐C
Lee, 2012I (China)	Randomized, double‐blind, parallel	CAD	14/12	25.6 ± 2.8/26.9 ± 2.8	75.1 ± 4.9/77.2 ± 7.4	CoQ10	60	12	N	CRP, IL‐6
Lee, 2012II (China)	Randomized, double‐blind, parallel	CAD	14/12	25.2 ± 2.6/26.9 ± 2.8	79.2 ± 5.4/77.2 ± 7.4	CoQ10	150	12	N	CRP, IL‐6
Lee, 2013 (China)	Randomized, double‐blind, parallel	CAD	23/19	25.9 ± 3.5/26.7 ± 3.2	71.7 ± 11.5/66.5 ± 11.1	CoQ10	300	12	N	CRP, TNF‐*α*, IL‐6, TGTC
Lim, 2007 (Singapore)	Randomized, double‐blind, parallel	T2DM	40/40	24.8 ± 5.5/25.2 ± 3.4	54 ± 9/53 ± 9	CoQ10	200	12	Y	HbA1c, fasting glucose
Mabuchi, 2007 (Japan)	Randomized, double‐blind, parallel	Hypercholesterolemia	24/25	23.3 ± 2.7/23.9 ± 3.4	61 ± 8/60 ± 8	CoQ10+atorvastatin	100	12	Y	TG, TC, HDL‐C, LDL‐C, CRP
Mehrdadi, 2017 (Iran)	Randomized, double‐blind, parallel	T2DM	26/30	29.68 ± 2.92/29.31 ± 3.26	46 ± 7/48 ± 8	CoQ10	200	12	N	HbA1c, fasting glucose, FINS, HOMA‐IR
Mirhashemi, 2016 (Iran)	Randomized, double‐blind, parallel	T2DM with stable MI	30/30	28.2 ± 5.2/30.7 ± 5.9	65.9 ± 12.5/59.9 ± 13.1	CoQ10	100	8	N	IL‐6
Moazen, 2015 (Iran)	Randomized, single‐blind, parallel	T2DM	26/26	25.31 ± 2.14/25.34 ± 2.39	50.67 ± 7.01/52.79 ± 7.66	CoQ10	200	8	Y	HbA1c, fasting glucose, TGTC
Mohammadshahi, 2014 (Iran)	Randomized, double‐blind, parallel	NAFLD	20/21	28.23 ± 3.60/29.69 ± 5.76	NA	CoQ10	100	12	N	TG, TC, HDL‐C, LDL‐C, fasting glucose
Mohseni, 2014 (Iran)	Randomized, double‐blind, parallel	Hyperlipidemia with MI	26/26	25.91 ± 2.53/26 ± 3.34	60 ± 8/61 ± 7	CoQ10	200	12	N	
Mohseni, 2015 (Iran)	Randomized, double‐blind, parallel	Hyperlipidemia with MI	26/26	25.91 ± 2.53/26 ± 3.34	60 ± 8/61 ± 7	CoQ10	200	12	N	TG, TC, HDL‐C, LDL‐C, IL‐6
Noor, 2014 (Iraq)	Randomized, parallel	T2DM	19/19	28.15±4.08/29.5±4.29	49.37±6.65/51.63±8.13	CoQ10	150	8	N	TC, HDL‐C, LDL‐C, HbA1c, fasting glucose
Palomaki, 1998 (Finland)	Randomized, double‐blind, crossover	Dyslipidemia and CAD	19/19	27.2 ± 0.7	55 ± 7	CoQ10+lovastatin	180	6	Y	TG, TC, HDL‐C, LDL‐C
Pek, 2016 (Singapore)	Randomized, double‐blind, parallel	Hypercholesterolemia	20/20	29.2 ± 3.8/31.2 ± 6.1	43.1 ± 11.3/49.2 ± 12.2	Ubiquinol+simvastatin	150	12	N	TG, TC, HDL‐C, LDL‐C
Pekarova, 2017 (Slovakia)	Randomized, double‐blind, parallel	Dyslipidemia	35/35	27.89 ± 6.01/29.31 ± 4.13	58.4 ± 13.8/61.96 ± 12.2	CoQ10+omega‐3 PUFA	200	12	N	TC, HDL‐C, LDL‐C, fasting glucose, CRP, IL‐6
Playford, 2003I (Australia)	Randomized, double‐blind, parallel	Dyslipidemic T2DM	20/20	^∗∗^29.9 ± 0.7/30.9 ± 1.0	^∗∗^52.7 ± 1.4/54.7 ± 2.1	CoQ10	200	12	Y	TG, TC, HDL‐C, LDL‐C, HbA1c
Playford, 2003II (Australia)	Randomized, double‐blind, parallel	Dyslipidemic T2DM	20/20	^∗∗^30.3 ± 0.9/30.0 ± 0.8	^∗∗^52.7 ± 1.8/53.5 ± 2.2	CoQ10+fenofibrate	200	12	Y	TG, TC, HDL‐C, LDL‐C, HbA1c
Raitakari, 2000 (Australia)	Randomized, double‐blind, crossover	Dyslipidemia	12/12	23 ± 4	34 ± 10	CoQ10	150	4	Y	LDL‐C
Rodriguez‐Carrizalez, 2016 (Mexico)	Randomized, double‐blind, parallel	T2DM	20/20	28.2 ± 3.7/29.3 ± 0.8	58.5 ± 1.9/57.8 ± 1.9	CoQ10	400	24	Y	TG, TC, HDL‐C, LDL‐C, HbA1c, fasting glucose
Sangouni, 2022I (Iran)	Randomized, double‐blind, parallel	Metabolic syndrome	22/22	30.7 ± 4.9/30.0 ± 4.7	39.0 ± 4.5/39.5 ± 5.0	CoQ10	60	12	N	TG, TC, HDL‐C, LDL‐C, fasting glucose
Sangouni, 2022II (Iran)	Randomized, double‐blind, parallel	Metabolic syndrome	22/22	29.7 ± 5.1/30.0 ± 4.6	37.7 ± 5.0/38.8 ± 4.9	CoQ10+curcumin	60	12	N	TG, TC, HDL‐C, LDL‐C, fasting glucose
Sedeh, 2018 (Iran)	Randomized, double‐blind, parallel	T2DM	34/34	NA	60.2 ± 11.4/54.3 ± 9.6	CoQ10	100	8	N	HbA1c, fasting glucose
Shahraki, 2021 (Iran)	Randomized, double‐blind, parallel	Diabetic ESRD	20/20	NA	60.2 ± 8.5/60.4 ± 8.2	CoQ10	200	24	N	CRP
Shojaei, 2011I (Iran)	Randomized, double‐blind, parallel	Maintenance hemodialysis patients	13/13	23.6 ± 2.4/23.6 ± 2.6	53.3 ± 11.5/51.6 ± 19.2	CoQ10	100	12	N	TG, TC, HDL‐C, LDL‐C
Shojaei, 2011II (Iran)	Randomized, double‐blind, parallel	Maintenance hemodialysis patients	22/22	23.3 ± 2.3/24.3 ± 2.1	52.8 ± 10.4/55.3 ± 15.6	CoQ10+intravenous carnitine	100	12	N	TG, TC, HDL‐C, LDL‐C
Singh, 1999 a (India)	Randomized, double‐blind, parallel	CAD	30/29	23.9 ± 1.2/23.8 ± 1.1	48.3 ± 7.2/48.0 ± 7.0	CoQ10	120	8	Y	TG, HDL‐C, fasting glucose, FINS
Singh, 1999 b (India)	Randomized, double‐blind, parallel	CAD	25/22	23.6 ± 1.2/23.5 ± 1.2	48.4 ± 0.5/47.6 ± 0.3	CoQ10	120	4	Y	TC, HDL‐C, LDL‐C, fasting glucose
Skarlovnik, 2014 (Slovenia)	Randomized, double‐blind, parallel	Statin‐related muscle pain	25/25	25.3 ± 1.2/24.6 ± 1.5	64.5 ± 1.9/65.6 ± 2.1	CoQ10	100	4	N	TG, TC, LDL‐C
Taghizadeh, 2020 (Iran)	Randomized, double‐blind, parallel	PCOS	22/21	28.97 ± 2.95/28.73 ± 3.16	27.64 ± 5.19/26.00 ± 4.53	CoQ10	200	8	N	CRP, TNF‐*α*, IL‐6
Taylor, 2015 (USA)	Randomized, double‐blind, crossover	Statin myalgia	20/18	NA	58 ± 10/60 ± 10	CoQ10+simvastatin	600	8	N	LDL‐C
Yasser, 2021 (Iraq)	Randomized, open‐label, parallel	Dyslipidemia	28/24	32.54 ± 4.66/31.59 ± 5.08	59.24 ± 5.571/58.11 ± 7.10	CoQ10+atorvastatin	200	12	N	TG, TC, HDL‐C, LDL‐C, fasting glucose, IL‐6
Yen, 2018 (China)	Randomized, double‐blind, parallel	T2DM	24/23	28.0 ± 4.8/27.3 ± 3.4	61.5 ± 10.2/59.6 ± 11.7	Ubiquinol	100	12	Y	TC, HDL‐C, LDL‐C, HbA1c, fasting glucose, FINS, HOMA‐IR
Yoo, 2018 (Korea)	Randomized, double‐blind, parallel	Impaired glucose tolerance	39/39	26.7 ± 3.7/26.3 ± 3.4	49.79 ± 8.4/52.44 ± 6.9	CoQ10	200	8	Y	HbA1c, fasting glucose, FINS, HOMA‐IR
Young, 2007 (New Zealand)	Randomized, double‐blind, parallel	Simvastatin‐induced myalgia	22/22	NA	^∗∗^59 ± 2/59 ± 2	CoQ10+simvastatin	200	12	N	TG, TC, HDL‐C, LDL‐C
Zahedi, 2014 (Iran)	Randomized, double‐blind, parallel	T2DM	20/20	NA	53.5 ± 9.7/58.8 ± 9.6	CoQ10	150	12	N	TG, TC, HDL‐C, LDL‐C, HbA1c, fasting glucose, FINS, HOMA‐IR
Zarei, 2018 (Iran)	Randomized, double‐blind, parallel	T2DM	34/34	36.44 ± 0.57/32.56 ± 0.61	53.1 ± 6.23/53.35 ± 6.56	CoQ10	100	12	N	HbA1c, fasting glucose, FINS
Zhang, 2018 (China)	Randomized, double‐blind, parallel	Dyslipidemia	51/50	25.23 ± 3.96/24.91 ± 3.32	51.78 ± 8.92/50.02 ± 10.91	CoQ10	120	24	Y	TG, TC, HDL‐C, LDL‐C, fasting glucose, FINS, HOMA‐IR, CRP

*Note:* Data presentation: Values are mean ± standard deviation unless otherwise indicated. Single asterisk “∗” denotes age reported as 3.5 ± 2.0 in original article was corrected to 35 ± 2.0; and double asterisks “ ^∗∗^” denote values reported as mean ± SEM in the original article.

Abbreviations: BMI, body mass index; C, control group; CAD, coronary artery disease; CoQ10, coenzyme Q10; ESRD, end‐stage renal disease; I, intervention group; MI, myocardial infarction; N, not funded by industry; NA, not available; NAFLD, nonalcoholic fatty liver disease; PCOS, polycystic ovary syndrome; T1DM/T2DM, type 1/2 diabetes mellitus; Y, funded by industry.

### 3.3. Effects of CoQ10 Supplementation on Lipid Profiles

#### 3.3.1. TG

**Figure 2:**
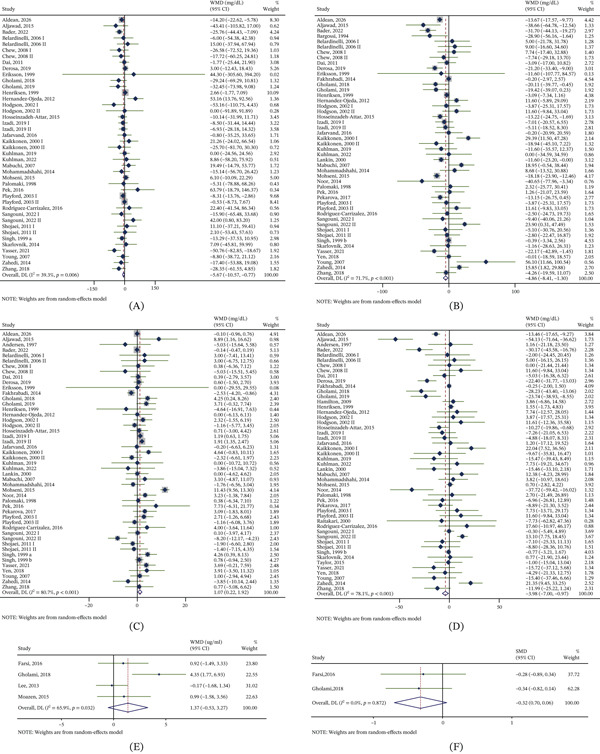
Forest plots of effects of coenzyme Q10 supplementation on lipid profiles. Data from randomized controlled trials were analyzed using a random‐effects model and are presented as WMDs with 95% CIs for (A) triglyceride (*n* = 1861), (B) total cholesterol (*n* = 2115), (C) high‐density lipoprotein cholesterol (*n* = 2128), (D) low‐density lipoprotein cholesterol (*n* = 2205), and (E) adiponectin (*n* = 203), and SMD with 95% CI was calculated for (F) leptin (*n* = 109). Each square represents an individual study with its corresponding weight, and horizontal lines denote 95% CIs. The diamond indicates the pooled effect size. Statistical significance was considered when the 95% CI did not cross the vertical line of no effect. Abbreviations: WMD, weighted mean difference; CI, confidence interval; SMD, standardized mean difference.

Forty‐two trial arms (933 participants in the CoQ10 groups and 928 in the control groups) showed that CoQ10 supplementation significantly reduced serum TG, with low‐to‐moderate heterogeneity. (WMD = −5.67 mg/dL; 95% CI: −10.57, −0.77; *I* ^2^ = 39.3*%*; *p* = 0.006) (Figure 2A). In subgroup analyses, TG reductions were significant in trials enrolling individuals with PCOS; trials conducted in Asia or Oceania; trials using doses of ≥ 200 and < 300 mg/day; trials with duration ≥ 12 weeks; and trials evaluating CoQ10 alone (Table 2).

**Table 2:** Subgroup analysis for primary outcome indicators.

Study groups		No. of trials	Participants (experimental/control)	Effects		Heterogeneity		
				WMD or SMD (95% CI)	*p*effect	I^2^	*p*heterogeneity	*p*between subgroups
**TG (mg/dL)**								
Overall		42	933/928	−5.67 (−10.57, −0.77)	0.023	39.3%	0.006	
Disease	Dyslipidemia and diabetes	5	90/84	−6.66 (−15.33, 2.01)	0.132	37.8%	0.169	0.369
	PCOS	4	123/123	−14.50 (−21.38, −7.61)	0.000	0.0%	0.541	
	CAD	4	100/99	−5.38 (−20.69, 9.92)	0.490	0.0%	0.785	
	Diabetes	10	226/231	−5.44 (−19.43, 8.55)	0.446	0.5%	0.057	
	Dyslipidemia	13	265/260	−3.34 (−15.94, 9.26)	0.604	33.5%	0.114	
	NAFLD	2	40/42	−6.64 (−33.16, 19.88)	0.624	0.0%	0.603	
	Dyslipidemia and CAD	2	45/45	5.57 (−10.24, 21.39)	0.490	0.0%	0.767	
	Metabolic syndrome	2	44/44	14.76 (−41.88, 71.40)	0.610	0.7%	0.078	

Region	Asia	22	567/566	−10.18 (−17.61, −2.75)	0.007	34.4%	0.058	0.000
	Europe	11	191/188	2.65 (−1.46, 6.76)	0.206	0.0%	0.995	
	Oceania	7	131/129	−6.56 (−11.04, −2.08)	0.004	0.0%	0.421	
	North America	2	44/45	44.70 (11.15, 78.24)	0.009	0.0%	0.422	

Dosage (mg/day)	< 100	2	44/44	14.76 (−41.88, 71.40)	0.610	67.7%	0.078	0.075
	≥ 100 and < 200	16	385/381	−2.15 (−9.06, 4.76)	0.542	11.0%	0.328	
	≥ 200 and < 300	17	363/361	−9.78 (−15.56, −4.00)	0.001	32.6%	0.096	
	≥ 300	7	141/142	8.64 (−6.39, 23.67)	0.260	10.3%	0.350	

Duration (weeks)	≥ 12	31	699/694	−6.35 (−12.51, −0.20)	0.043	54.4%	0.000	0.735
	< 12	11	234/234	−4.43 (−13.70, 4.83)	0.348	0.0%	0.998	

Intervention	CoQ10	29	673/671	−5.63 (−11.24, −0.02)	0.049	46.8%	0.111	0.824
	CoQ10+statin	5	105/105	−4.02 (−37.70, 29.66)	0.815	67.0%	0.016	
	CoQ10+fenofibrate	2	37/35	−0.53 (−8.69, 7.64)	0.900	0.0%	0.991	
	CoQ10+another adjunct	5	97/96	−4.64 (−21.72, 12.45)	0.595	46.8%	0.111	
	CoQ10+exercise	1	21/21	15.00 (−37.94, 67.94)	0.579	—	—	

Baseline BMI (kg/m^2^)	≥ 18.5 and < 25	6	123/121	2.52 (−1.76, 6.79)	0.248	0.0%	0.741	0.050
	≥ 25 and < 30	21	538/538	−5.61 (−13.06,1.83)	0.140	36.7%	0.048	
	≥ 30	6	116/116	−10.17 (−22.48, 2.14)	0.105	62.7%	0.020	
	NA	9	156/153	−11.04 (−26.56, 4.47)	0.163	0.0%	0.587	

Age (years)	< 45	7	154/155	−3.39 (−14.04, 7.26)	0.532	65.3%	0.008	0.150
	≥ 45 and < 60	22	509/501	−7.02 (−14.50, 0.45)	0.066	35.9%	0.049	
	≥ 60	8	166/168	1.81 (−8.45, 12.07)	0.729	0.0%	0.806	
	NA	5	104/104	−19.81 (−35.84, −3.79)	0.015	5.2%	0.377	

Baseline TG (mg/dL)	< 150	16	362/364	0.43 (−3.33, 4.19)	0.823	0.0%	0.491	0.056
	≥ 150	26	571/564	−7.45 (−14.59, −0.30)	0.041	43.3%	0.011	

**TC (mg/dL)**								
Overall		48	1063/1052	−4.86 (−8.41, −1.30)	0.007	71.7%	0.000	
Disease	Dyslipidemia and diabetes	5	90/84	−3.61 (−19.91, 12.69)	0.665	62.7%	0.030	0.005
	PCOS	4	123/123	−14.53 (−24.12, −4.93)	0.003	71.5%	0.015	
	Dyslipidemia	15	315/310	−3.54 (−13.05, 5.98)	0.466	67.3%	0.000	
	CAD	4	95/92	−0.33 (−3.18, 2.53)	0.823	0.0%	0.846	
	Diabetes	13	301/303	−3.05 (−8.15, 2.05)	0.242	52.3%	0.01	
	NAFLD	2	40/42	3.95 (−11.23, 19.12)	0.610	0.0%	0.567	
	Dyslipidemia and CAD	3	55/54	−14.92 (−22.32, −7.53)	0.000	26.9%	0.255	
	Metabolic syndrome	2	44/44	8.75 (−23.75, 41.25)	0.598	64.9%	0.092	

Region	Asia	25	637/630	−6.92 (−11.64, −2.20)	0.004	79.9%	0.000	0.086
	Europe	14	251/248	−5.67 (−13.13, 1.79)	0.136	57.8%	0.004	
	Oceania	7	131/129	5.15 (−5.47, 15.77)	0.342	29.5%	0.203	
	North America	2	44/45	6.21 (−7.53, 19.96)	0.376	0.0%	0.329	

Dosage (mg/day)	< 100	3	54/54	0.24 (−22.90, 23.39)	0.984	71.8%	0.029	0.519
	≥ 100 and < 200	19	438/431	−4.86 (−10.07, 0.35)	0.068	59.6%	0.000	
	≥ 200 and < 300	19	430/425	−6.10 (−12.29, 0.10)	0.054	82.0%	0.000	
	≥ 300	7	141/142	1.09 (−6.82, 9.01)	0.787	0.0%	0.762	

Duration (weeks)	≥ 12	36	815/806	−5.10 (−9.55, −0.64)	0.025	76.7%	0.000	0.130
	< 12	12	248/246	−1.09 (−3.75, 1.56)	0.420	0.0%	0.775	

Intervention	CoQ10	30	717/709	−5.74 (−9.99, −1.50)	0.008	73.2%	0.000	0.380
	CoQ10+statin	8	139/140	−1.62 (−15.68, 12.45)	0.822	65.9%	0.005	
	CoQ10+fenofibrate	3	54/51	5.16 (−7.48, 17.80)	0.424	4.1%	0.353	
	CoQ10+another adjunct	6	132/131	−7.34 (−16.14, 1.47)	0.491	57.5%	0.038	
	CoQ10+exercise	1	21/21	9.00 (−16.60, 34.60)	0.491	—	—	

Baseline BMI (kg/m^2^)	≥ 18.5 and < 25	6	118/114	−1.04 (−3.69, 1.61)	0.440	4.7%	0.387	0.062
	≥ 25 and < 30	25	648/644	−8.57 (−13.48, −3.67)	0.001	74.2%	0.000	
	≥ 30	6	116/116	−2.57 (−12.98, 7.84)	0.628	17.8%	0.298	
	NA	11	181/178	1.03 (−12.10, 14.16)	0.878	76.0%	0.000	

Age (years)	< 45	7	154/155	−4.86 (−12.02, 2.30)	0.183	71.5%	0.002	0.861
	≥ 45 and < 60	25	565/553	−3.36 (−7.52, 0.80)	0.113	53.5%	0.001	
	≥ 60	10	225/225	−5.14 (−13.62, 3.33)	0.234	56.0%	0.015	
	NA	6	119/119	−12.85 (−36.44, 10.74)	0.286	87.2%	0.000	

Baseline TC (mg/dL)	< 200	22	514/513	−6.20 (−10.65, −1.75)	0.006	57.6%	0.000	0.368
	≥ 200	26	549/539	−2.85 (‐−8.62, 2.91)	0.332	77.6%	0.000	

**HDL-C (mg/dL)**								
Overall		48	1070/1058	1.07 (0.22, 1.92)	0.013	80.7%	0.000	
Disease	Dyslipidemia and diabetes	5	90/84	1.67 (−1.01, 4.36)	0.222	37.8%	0.169	0.799
	Diabetes	14	318/320	0.60 (−1.41, 2.60)	0.560	45.5%	0.033	
	PCOS	4	123/123	0.72 (−0.33, 1.78)	0.179	93.5%	0.000	
	CAD	5	125/121	1.23 (−0.15, 2.62)	0.080	0.0%	0.537	
	Dyslipidemia	13	275/270	0.88 (−0.38, 2.14)	0.173	0.0%	0.556	
	NAFLD	2	40/42	−1.20 (−5.05, 2.64)	0.540	0.0%	0.703	
	Dyslipidemia and CAD	3	55/54	4.25 (−4.67, 13.16)	0.350	92.7%	0.000	
	Metabolic syndrome	2	44/44	−4.06 (−12.20, 4.07)	0.328	87.8%	0.004	

Region	Asia	26	667/659	1.29 (0.21, 2.36)	0.019	89.2%	0.000	0.854
	Europe	13	228/225	0.53 (−0.91, 1.98)	0.471	0.0%	0.803	
	Oceania	7	131/129	0.80 (−0.98, 2.58)	0.380	0.0%	0.677	
	North America	2	44/45	1.57 (−3.22, 6.35)	0.521	0.0%	0.424	

Dosage (mg/day)	< 100	3	54/54	−2.76 (−8.32, 2.79)	0.330	80.9%	0.005	0.512
	≥ 100 and < 200	19	445/437	1.27 (0.09, 2.45)	0.035	16.7%	0.250	
	≥ 200 and < 300	19	430/425	1.48 (0.28, 2.68)	0.016	91.0%	0.000	
	≥ 300	7	141/142	0.76 (−1.61, 3.12)	0.531	0.0%	0.932	

Duration (weeks)	≥ 12	36	817/808	0.88 (−0.35, 2.12)	0.161	82.8%	0.000	0.336
	< 12	12	253/250	1.52 (1.14, 1.90)	0.000	0.0%	0.681	

Intervention	CoQ10	32	755/747	1.57 (0.41, 2.72)	0.008	83.8%	0.000	0.228
	CoQ10+statin	7	124/125	1.64 (−0.46, 3.73)	0.126	0.0%	0.735	
	CoQ10+fenofibrate	2	37/35	−0.74 (−2.85, 1.37)	0.493	87.6%	0.000	
	CoQ10+another adjunct	6	133/130	−1.31 (−5.46, 2.83)	0.535	86.3%	0.000	
	CoQ10+exercise	1	21/21	3.00 (−6.75, 12.75)	0.546	—	—	

Baseline BMI (kg/m^2^)	≥ 18.5 and < 25	7	140/135	0.68 (−1.19, 2.54)	0.476	15.9%	0.308	0.916
	≥ 25 and < 30	25	648/644	1.13 (−0.00, 2.26)	0.050	89.1%	0.000	
	≥ 30	6	116/116	1.63 (−0.34, 3.60)	0.105	0.0%	0.550	
	NA	10	166/163	0.91 (−1.05, 2.86)	0.364	25.1%	0.213	

Age (years)	< 45	8	171/172	0.02 (−1.31, 1.34)	0.980	82.1%	0.000	0.621
	≥ 45 and < 60	26	595/582	0.89 (−0.10, 1.87)	0.079	29.2%	0.083	
	≥ 60	9	200/200	2.67 (−2.13, 7.48)	0.276	84.8%	0.000	
	NA	5	104/104	0.71 (−2.00, 3.43)	0.608	58.3%	0.048	

Baseline HDL‐C (mg/dL)	> 50	14	295/292	1.51 (1.12, 1.90)	0.000	0.0%	0.497	0.473
	≤ 50	34	775/766	1.05 (−0.13, 2.24)	0.082	83.7%	0.000	

**LDL-C (mg/dL)**								
Overall		50	1108/1097	−3.98 (−7.00, −0.97)	0.010	78.1%	0.000	
Disease	Dyslipidemia and diabetes	5	90/84	−4.36 (−32.36, 23.64)	0.760	88.8%	0.000	0.134
	Diabetes	14	329/332	−2.61 (−7.92, 2.70)	0.335	76.7%	0.000	
	PCOS	4	123/123	−13.87 (−22.68, −5.06)	0.002	64.1%	0.039	
	CAD	4	95/92	−0.90 (−3.25, 1.46)	0.456	0.0%	0.843	
	Dyslipidemia	16	332/325	−5.52 (−12.48, 1.45)	0.121	57.9%	0.002	
	NAFLD	2	40/42	2.79 (−8.72, 14.30)	0.635	0.0%	0.827	
	Dyslipidemia and CAD	3	55/55	−2.38 (−11.83, 7.07)	0.621	36.4%	0.208	
	Metabolic syndrome	2	44/44	6.38 (−6.75, 19.52)	0.341	91.9%	0.000	

Region	Asia	25	637/631	−6.72 (−10.75, −2.69)	0.001	86.2%	0.000	0.024
	Europe	13	241/239	−3.01 (−10.55, 4.53)	0.434	69.1%	0.000	
	Oceania	9	166/164	4.02 (−2.47, 10.52)	0.225	0.0%	0.784	
	North America	3	64/63	4.04 (−6.67, 14.75)	0.459	0.0%	0.475	

Dosage (mg/day)	< 100	3	54/54	1.36 (−11.37, 14.09)	0.834	88.9%	0.000	0.314
	≥ 100 and < 200	20	452/445	−7.97 (−13.88, −2.06)	0.008	82.6%	0.000	
	≥ 200 and < 300	19	441/438	−3.32 (−7.87, 1.23)	0.153	76.6%	0.000	
	≥ 300	8	161/160	−0.54 (−7.02, 5.94)	0.870	0.0%	0.669	

Duration (weeks)	≥ 12	36	828/821	−4.01 (−7.82, −0.19)	0.039	83.2%	0.000	0.680
	< 12	14	280/276	−2.87 (−7.00, 0.92)	0.138	11.9%	0.323	

Intervention	CoQ10	33	774/767	−4.62 (−8.05, −1.20)	0.008	78.4%	0.000	0.345
	CoQ10+statin	8	144/143	−3.79 (−11.52, 3.93)	0.336	20.5%	0.267	
	CoQ10+fenofibrate	2	37/35	11.60 (−4.37, 27.58)	0.155	0.0%	1.000	
	CoQ10+another adjunct	6	132/131	−4.82 (−17.90, 8.25)	0.470	91.6%	0.000	
	CoQ10+exercise	1	21/21	5.00 (−16.15, 26.15)	0.643	—	—	

Baseline BMI (kg/m^2^)	≥ 18.5 and < 25	8	147/143	0.06 (−1.85, 1.96)	0.953	0.0%	0.682	0.064
	≥ 25 and < 30	25	659/657	−6.58 (−11.05, −2.11)	0.004	83.5%	0.000	
	≥ 30	6	116/116	−0.44 (−4.98, 4.11)	0.851	0.0%	0.528	
	NA	11	186/181	−2.82 (−16.77, 11.14)	0.692	84.5%	0.000	

Age (years)	< 45	9	183/184	−1.15 (−8.43, 6.13)	0.756	87.6%	0.000	0.085
	≥ 45 and < 60	26	585/571	−5.22 (−9.35, −1.10)	0.013	70.9%	0.000	
	≥ 60	11	246/248	1.60 (−3.59, 6.79)	0.547	40.6%	0.078	
	NA	4	94/94	−22.82 (−48.33, 2.70)	0.080	88.8%	0.000	

Baseline LDL‐C (mg/dL)	< 130	36	816/808	−3.54 (−6.93, −0.14)	0.041	76.3%	0.000	0.777
	≥ 130	14	292/289	−4.65 (−11.58, 2.28)	0.189	78.8%	0.000	

**HbA1c (%)**								
Overall		31	752/751	−0.22 (−0.37, −0.06)	0.006	67.2%	0.000	
Disease	Diabetes and IGT	22	557/562	−0.18 (−0.37, 0.01)	0.070	70.8%	0.000	0.089
	Dyslipidemia and diabetes	5	90/84	−0.43 (−0.84, −0.02)	0.038	0.0%	0.733	
	PCOS	1	50/50	−0.47 (−0.66, −0.28)	0.000	—	—	
	CAD	1	28/28	−0.19 (−0.59, 0.21)	0.353	—	—	
	Dyslipidemia	2	27/27	−0.09 (−0.33, 0.15)	0.467	0.0%	0.987	

Region	Asia	17	503/504	−0.39 (−0.65, −0.13)	0.003	76.3%	0.000	0.047
	Europe	5	73/72	−0.02 (−0.12, 0.09)	0.753	0.0%	0.979	
	Oceania	7	132/130	−0.03 (−0.20, 0.13)	0.690	0.0%	0.670	
	North America	2	44/45	0.51 (−0.56, 1.59)	0.349	38.1%	0.204	

Dosage (mg/day)	≥ 100 and < 200	10	248/246	−0.62 (−1.07, −0.16)	0.008	81.4%	0.000	0.102
	≥ 200 and < 300	16	405/405	−0.13 (−0.32, 0.06)	0.173	59.8%	0.001	
	≥ 300	5	99/100	−0.08 (−0.284, 0.13)	0.463	1.1%	0.400	

Duration (weeks)	≥ 12	25	618/617	−0.14 (−0.29, 0.02)	0.077	70.2%	0.000	0.879
	< 12	6	134/134	−0.53 (−1.05, −0.01)	0.046	81.4%	0.000	

Intervention	CoQ10	27	687/689	−0.21 (−0.38, −0.04)	0.017	73.1%	0.000	0.878
	CoQ10+another adjunct	4	65/62	−0.24 (−0.56, 0.08)	0.147	25.0%	0.261	

Baseline BMI (kg/m^2^)	≥ 18.5 and < 25	2	34/34	0.00 (−0.12, 0.12)	1.000	—	—	0.000
	≥ 25 and < 30	19	506/513	−0.11 (−0.28, 0.07)	0.229	58.8%	0.001	
	≥ 30	4	114/110	−0.14 (−0.60, 0.32)	0.560	18.6%	0.298	
	NA	6	123/119	−0.84 (−1.13, −0.56)	0.000	11.6%	0.341	

Age (years)	< 45	2	34/34	0.00 (−0.12, 0.12)	1.000	—	—	0.001
	≥ 45 and < 60	19	499/500	−0.28 (−0.54, −0.02)	0.034	71.8%	0.000	
	≥ 60	7	138/138	−0.02 (−0.17, 0.14)	0.834	0.0%	0.958	
	NA	3	81/79	−0.54 (−0.79, −0.29)	0.000	6.9%	0.342	

**Fasting glucose (mg/dL)**								
Overall		39	978/975	−10.07 (−14.75, −5.39)	0.000	87.6%	0.000	
Disease	Diabetes and IGT	21	534/539	−14.10 (−20.86, −7.33)	0.000	60.2%	0.000	0.001
	Dyslipidemia and diabetes	3	52/49	−13.33 (−40.53, 13.87)	0.337	38.4%	0.197	
	PCOS	3	93/93	−4.07 (−7.71, −0.44)	0.028	0.0%	0.609	
	CAD	3	83/79	−20.83 (−34.62, −7.04)	0.003	89.7%	0.000	
	NAFLD	2	39/40	−21.03 (−71.00, 28.94)	0.409	88.5%	0.003	
	Dyslipidemia	5	133/131	−1.18 (−5.43, 3.06)	0.585	34.0%	0.194	
	Metabolic syndrome	2	44/44	1.66 (−2.92, 6.23)	0.478	0.0%	0.969	

Region	Asia	27	755/751	−12.03 (−17.63, −6.44)	0.000	89.5%	0.000	0.018
	Europe	6	108/107	−1.55 (−6.37, 3.27)	0.528	28.6%	0.221	
	Oceania	4	71/72	−6.70 (−18.43, 5.02)	0.263	0.0%	0.705	
	North America	2	44/45	−27.37 (−54.57, −0.17)	0.049	0.0%	0.647	

Dosage (mg/day)	< 100	2	44/44	1.66 (−2.92, 6.23)	0.478	0.0%	0.969	0.000
	≥ 100 and < 200	15	393/387	−20.96 (−28.56, −13.36)	0.000	87.5%	0.000	
	≥ 200 and < 300	17	442/444	−3.08 (−5.83, −0.34)	0.028	0.0%	0.955	
	≥ 300	5	99/100	−2.90 (−9.61, 3.80)	0.396	36.8%	0.176	

Duration (weeks)	≥ 12	27	687/687	−6.10 (−10.18, −2.02)	0.003	43.1%	0.010	0.185
	< 12	12	291/288	−12.10 (−19.96, −4.23)	0.003	93.9%	0.000	

Intervention	CoQ10	32	835/833	−12.25 (−17.42, −7.08)	0.000	87.8%	0.000	0.000
	CoQ10+another adjunct	7	143/142	−0.22 (−4.01, 3.58)	0.911	0.0%	0.616	

Baseline BMI (kg/m^2^)	≥ 18.5 and < 25	4	89/85	−19.13 (−29.99, −8.28)	0.001	87.7%	0.000	0.004
	≥ 25 and < 30	25	674/676	−3.66 (−6.68, −0.63)	0.018	44.3%	0.010	
	≥ 30	4	92/95	−7.12 (−22.02, 7.79)	0.349	56.1%	0.077	
	NA	6	123/119	−24.82 (−40.79, −8.85)	0.002	40.9%	0.132	

Age (years)	< 45	7	141/142	−1.98 (−5.29, 1.34)	0.242	19.6%	0.280	0.000
	≥ 45 and < 60	21	587/585	−16.27 (−22.69, −9.85)	0.000	84.0%	0.000	
	≥ 60	7	150/150	1.24 (−2.87, 5.34)	0.555	0.0%	0.680	
	NA	4	100/98	−23.50 (−53.32, 6.32)	0.122	72.6%	0.012	

**FINS (*μ*IU/mL)**								
Overall		17	453/451	−2.94 (−4.63, −1.25)	0.001	92.5%	0.000	
Disease	Diabetes and IGT	8	244/245	−2.31 (−3.42, −1.21)	0.000	50.6%	0.048	0.000
	NAFLD	1	20/21	−0.24 (−0.52, 0.04)	0.095	—	—	
	Dyslipidemia and diabetes	2	38/36	1.65 (−2.42, 5.72)	0.426	0.0%	0.720	
	PCOS	2	43/43	−2.17 (−4.85, 0.52)	0.113	0.0%	0.781	
	Dyslipidemia	3	78/77	−1.91 (−3.57, −0.24)	0.025	0.0%	0.563	
	CAD	1	30/29	−23.49 (−27.13, −19.85)	0.000	—	—	

Region	Asia	13	388/388	−3.70 (−5.67, −1.73)	0.000	94.3%	0.000	0.038
	Europe	2	27/27	−1.09 (−3.36, 1.19)	0.348	0.0%	0.798	
	Oceania	2	38/36	1.65 (−2.42, 5.72)	0.426	0.0%	0.720	

Dosage (mg/day)	≥ 100 and < 200	8	248/246	−4.69 (−8.03, −1.34)	0.006	95.9%	0.000	0.211
	≥ 200 and < 300	7	178/178	−1.93 (−3.45, −0.42)	0.012	47.7%	0.075	
	≥ 300	2	27/27	−1.09 (−3.36, 1.19)	0.348	0.0%	0.798	

Duration (weeks)	≥ 12	10	294/292	−2.15 (−3.21, −1.09)	0.000	51.0%	0.031	0.383
	< 12	7	159/159	−4.58 (−9.91, 0.76)	0.093	96.2%	0.000	

Intervention	CoQ10	14	403/402	−3.40 (−5.30, −1.50)	0.000	93.9%	0.000	0.084
	CoQ10+another adjunct	3	50/49	−0.82 (−3.04, 1.41)	0.471	0.5%	0.366	

Baseline BMI (kg/m^2^)	≥ 18.5 and < 25	1	30/29	−23.49 (−27.13, −19.85)	0.000	—	—	0.000
	≥ 25 and < 30	12	331/332	−1.72 (−2.87, −0.56)	0.004	78.8%	0.000	
	≥ 30	1	34/34	−1.58 (−3.60, 0.44)	0.126	—	—	
	NA	3	58/56	−1.40 (−7.26, 4.47)	0.640	75.5%	0.017	

Age (years)	< 45	3	63/64	−0.30 (−0.76, 0.16)	0.207	1.9%	0.361	0.032
	≥ 45 and < 60	12	348/347	−3.57 (−6.04, −1.10)	0.005	92.4%	0.000	
	≥ 60	2	42/40	−1.36 (−4.38, 1.66)	0.376	0.0%	0.938	

**HOMA−IR**								
Overall		11	324/325	−0.82 (−1.36, −0.28)	0.003	90.0%	0.000	
Disease	Diabetes and IGT	7	210/211	−1.17 (−1.47, −0.86)	0.000	8.1%	0.367	0.000
	NAFLD	1	20/21	−0.05 (−0.12, 0.02)	0.136	—	—	
	PCOS	2	43/43	−0.58 (−1.42, 0.27)	0.181	56.9%	0.128	
	Dyslipidemia	1	51/50	−0.74 (−1.35, −0.51)	0.035	—	—	

Dosage (mg/day)	≥ 100 and < 200	6	184/183	−0.84 (−1.55, −0.13)	0.021	71.4%	0.004	0.964
	≥ 200 and < 300	5	140/142	−0.82 (−1.33, −0.31)	0.002	63.3%	0.028	

Duration (weeks)	≥ 12	7	222/222	−1.07 (−1.42, −0.72)	0.000	25.8%	0.232	0.031
	< 12	4	102/103	−0.36 (−0.91, 0.19)	0.204	60.7%	0.054	

Intervention	CoQ10	10	303/303	−0.80 (−1.38, −0.22)	0.007	90.4%	0.000	0.726
	CoQ10+another adjunct	1	21/22	−0.96 (−1.63, −0.29)	0.005	—	—	

Baseline BMI (kg/m^2^)	≥ 25 and < 30	10	324/325	−0.79 (−1.34, −0.24)	0.005	90.8%	0.000	0.615
	NA	1	20/20	−1.38 (−3.60, 0.84)	0.222	—	—	

Age (years)	< 45	3	63/64	−0.33 (−0.92, 0.26)	0.276	71.5%	0.030	0.095
	≥ 45 and < 60	7	237/238	−1.08 (−1.42, −0.74)	0.000	24.1%	0.245	
	≥ 60	1	24/23	−0.53 (−3.30, 2.24)	0.707	—	—	

**CRP (mg/L)**								
Overall		15	394/382	−0.44 (−0.79, −0.09)	0.013	90.9%	0.000	
Disease	CAD	4	79/71	−0.06 (−0.15, 0.03)	0.169	1.7%	0.384	0.181
	Diabetes	4	117/115	−1.08 (−2.63, 0.46)	0.169	94.1%	0.000	
	NAFLD	1	20/21	−5.05 (−10.04, −0.06)	0.047	—	—	
	Dyslipidemia	4	128/127	−0.20 (−0.74, 0.33)	0.459	57.0%	0.073	
	PCOS	2	50/48	−0.24 (−0.67, 0.20)	0.288	5.1%	0.305	

Region	Asia	13	341/330	−0.53 (−0.95, −0.11)	0.014	91.2%	0.000	0.177
	Europe	2	53/52	0.00 (−0.65, 0.65)	0.992	53.0%	0.145	

Dosage (mg/day)	< 100	1	14/12	−0.05 (−0.15, 0.05)	0.348	—	—	0.328
	≥ 100 and < 200	7	202/200	−0.97 (−1.94, −0.00)	0.049	94.5%	0.000	
	≥ 200 and < 300	4	109/106	−0.08 (−0.46, 0.31)	0.694	17.6%	0.303	
	≥ 300	3	69/64	−0.08 (−0.77, 0.61)	0.823	66.2%	0.052	

Duration (weeks)	≥ 12	12	326/316	−0.60 (−1.05, −0.16)	0.008	91.9%	0.000	0.001
	< 12	3	68/66	0.18 (0.02, 0.34)	0.032	0.0%	0.457	

Intervention	CoQ10	13	335/322	−0.43 (−0.80, −0.06)	0.023	92.2%	0.000	0.806
	CoQ10+another adjunct	2	59/60	−0.54 (−1.36, 0.28)	0.193	0.0%	0.965	

Baseline BMI (kg/m^2^)	≥ 18.5 and < 25	1	24/25	−0.57 (−2.05, 0.91)	0.451	—	—	0.072
	≥ 25 and < 30	13	350/337	−0.51 (−0.89, −0.13)	0.009	93.8%	0.000	
	NA	1	20/20	0.22 (−0.28, 0.72)	0.393	—	—	

Age (years)	< 45	2	50/48	−0.24 (−0.67, 0.20)	0.288	5.1%	0.305	0.026
	≥ 45 and < 60	3	118/115	−1.28 (−2.42, −0.15)	0.027	85.0%	0.001	
	≥ 60	9	206/198	−0.02 (−0.17, 0.14)	0.851	45.8%	0.064	
	NA	1	20/21	−5.05 (−10.04, −0.06)	0.047	—	—	

**IL-6**								
Overall		11	239/228	−0.42 (−0.79, −0.05)	0.027	74.0%	0.000	
Disease	Diabetes	1	17/16	0.65 (−0.05, 1.35)	0.069	—	—	0.000
	NAFLD	1	20/21	0.19 (−0.43, 0.80)	0.548	—	—	
	Dyslipidemia	3	73/71	−0.54 (−0.88, −0.20)	0.002	2.9%	0.357	
	CAD	3	51/43	−0.41 (−0.90, 0.08)	0.099	26.9%	0.255	
	PCOS	1	22/21	−0.03 (−0.62, 0.57)	0.933	—	—	
	Diabetes and CAD	1	30/30	−1.51 (−2.09, −0.94)	0.000	—	—	
	Dyslipidemia and CAD	1	26/26	−1.10 (−1.69, −0.52)	0.000	—	—	

Region	Asia	9	186/176	−0.42 (−0.89, 0.04)	0.076	78.3%	0.000	0.954
	Europe	2	53/52	−0.44 (−0.94, 0.06)	0.085	35.7%	0.212	

Dosage (mg/day)	< 100	1	14/12	−0.04 (−0.81, 0.73)	0.918	—	—	0.828
	≥ 100 and < 200	3	64/63	−0.53 (−1.62, 0.56)	0.341	88.2%	0.000	
	≥ 200 and < 300	5	120/117	−0.39 (−0.95, 0.17)	0.168	77.3%	0.001	
	≥ 300	2	41/36	−0.48 (−1.17, 0.20)	0.166	55.0%	0.136	

Duration (weeks)	≥ 12	8	169/160	−0.37 (−0.77, 0.04)	0.076	68.9%	0.002	0.716
	< 12	3	70/68	−0.56 (−1.54, 0.41)	0.257	86.8%	0.001	

Intervention	CoQ10	8	167/158	−0.48 (−0.93, −0.03)	0.038	74.3%	0.000	0.665
	CoQ10+another adjunct	3	72/70	−0.27 (−1.08, 0.54)	0.512	81.3%	0.005	

Baseline BMI (kg/m^2^)	≥ 25 and < 30	9	202/193	−0.50 (−0.89, −0.12)	0.011	70.9%	0.001	0.007
	≥ 30	1	20/19	−0.74 (−1.40, −0.09)	0.025	—	—	
	NA	1	17/16	0.65 (−0.05, 1.35)	0.069	74.0%	0.000	

Age (years)	< 45	1	22/21	−0.03 (−0.62, 0.57)	0.933	—	—	0.002
	≥ 45 and < 60	1	20/19	−0.74 (−1.40, −0.09)	0.02	—	—	
	≥ 60	7	160/151	−0.68 (−1.07, −0.28)	0.001	64.1%	0.010	
	NA	2	37/37	0.39 (−0.07, 0.85)	0.099	0.0%	0.330	

*Note:* Values are presented as WMD or SMD with 95% CI. WMD was applied for the estimation of TG, TC, HDL‐C, LDL‐C, HbA1c, fasting glucose, FINS, HOMA‐IR, and CRP, and SMD was applied for IL‐6. *p*‐effect refers to the overall effect within each subgroup. Heterogeneity was assessed using Cochrane Q test (*p*‐heterogeneity) and quantified with I^2^. *p*‐between subgroups was obtained from the test for subgroup differences.

Abbreviations: CAD, coronary artery disease; CI, confidence interval; CoQ10, coenzyme Q10; CRP, C‐reactive protein; FINS, fasting insulin; HbA1c, hemoglobin A1C; HDL‐C, high‐density lipoprotein cholesterol; HOMA‐IR, homeostasis model assessment–insulin resistance; IL‐6, interleukin‐6; LDL‐C, low‐density lipoprotein cholesterol; NAFLD, nonalcoholic fatty liver disease; PCOS, polycystic ovary syndrome; SMD, standardized mean difference; TC, total cholesterol; TG, triglyceride; WMD, weighted mean difference.

#### 3.3.2. TC

Forty‐eight trial arms (1063 participants in the CoQ10 groups and 1052 in the control groups) showed that CoQ10 supplementation significantly reduced serum TC (WMD = −4.86 mg/dL; 95% CI: −8.41, −1.30) (Figure 2B). Heterogeneity was substantial (*I* ^2^ = 71.7*%*; *p* < 0.001). Subgroup analyses suggested that heterogeneity was partly explained by disease category (*p* between subgroups = 0.005). Significant TC reductions were observed in trials enrolling individuals with PCOS (WMD = −14.53 mg/dL; 95% CI: −24.12, −4.93) and those with dyslipidemia plus CAD (WMD = −14.92 mg/dL; 95% CI: −22.32, −7.53). Trials conducted in Asia also showed a significant reduction in TC. Similarly, significant reductions were observed in subgroups with duration ≥ 12 weeks, intervening by CoQ10 alone, baseline BMI of ≥ 25 and < 30, or baseline TC of < 200 mg/dL (Table 2).

#### 3.3.3. HDL‐C

Across 48 trial arms (1070 participants receiving CoQ10 and 1058 controls), CoQ10 supplementation was associated with a significant increase in HDL‐C (WMD = 1.07 mg/dL; 95% CI: 0.22, 1.92) (Figure 2C). Significant heterogeneity was estimated (*I* ^2^ = 80.7*%*; *p* < 0.001), and subgroup analyses did not identify a clear source of heterogeneity across disease, region, dosage, duration, intervention, baseline BMI, age, and baseline HDL‐C. In subgroup analyses, HDL‐C increases were significant in trials conducted in Asia, trials with dosages of ≥ 100 and < 200 or ≥ 200 and < 300 mg/day, trials with duration < 12 weeks, trials evaluating CoQ10 alone, and trials with baseline HDL‐C of > 50 mg/dL (Table 2).

#### 3.3.4. LDL‐C

Serum LDL‐C was reported in 50 trial arms (1108 participants in the CoQ10 groups and 1097 in the control groups). Pooled results revealed that the CoQ10 supplement significantly reduced LDL‐C concentrations (WMD = −3.98 mg/dL; 95% CI: −7.00, −0.97; *I* ^2^ = 78.1*%*; *p* < 0.001) (Figure 2D). Subgroup analyses indicated that heterogeneity was partly explained by region (*p* between subgroups = 0.024). Significant LDL‐C reductions were observed in trials enrolling participants with PCOS, conducted in Asia, using doses at ≥ 100 and < 200 mg/day, lasting ≥ 12 weeks, evaluating CoQ10 monotherapy, with baseline BMI ≥ 25 and < 30, with mean age ≥ 45 and < 60, and with baseline TC < 130 mg/dL (Table 2).

#### 3.3.5. Adiponectin and Leptin

Four RCTs (103 participants in the CoQ10 groups and 100 in the control groups) reported adiponectin, and two RCTs (54 participants in the CoQ10 groups and 55 in the control groups) reported leptin. The pooled estimates showed no significant effect of CoQ10 on adiponectin (WMD = 1.37 *μ*g/mL; 95% CI: −0.35, 3.27; *I* ^2^ = 65.9*%*; *p* = 0.032) (Figure 2E) or leptin (SMD = −0.32; 95% CI: −0.70, 0.06; *I* ^2^ = 0.0*%*; *p* = 0.872) (Figure 2F), as both CIs crossed the null. Owing to the limited number of studies, no subgroup analyses were performed.

### 3.4. Effects of CoQ10 Supplementation on Glucose Metabolism

#### 3.4.1. HbA1c

**Figure 3:**
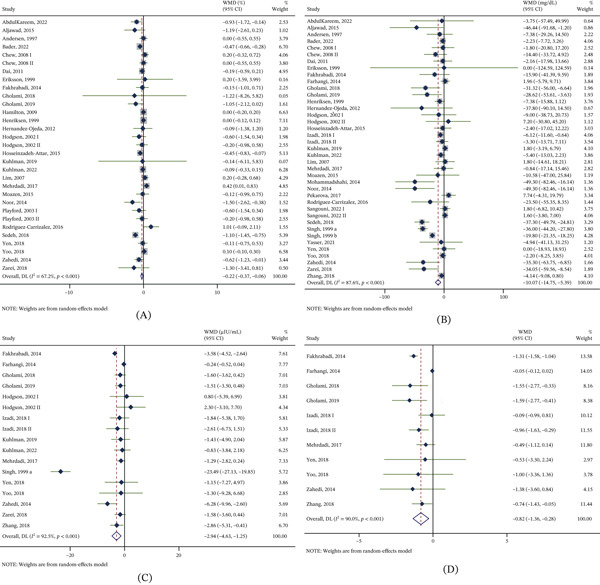
Forest plots of effects of coenzyme Q10 supplementation on glucose metabolism. Data from randomized controlled trials were analyzed using a random‐effects model and are presented as WMDs with 95% CIs for (A) hemoglobin A1C (*n* = 1503), (B) fasting plasma glucose (*n* = 1953), (C) fasting insulin (*n* = 904), and (D) homeostasis model assessment–insulin resistance (*n* = 649). Each square represents an individual study with its corresponding weight, and horizontal lines denote 95% CIs. The diamond indicates the pooled effect size. Statistical significance was considered when the 95% CI did not cross the vertical line of no effect. Abbreviations: WMD, weighted mean difference; CI, confidence interval.

Based on 31 trial arms (752 participants in the CoQ10 groups and 751 in the control groups), CoQ10 supplementation was associated with a significant reduction in HbA1c (WMD = −0.22*%*; 95% CI: −0.37, −0.06; *I* ^2^ = 67.2*%*; *p* < 0.001) (Figure 3A). Substantial heterogeneity was observed, with region, baseline BMI, and age suggested as potential contributors. Subgroup analyses showed significant HbA1c reductions in participants with dyslipidemic diabetes or PCOS, and in trials conducted in Asia, enrolling participants aged ≥ 45 and < 60 years, using CoQ10 monotherapy, administering ≥ 100 and < 200 mg/day, or lasting < 12 weeks (Table 2).

#### 3.4.2. Fasting Glucose

Thirty‐nine studies (978 participants in the CoQ10 groups and 975 in the control groups) assessed fasting glucose. Pooled analysis showed that CoQ10 supplementation significantly reduced fasting glucose, with substantial heterogeneity (WMD = −10.07 mg/dL; 95% CI: −14.75, −5.39; *I* ^2^ = 87.6*%*; *p* < 0.001) (Figure 3B). Subgroup analyses suggested that heterogeneity was explained by disease, region, dose, intervention type, baseline BMI, and age (all *p* between subgroups < 0.05, Table 2). Significant reductions were observed among participants with dysglycemia, CAD, or PCOS; those with baseline BMI ≥ 18.5 and < 25 or ≥ 25 and < 30 kg/m^2^; and those aged ≥ 45 and < 60 years. Effects were also significant in trials conducted in Asia or North America, in trials using doses of ≥ 100 and < 200 or ≥ 200 and < 300 mg/day, and in trials using CoQ10 monotherapy (Table 2). When stratified by duration, fasting glucose reductions remained significant across all duration subgroups.

#### 3.4.3. FINS

Meta‐analysis with 17 arms (453 participants in the CoQ10 groups and 451 in the control groups) showed that CoQ10 supplementation significantly reduced FINS (WMD = −2.94 *μ*IU/mL; 95% CI: −4.63, −1.25; *I* ^2^ = 92.5*%*; *p* < 0.001) (Figure 3C). Subgroup analysis indicated that disease, region, BMI, and age may contribute to between‐study heterogeneity (all *p* between subgroups < 0.05, Table 2). Significant FINS reductions were observed in studies including participants with dyslipidemia, CAD, diabetes, or IGT, and in those aged ≥ 45 and < 60 or those with BMI of ≥ 18.5 and < 25 or ≥ 25 and < 30. Reductions were also significant in trials conducted in Asia, using doses of ≥ 100 and < 200 or ≥ 200 and < 300 mg/day, and evaluating CoQ10 monotherapy (Table 2).

#### 3.4.4. HOMA‐IR

A meta‐analysis of 11 trial arms (324 participants in the CoQ10 groups and 325 in the control groups) showed that CoQ10 supplementation significantly reduced HOMA‐IR (WMD = −0.82; 95% CI: −1.36, −0.28; *I* ^2^ = 90.0*%*; *p* < 0.001) (Figure 3D). Subgroup analyses suggested that heterogeneity was mainly attributable to disease and duration (both *p* between subgroups < 0.005, Table 2). Significant reductions were observed in participants with dyslipidemia or dysglycemia, and in those aged ≥ 45 and < 60. Besides, CoQ10 was associated with significant improvements among participants with baseline BMI ≥ 25 and < 30 kg/m^2^. Benefits were also evident in trials using doses of 100–200 or 200–300 mg/day, lasting ≥ 12 weeks, and administering CoQ10 alone or combined with an adjunct (e.g., vitamin E) (Table 2).

### 3.5. Effects of CoQ10 Supplementation on Biomarkers of Inflammation

#### 3.5.1. CRP

**Figure 4:**
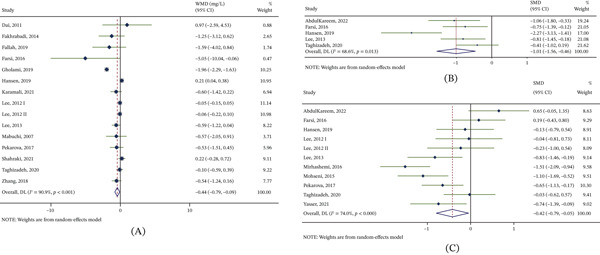
Forest plots of effects of coenzyme Q10 supplementation on biomarkers of inflammation. Data from randomized controlled trials were analyzed using a random‐effects model and are presented as WMD with 95% CIs for (A) C‐reactive protein (*n* = 776), and SMDs with 95% CI were calculated for (B) tumor necrosis factor‐*α* (*n* = 194) and (C) interleukin‐6 (*n* = 467). Each square represents an individual study with its corresponding weight, and horizontal lines denote 95% CIs. The diamond indicates the pooled effect size. Statistical significance was considered when the 95% CI did not cross the vertical line of no effect. Abbreviations: WMD, weighted mean difference; CI, confidence interval; SMD, standardized mean difference.

The effect of the CoQ10 supplement on CRP was evaluated in 15 arms (394 participants in the CoQ10 groups and 382 in the control groups), indicating a significant difference (WMD = −0.44 mg/L; 95% CI: −0.79, −0.09; *I* ^2^ = 90.9*%*; *p* < 0.001) (Figure 4A). Subgroup analyses identified duration (*p* between subgroups = 0.001) and age (*p* between subgroups = 0.026) as sources of heterogeneity, with significant CRP reductions observed in trials enrolling participants aged ≥ 45 and < 60 years. By disease subgroup, CoQ10 was associated with a marked CRP decrease in NAFLD (WMD = −5.05 mg/L; 95% CI: −10.04, −0.06). When stratified by duration, CRP showed significant changes in both subgroups, with a reduction in the ≥ 12‐week subgroup and an increase in the < 12‐week subgroup. However, subgroup findings were based on limited sample sizes (Table 2).

#### 3.5.2. TNF‐*α*

Pooled analysis of five RCTs (100 participants in the CoQ10 groups and 94 in the control groups) showed that CoQ10 supplement significantly reduced TNF‐*α* levels (SMD = −1.01; 95% CI: −1.56, −0.46; *I* ^2^ = 68.6*%*; *p* = 0.013) (Figure 4B). Subgroup analyses were not performed due to the limited number of included studies.

#### 3.5.3. IL‐6

Ten trials (239 participants in the CoQ10 groups and 228 in the control groups) reported IL‐6 values. The pooled analysis indicated that CoQ10 supplementation significantly reduced IL‐6 levels (SMD = −0.42; 95% CI: −0.79, −0.05; *I* ^2^ = 74.0*%*; *p* < 0.001) (Figure 4C). Subgroup analysis revealed that disease, baseline BMI, and age (all *p* between subgroups < 0.05) (Table 2) contributed to the observed heterogeneity. Significant IL‐6 reductions were observed among participants with dyslipidemia, diabetes plus CAD, or dyslipidemia plus CAD; among those aged ≥ 45 and < 60 or ≥ 60 years; and among those with baseline BMI ≥ 25 and < 30 and ≥ 30 kg/m^2^. Besides, trials using CoQ10 as the sole intervention showed a significant association between CoQ10 supplementation and lower IL‐6 levels (Table 2).

### 3.6. Meta‐Regressions

Univariate meta‐regression analyses were performed to evaluate the potential moderating effects of dosage, duration, age, baseline BMI, and baseline levels of the corresponding outcomes. Across all outcomes, no statistically significant associations were identified for the majority of moderators (all *p* > 0.05), with the exception of a trend toward significance for baseline BMI on FINS (*p* = 0.055) (Supporting Files S3–S5). Additionally, the dosage × duration interaction terms were not significant for any outcome (all *p* > 0.05), indicating no evidence of an interaction between dose and duration in modifying treatment effects (Supporting File S3).

### 3.7. Sensitivity Analysis and Publication Bias

Leave‐one‐out sensitivity analyses showed that the pooled results for TC, LDL‐C, HbA1c, fasting glucose, FINS, and HOMA‐IR were robust. However, the pooled effect for TG became nonsignificant after excluding Bader et al. (WMD = −4.74 mg/dL; 95% CI: −9.57, 0.09) [57]. For HDL‐C, the exclusion of Mohseni et al. resulted in a nonsignificant estimate (WMD = 0.58 mg/dL; 95% CI: −0.08, 1.25) [93]. For CRP, the pooled estimate lost statistical significance when omitting Gholami et al. (WMD = −0.09 mg/L; 95% CI: −0.25, 0.07) [63]. For IL‐6, the exclusion of any single study by Lee et al. [86], Mirhashemi et al. [92], Mohseni et al. [93], Yasser et al. [69], or Pekarova et al. [53] resulted in a loss of statistical significance.

Except for TNF‐*α*, adiponectin, and leptin, all other outcome indicators were evaluated for publication bias using funnel plots, Begg′s test, and Egger′s test (Figure S3A–J). Begg′s and Egger′s tests showed no evidence of publication bias for most outcomes. However, potential bias was detected for fasting glucose (Begg′s test, *p* < 0.001) and HOMA‐IR (Egger′s test, *p* = 0.046). Therefore, the trim‐and‐fill method was applied. For fasting glucose, the trim‐and‐fill method did not impute any missing studies, and the adjusted effect remained significant (WMD = −10.07 mg/dL; 95% CI: −14.75, −5.39), consistent with the original results. For HOMA‐IR, the trim‐and‐fill method imputed three missing studies, and the adjusted pooled effect remained significant (WMD = −0.63; 95%CI: −1.01, −0.26), supporting the robustness of the findings (Figure S4A–B).

### 3.8. Risk of Bias and Grading of Evidence

**Figure 5:**
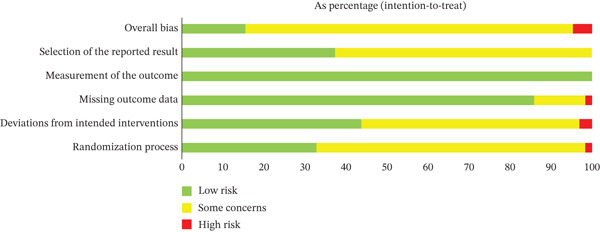
The summary of risk of bias assessment.

RoB 2 assessment indicated that most domains were judged as low risk of bias, particularly the measurement of the outcome and the missing outcome data. However, some concerns were frequently noted for the randomization process, deviations from intended interventions, and selection of the reported result, additionally, a small proportion of studies were rated as high risk. Overall, most studies were rated as having some concerns, followed by low risk of bias, with few at high risk. The summary of the risk of bias assessment is shown in Figure 5, and the individual study risk of bias assessments are provided in Supporting File S8.

Regarding the certainty of evidence (Table 3), TG and TNF‐*α* were rated as moderate certainties owing to the potential risk of bias. The quality of TC, HDL‐C, LDL‐C, HbA1c, FINS, CRP, and IL‐6 was low grade due to degradation factors including risk of bias and inconsistency. The certainty of fasting glucose and HOMA‐IR was downgraded to very low, owing to the terms of risk of bias, inconsistency, and the detected publication bias.

**Table 3:** GRADE evidence profile for effects of CoQ10 supplementation on lipid profiles, glucose metabolism, and biomarkers of inflammation.

Certainty assessment								No of patients		Effect	Certainty
Outcomes	No. of studies	Study design	Risk of bias	Inconsistency	Indirectness	Imprecision	Other considerations	Coenzyme Q10	Control	Absolute (95% CI)	
TG	42	Randomized trials	Serious^a^	Not serious	Not serious	Not serious	None	933	928	MD 5.67 mg/dL lower (10.57 lower to 0.77 lower)	Moderate
TC	48	Randomized trials	Serious^a^	Serious^b^	Not serious	Not serious	None	1063	1052	MD 4.86 mg/dL lower (8.41 lower to 1.30 lower)	Low
HDL‐c	48	Randomized trials	Serious^a^	Serious^b^	Not serious	Not serious	None	1070	1058	MD 1.07 mg/dL higher (0.22 higher to 1.92 higher)	Low
LDL‐c	50	Randomized trials	Serious^a^	Serious^b^	Not serious	Not serious	None	1108	1097	MD 3.98 mg/dL lower (7.00 lower to 0.97 lower)	Low
HbA1c	31	Randomized trials	Serious^a^	Serious^b^	Not serious	Not serious	None	752	751	MD 0.22% lower (0.37 lower to 0.06 lower)	Low
Fasting glucose	39	Randomized trials	Serious^a^	Serious^b^	Not serious	Not serious	Publication bias strongly suspected^c^	978	975	MD 10.07 mg/dL lower (14.75 lower to 5.39 lower)	Very low
FINS	17	Randomized trials	Serious^a^	Serious^b^	Not serious	Not serious	None	453	451	MD 2.94 *μ*IU/mL lower (4.63 lower to 1.25 lower)	Low
HOMA‐IR	11	Randomized trials	Serious^a^	Serious^b^	Not serious	Not serious	Publication bias strongly suspected^d^	324	325	MD 0.82 lower (1.36 lower to 0.28 lower)	Very low
CRP	15	Randomized trials	Serious^a^	Serious^b^	Not serious	Not serious	None	394	382	MD 0.44 mg/L lower (0.79 lower to 0.09 lower)	Low
TNF‐*α*	5	Randomized trials	Serious^a^	Not serious	Not serious	Not serious	None	100	94	SMD 1.01 SD lower (1.56 lower to 0.46 lower)	Moderate
IL‐6	11	Randomized trials	Serious^a^	Serious^b^	Not serious	Not serious	None	239	228	SMD 0.42 SD lower (0.79 lower to 0.05 lower)	Low

Abbreviations: CI, confidence interval; CRP, C‐reactive protein; FINS, fasting insulin; HbA1c, hemoglobin A1C; HDL‐C, high‐density lipoprotein cholesterol; HOMA‐IR, homeostasis model assessment–insulin resistance; IL‐6, interleukin‐6; LDL‐C, low‐density lipoprotein cholesterol; MD, mean difference; SMD, standardized mean difference; TC, total cholesterol; TG, triglyceride; TNF‐*α*, tumor necrosis factor‐*α*.

^a^Some studies did not report randomness or assignment concealment.

^b^The test for heterogeneity is significant (I^2^ > 50%).

^c^Begg′s test shows potential publication bias (*p* < 0.05).

^d^Egger′s test shows potential publication bias (*p* < 0.05).

## 4. Discussion

This meta‐analysis evaluated the effects of CoQ10 on lipid and glycemic metabolism and inflammation, and explored the potential differences in effects among various diseases, physical traits, and CoQ10 regimens. Based on the comprehensive analyses of relevant RCTs, this study might yield new insights into the metabolic benefits of CoQ10.

Overall, CoQ10 supplementation was associated with improved lipid, glycemic, and inflammatory biomarkers in individuals with metabolic abnormalities. For lipid profiles, CoQ10 supplementation significantly decreased TG, TC, and LDL‐C levels, and increased HDL‐C concentrations. Given that elevated TG, TC, and LDL‐C levels are associated with increased cardiovascular disease incidence and mortality, whereas HDL‐C levels are inversely associated with these outcomes [18, 96, 97], the observed modest lipid‐lowering effect might suggest an adjunctive effect on lipid metabolism and a potential contribution to cardiovascular protection, though it did not represent a clinically meaningful therapeutic effect. In addition, significant improvements were observed in glycemic parameters (HbA1c, fasting glucose, FINS, and HOMA‐IR) and inflammatory cytokines (CRP, TNF‐*α*, and IL‐6). By contrast, the effects on adiponectin and leptin were not statistically significant, and the limited number of studies reporting these outcomes precluded firm conclusions, highlighting the need for further research.

We next performed stratified analyses to elucidate the beneficial effects of CoQ10 across different metabolic diseases, supplementation doses and durations, and BMI categories. Disease‐specific subgroup analyses revealed that CoQ10 supplementation did not significantly improve lipid profiles among patients with dyslipidemia or those with abnormal baseline lipid levels, whereas mild improvements in TC, LDL‐C, and HDL‐C levels were observed among participants with normal baseline lipid profiles. Notably, among trials involving dyslipidemic patients, a substantial proportion compared CoQ10‐combined statin therapy versus statin therapy alone (4/13 for TG, 6/15 for TC, 6/16 for LDL‐C, and 5/13 for HDL‐C). Given that statins lower LDL‐C levels by inhibiting cholesterol synthesis, this concomitant therapy may have confounded the adjunctive lipid‐lowering effects of CoQ10 [98]. Consistently, subgroup results revealed significant lipid improvements in studies using CoQ10 alone but not in those using CoQ10‐statin combinations, suggesting that CoQ10 might not confer additional lipid‐reducing benefits when coadministered with statins. Nevertheless, the observed effects of CoQ10 on FINS, HOMA‐IR and IL‐6 levels among dyslipidemic participants implied that CoQ10 may exert compensatory benefits by improving glycometabolic and inflammatory disturbances commonly associated with dyslipidemia [99]. Collectively, these findings improved our understanding of CoQ10′s role in dyslipidemic individuals, suggesting that it might not provide additional lipid‐lowering benefits when coadministered with statins. However, the independent effects of CoQ10 on lipid metabolism, particularly in the absence of statin use, require further investigation in future trials.

Among individuals with glucose metabolism abnormalities, including diabetes and IGT, CoQ10 supplementation significantly improved HbA1c, fasting glucose, FINS, and HOMA‐IR levels. These clinical findings were also supported by in vitro evidence demonstrating that CoQ10 protects pancreatic *β* cells from staurosporine‐induced apoptosis [100].

Subgroup analyses further demonstrated significant benefits of CoQ10 across several interrelated metabolic disorders, including PCOS, CAD, and NAFLD. Specifically, in patients with PCOS—a condition characterized by reproductive dysfunctions and metabolic abnormalities such as hyperandrogenemia, dyslipidemia, IGT, and IR [99, 101]—CoQ10 supplementation significantly improved both lipid and glucose profiles. Among participants with CAD, CoQ10 consumption effectively reduced fasting glucose and insulin levels, supporting its cardiovascular benefits given the established role of glycemic control in mitigating cardiovascular risk [102]. Beyond glucose regulation, CoQ10 has also demonstrated additional cardioprotective actions, including modulation of blood pressure and attenuation of oxidative stress biomarkers [103]. Furthermore, CoQ10 may protect cardiac cells from IR‐induced apoptosis by attenuating apoptotic deoxyribonucleic acid (DNA) and modulating the expression of the B‐cell lymphoma‐2 (Bcl‐2) gene [104]. Additionally, CoQ10 reduced CRP levels in NAFLD, demonstrating its anti‐inflammatory effects. Collectively, these findings highlighted the potential benefits of CoQ10 in alleviating various metabolic disturbances.

The included clinical trials encompassed a wide range of CoQ10 dosages (60–600 mg/day). To explore the optimal intake, we performed subgroup analyses stratified by dosage. Dose brackets were defined based on commonly applied therapeutic ranges in nutritional and clinical contexts, as well as the distribution of doses across the included trials. Our results indicated that CoQ10 supplementation at 100–200 mg/day significantly improved most lipid and glycemic parameters. In contrast, supplementation at 200–300 mg/day showed improvements in only a limited number of these outcomes, and no significant effects were observed for other dosage ranges. Subgroup analysis of fasting glucose further indicated that the beneficial effect was greater at 100–200 mg/day than at 200–300 mg/day. These findings aligned with a previous meta‐analysis reporting a “U” shape dose‐response curve of CoQ10 dosage and outcome indicators of glycemic control, with an optimal dosage of 100–200 mg/day [20]. Pharmacokinetic studies further indicated that although plasma CoQ10 concentrations increase with oral intake, the increment is not proportional at higher doses [105]. More importantly, tissue uptake of CoQ10 remains limited even when plasma concentrations are elevated, suggesting that higher plasma levels do not necessarily translate to increased tissue availability or enhanced clinical benefit [106, 107]. Collectively, these results supported that supplementation of CoQ10 at 100–200 mg/day might represent an effective and well‐established regimen that balances physiological benefits with practicality and safety. Accordingly, a daily intake of 100–200 mg of CoQ10 is recommended for optimal outcomes. Regarding treatment duration, although meta‐regression analyses did not identify a strong duration–effect relationship, most lipid, glycemic, and inflammatory parameters showed significant improvements with interventions lasting at least 12 weeks. In contrast, trials with shorter durations (< 12 weeks) demonstrated benefits in only a limited number of outcomes. Particularly, results indicated that CoQ10 yielded greater improvements in HOMA‐IR and CRP when administered for at least 12 weeks compared with shorter durations. Furthermore, dose–duration interaction analyses suggested that the effects of CoQ10 were independent of the combination of dose and duration. Collectively, these findings suggested that a minimum supplementation period of 12 weeks is recommended to achieve broader metabolic benefits.

In addition to the aforementioned sources of heterogeneity, our estimates identified BMI as a significant contributor. Subgroup analyses indicated that CoQ10 significantly improved TC, LDL‐C, fasting glucose, FINS, HOMA‐IR, CRP, and IL‐6 levels among participants with a BMI of 25–30 kg/m^2^. These findings suggested that individuals with a BMI of 25–30 kg/m^2^ might benefit more from CoQ10 supplementation.

A previous meta‐analysis reported favorable impacts of CoQ10 on lipid profiles and suggested a daily intake of 400–500 mg of CoQ10 [18]. In contrast, based on both lipid and glycemic outcomes, we recommended a daily dose of 100–200 mg. Consistent with the findings of Liang et al. [20], our meta‐analysis confirmed significant improvements in glycemic control following CoQ10 supplementation. However, we identified a larger effect size in terms of WMD compared with Liang et al.′s study, which primarily included healthy participants. The inclusion of nondiseased individuals may have attenuated the overall effect in their analysis. Regarding inflammatory biomarkers, our results revealed significant reductions in serum CRP and IL‐6, in contrast to previously published meta‐analyses [21, 23]. Nevertheless, the estimates for these inflammatory markers remain unstable, which accentuates the need for more well‐designed RCTs.

The main strength of this review lies in its synthetic evaluation of CoQ10 efficacy across multiple interrelated domains, including lipid profiles, glucose metabolism and inflammation, which guaranteed the comprehensiveness of our analysis. Besides, subgroup and meta‐regression analyses provided detailed insights into the different effects of CoQ10 across various metabolic diseases, the optimal dosage and duration, and the physical characteristics of the beneficiary population. These findings collectively offered new insights for clinical recommendations. The data were derived from the most updated RCTs, identified through a thorough search and rigorous screening process to minimize publication biases and other potential biases. Publication bias was further assessed, adjusted using trim‐and‐fill methods, which further guarantees the avoidance of publication biases. In addition to CoQ10, other dietary supplements such as *Nigella sativa*, sesame, and cinnamon have also been shown to improve lipid and glucose metabolism as well as inflammatory status in metabolic disorders, highlighting the growing interest in bioactive dietary ingredients for metabolic regulation [108–110]. Against this broader context, the present findings underscored the distinctive role and clinical significance of CoQ10 supplementation, whose dual role in mitochondrial electron transport and antioxidant defense enables it to modulate both energy metabolism and inflammatory responses, thereby contributing to the management of lipid and glycemic metabolic abnormalities [10–12]. However, there are limitations. First, most outcomes exhibited substantial heterogeneity, and exploratory subgroup analyses and meta‐regression did not fully explain it. Therefore, these pooled estimates should be interpreted cautiously, and the clinical applicability of the findings may be limited. Unexplained variation may stem from methodological differences or unmeasured factors such as CoQ10 formulations or participants′ genetic and dietary backgrounds [105, 111]. Second, the exclusion of non‐English publications may have introduced language bias. Third, multiple exploratory subgroup analyses were conducted without adjustment for multiplicity, and given the relatively small number of studies in certain subgroups, these findings should be interpreted as hypothesis‐generating rather than confirmatory. Furthermore, the predominance of studies from Asia, particularly Iran, may limit the generalizability of the findings to populations with different genetic, dietary, or clinical characteristics. Results concerning adiponectin and leptin should also be interpreted cautiously due to the small number of contributing studies. In addition, based on the GRADE assessment, 7 of the 11 outcomes were rated as low certainty and two as very low certainty. These findings, while indicative of potential benefits, should therefore be regarded as preliminary and warrant confirmation through future high‐quality RCTs.

In conclusion, this meta‐analysis demonstrates that CoQ10 supplementation exerts beneficial effects on lipid metabolism, glycemic control, and inflammatory biomarkers among individuals with metabolic disorders. Compared with previous meta‐analyses that focused on limited outcomes or single metabolic conditions, our study integrated a larger pool of RCTs and provided a more comprehensive evaluation across multiple metabolic parameters. Importantly, our findings suggested that a daily dose of 100–200 mg of CoQ10 and a supplementation duration of at least 12 weeks may yield optimal physiological benefits, particularly among individuals with a BMI of 25–30 kg/m^2^. Notably, results revealed that CoQ10 supplementation did not appear to provide additional lipid‐lowering benefits when administered alongside statins or in individuals with abnormal baseline lipid levels. Nonetheless, substantial heterogeneity across studies and the low certainty of evidence (ranging from very low to moderate) indicate that these findings should be interpreted with caution. Moreover, unconfounded estimates—such as the independent effects of CoQ10 on lipid profiles—remain insufficiently investigated. Therefore, to advance the field, future large‐scale, well‐designed RCTs with standardized formulations are warranted to confirm these results and to inform clinical guidelines.

**Nomenclature**
Bcl‐2: B‐cell lymphoma‐2
CAD: coronary artery disease
CI: confidence interval
CoQ10: coenzyme Q10
CRP: C‐reactive protein
DNA: deoxyribonucleic acid
FINS: fasting insulin
HbA1c: hemoglobin A1C
HDL‐C: high‐density lipoprotein cholesterol
HOMA‐IR: homeostasis model assessment–insulin resistance
IGT: impaired glucose tolerance
IL‐6: interleukin‐6
IR: insulin resistance
LDL‐C: low‐density lipoprotein cholesterol
NAFLD: nonalcoholic fatty liver disease
PCOS: polycystic ovary syndrome
RCT: randomized controlled trial
SD: standard deviation
SMD: standardized mean difference
TC: total cholesterol
TG: triglyceride
TNF‐*α*
: tumor necrosis factor‐*α*
WMD: weighted mean difference

## Author Contributions

M.Z. and Y.S. designed research; Z.Z. and Z.L. conducted research; Y.G., Y.H., R.H., and F.L. analyzed data; Z.Z., Z.L., and Y.S. wrote the paper. Z.Z. and Z.L. contributed equally to this work.

## Funding

This study was supported by the cooperation project of Integrated Chinese and Western Medicine of Refractory Diseases, Hubei Province.

## Disclosure

All authors read and approved the final manuscript.

## Conflicts of Interest

The authors declare no conflicts of interest.

## Supporting Information

Additional supporting information can be found online in the Supporting Information section.

## Supporting information


**Supporting Information 1** Supporting File S1: Changes to the protocol.

**Supporting Information 2** Supporting File S2: Search strategy.

**Supporting Information 3** Supporting File S3: Univariate and interaction meta‐regression analyses to investigate the potential impact of various moderators on pooled estimates.

**Supporting Information 4** Figure S1: Meta‐regressions to investigate the relationship between dosage and estimated net changes in outcome indicators: (A) TG, (B) TC, (C) HDL‐C, (D) LDL‐C, (E) HbA1c, (F) Fasting glucose, (G) FINS, (H) HOMA‐IR, (I) CPR, and (J) IL‐6.

**Supporting Information 5** Figure S2: Meta‐regressions to investigate the relationship between duration and estimated net changes in outcome indicators: (A) TG, (B) TC, (C) HDL‐C, (D) LDL‐C, (E) HbA1c, (F) fasting glucose, (G) FINS, (H) HOMA‐IR, (I) CPR, and (J) IL‐6.

**Supporting Information 6** Figure S3: Funnel plots of (A) TG, (B) TC, (C) HDL‐C, (D) LDL‐C, (E) HbA1c, (F) fasting glucose, (G) FINS, (H) HOMA‐IR, (I) CPR, and (J) IL‐6.

**Supporting Information 7** Figure S4: The trim‐and‐fill method for (A) fasting glucose and (B) HOMA‐IR.

**Supporting Information 8** Supporting File S8: Individual study risk of bias assessments.

## Data Availability

All data generated or analyzed during this study are available in this article/Supporting Information.
