# Supplementary material for: Effects of Coenzyme Q10 on Lipid, Glycemic, and Inflammatory Markers in Metabolic Disorders: A Systematic Review and Meta‐Analysis
Source: J Diabetes Res. 2026 May 26;2026:5587445. doi: 10.1155/jdr/5587445 (PMC13212042; doi:10.1155/jdr/5587445)
Supplement: Supplementary file 1 — Supporting Information 1 Supporting File S1: Changes to the protocol. [file JDR-2026-5587445-s007.docx]

**Supplementary file 1: Changes to the protocol**

1. The population of inclusion criteria was defined as patients with glycolipid metabolic abnormalities, excluding patients with breast cancer, bipolar disorder, chronic kidney disease or other non-metabolic disorders.
2. In addition to the primary outcomes mentioned in the protocol, adiponectin and leptin were included in the analysis as secondary outcomes.
3. Statistical analysis were performed using Stata software (version 16.0) instead of RevManV.5.4.
4. Updating search to January 2026.
