# Supplementary material for: Effects of Coenzyme Q10 on Lipid, Glycemic, and Inflammatory Markers in Metabolic Disorders: A Systematic Review and Meta‐Analysis
Source: J Diabetes Res. 2026 May 26;2026:5587445. doi: 10.1155/jdr/5587445 (PMC13212042; doi:10.1155/jdr/5587445)
Supplement: Supplementary file 2 — Supporting Information 2 Supporting File S2: Search strategy. [file JDR-2026-5587445-s001.docx]

**Supplementary file 2: Search strategy**

**PUBMED**

| Search | Query | Results |
| --- | --- | --- |
| #30 | Search: #3 AND #25 Filters: Clinical Trial, Randomized Controlled Trial, Humans, English | 215 |
| #26 | Search: #3 AND #25 | 2,735 |
| #25 | Search: #10 OR #15 OR #24 | 2,625,378 |
| #24 | Search: #16 OR #17 OR #18 OR #19 OR #20 OR #21 OR #22 OR #23 | 1,379,954 |
| #23 | Search: ((((((("C-Reactive Protein"[Title/Abstract]) OR ("CRP protein"[Title/Abstract])) OR ("C Reactive Protein"[Title/Abstract])) OR ("hsCRP"[Title/Abstract])) OR ("High Sensitivity C-Reactive Protein"[Title/Abstract])) OR ("High Sensitivity C Reactive Protein"[Title/Abstract])) OR ("hs-CRP"[Title/Abstract])) OR ("CRP"[Title/Abstract]) | 134,050 |
| #22 | Search: "C-Reactive Protein"[Mesh] OR "CRP protein, human" [Supplementary Concept] Sort by: Most Recent | 60,330 |
| #21 | Search: (((("Tumor Necrosis Factor-alpha"[Title/Abstract]) OR ("Tumor Necrosis Factor alpha"[Title/Abstract])) OR ("TNFalpha"[Title/Abstract])) OR ("TNF-alpha"[Title/Abstract])) | 265,340 |
| #20 | Search: "Tumor Necrosis Factor-alpha"[Mesh] Sort by: Most Recent | 146,970 |
| #19 | Search: (((("Interleukin-6"[Title/Abstract]) ) OR ("Interleukin 6"[Title/Abstract])) OR ("IL6"[Title/Abstract])) OR ("IL-6"[Title/Abstract]) | 219,970 |
| #18 | Search: "Interleukin-6"[Mesh] Sort by: Most Recent | 82,925 |
| #17 | Search: "Inflammation"[Title/Abstract] | 751,680 |
| #16 | Search: "Inflammation"[Mesh] Sort by: Most Recent | 469,037 |
| #15 | Search: #11 OR #12 OR #13 OR #14 | 446,665 |
| #14 | Search: ((((((((((((("Cholesterol"[Title/Abstract]) OR ("Epicholesterol"[Title/Abstract])) OR ("Low Density Lipoprotein Cholesterol"[Title/Abstract])) OR ("beta-Lipoprotein Cholesterol"[Title/Abstract])) OR ("beta Lipoprotein Cholesterol"[Title/Abstract])) OR ("LDL Cholesterol"[Title/Abstract])) OR ("LDL Cholesteryl Linoleate"[Title/Abstract])) OR ("alpha-Lipoprotein Cholesterol"[Title/Abstract])) OR ("alpha Lipoprotein Cholesterol"[Title/Abstract])) OR ("HDL Cholesterol"[Title/Abstract])) OR ("High Density Lipoprotein Cholesterol"[Title/Abstract])) ) OR ("HDL-C"[Title/Abstract])) OR ("LDL-C"[Title/Abstract]) | 318,777 |
| #13 | Search: "Cholesterol, LDL"[Mesh] OR "Cholesterol, HDL"[Mesh] OR "Cholesterol"[Mesh] Sort by: Most Recent | 182,458 |
| #12 | Search: "Triglyceride"[Title/Abstract] OR "Triacylglycerol"[Title/Abstract] OR "Triglycerides"[Title/Abstract] OR "Triacylglycerols"[Title/Abstract] | 172,729 |
| #11 | Search: "Triglycerides"[Mesh] Sort by: Most Recent | 90,338 |
| #10 | Search: #5 OR #6 OR #7 OR #8 OR #9 | 1,020,848 |
| #9 | Search: ( (((((("Glycated Hemoglobin"[Title/Abstract]) OR ("hemoglobin a1c protein"[Title/Abstract])) OR ("Glycohemoglobin"[Title/Abstract])) OR ("Glycosylated Hemoglobin"[Title/Abstract])) OR ("Hb A1"[Title/Abstract])) OR ("Hemoglobin A(1)"[Title/Abstract])) OR ("Glycosylated A1b Hemoglobin"[Title/Abstract])) OR ("HbA1c"[Title/Abstract]) | 75,795 |
| #8 | Search: ("Glycated Hemoglobin"[Mesh]) OR "hemoglobin A1c protein, human" [Supplementary Concept] Sort by: Most Recent | 48,736 |
| #7 | Search: (("Insulin"[Title/Abstract]) OR ("Novolin"[Title/Abstract])) OR ("Iletin"[Title/Abstract]) | 449,049 |
| #6 | Search: "Insulin"[Mesh] Sort by: Most Recent | 207,104 |
| #5 | Search: ("Glucose"[MeSH Terms]) OR ("Glucose"[Title/Abstract]) | 732,840 |
| #4 | Search: "Glucose"[Mesh] Sort by: Most Recent | 361,969 |
| #3 | Search: #1 OR #2 | 19,619 |
| #2 | Search: (((((((((("Ubiquinone"[Title/Abstract]) OR ("ubiquinol-10"[Title/Abstract])) OR ("Coenzyme Q"[Title/Abstract])) OR ("CoQ 10"[Title/Abstract])) OR ("co-enzyme Q10"[Title/Abstract])) OR ("ubidecarenone"[Title/Abstract])) OR ("Bio-Quinone Q10"[Title/Abstract])) OR ("CoQ10"[Title/Abstract])) OR ("Q-ter"[Title/Abstract])) OR ("ubisemiquinone"[Title/Abstract])) OR ("coenzyme Q10"[Title/Abstract]) | 16,889 |
| #1 | Search: "Ubiquinone"[Mesh] OR "coenzyme Q10" [Supplementary Concept] OR "ubiquinol-10" [Supplementary Concept] Sort by: Most Recent | 11,170 |

**Web of science**

| Search | Query | Results |
| --- | --- | --- |
| #7 | #1 AND #5 and Clinical Trial (Document Types) and English (Languages) | 269 |
| #6 | #1 AND #5 | 5,950 |
| #5 | #2 OR #3 OR #4 | 5,291,945 |
| #4 | ((((((((((((((((TS=("Inflammation")) OR TS=("Interleukin-6")) OR TS=("Interleukin 6")) OR TS=("IL6")) OR TS=("IL-6")) OR TS=("Tumor Necrosis Factor-alpha")) OR TS=("Tumor Necrosis Factor alpha")) OR TS=("TNFalpha")) OR TS=("TNF-alpha")) OR TS=("C-Reactive Protein")) OR TS=("CRP protein")) OR TS=("C Reactive Protein")) OR TS=("hsCRP")) OR TS=("High Sensitivity C-Reactive Protein")) OR TS=("High Sensitivity C Reactive Protein")) OR TS=("hs-CRP")) OR TS=("CRP") | 2,692,053 |
| #3 | ((((((((((((((((TS=("Triglyceride")) OR TS=( "Triacylglycerol")) OR TS=("Triglycerides")) OR TS=("Triacylglycerols")) OR TS=("Cholesterol")) OR TS=("Epicholesterol")) OR TS=("Low Density Lipoprotein Cholesterol")) OR TS=("beta-Lipoprotein Cholesterol")) OR TS=("beta Lipoprotein Cholesterol")) OR TS=("LDL Cholesterol")) OR TS=("LDL Cholesteryl Linoleate")) OR TS=("alpha-Lipoprotein Cholesterol")) OR TS=("alpha Lipoprotein Cholesterol")) OR TS=("HDL Cholesterol")) OR TS=("High Density Lipoprotein Cholesterol")) OR TS=("HDL-C")) OR TS=("LDL-C") | 857,312 |
| #2 | (((((((((((TS=("Glucose")) OR TS=("Insulin")) OR TS=("Novolin")) OR TS=("Iletin")) OR TS=("Glycated Hemoglobin")) OR TS=("hemoglobin a1c protein")) OR TS=("Glycohemoglobin")) OR TS=("Glycosylated Hemoglobin")) OR TS=("Hb A1")) OR TS=("Hemoglobin A(1)")) OR TS=("Glycosylated A1b Hemoglobin")) OR TS=("HbA1c") | 2,231,831 |
| #1 | ((((((((((TS=("Ubiquinone")) OR TS=("ubiquinol-10")) OR TS=("Coenzyme Q")) OR TS=("CoQ 10")) OR TS=("co-enzyme Q10")) OR TS=("ubidecarenone")) OR TS=("Bio-Quinone Q10")) OR TS=("CoQ10")) OR TS=("Q-ter")) OR TS=("ubisemiquinone")) OR TS=("coenzyme Q10") | 37,269 |

**SCOPUS**

| Search | Query | Results |
| --- | --- | --- |
| #9 | #7 AND #8 | 1,260 |
| #8 | ( TITLE-ABS-KEY ( "clinical trial" ) OR TITLE-ABS-KEY ( "randomized controlled trial" ) OR TITLE-ABS-KEY ( "rct" ) ) | 2,532,819 |
| #7 | #1 AND #5 AND ( LIMIT-TO ( DOCTYPE , "ar" ) ) AND ( LIMIT-TO ( LANGUAGE , "english" ) ) | 6,005 |
| #6 | #1 AND #5 | 8,707 |
| #5 | #2 OR #3 OR #4 | 3,864,561 |
| #4 | ( TITLE-ABS-KEY ( "inflammation" ) OR TITLE-ABS-KEY ( "interleukin-6" ) OR TITLE-ABS-KEY ( "interleukin 6" ) OR TITLE-ABS-KEY ( "il6" ) OR TITLE-ABS-KEY ( "il-6" ) OR TITLE-ABS-KEY ( "tumor necrosis factor-alpha" ) OR TITLE-ABS-KEY ( "tumor necrosis factor alpha" ) OR TITLE-ABS-KEY ( "tnfalpha" ) OR TITLE-ABS-KEY ( "tnf-alpha" ) OR TITLE-ABS-KEY ( "c-reactive protein" ) OR TITLE-ABS-KEY ( "crp protein" ) OR TITLE-ABS-KEY ( "c reactive protein" ) OR TITLE-ABS-KEY ( "hscrp" ) OR TITLE-ABS-KEY ( "high sensitivity c-reactive protein" ) OR TITLE-ABS-KEY ( "high sensitivity c reactive protein" ) OR TITLE-ABS-KEY ( "hs-crp" ) OR TITLE-ABS-KEY ( "crp" ) ) | 1,787,883 |
| #3 | ( TITLE-ABS-KEY ( "triglycerides" ) OR TITLE-ABS-KEY ( "triglyceride" ) OR TITLE-ABS-KEY ( "triacylglycerol" ) OR TITLE-ABS-KEY ( "triacylglycerols" ) OR TITLE-ABS-KEY ( "cholesterol" ) OR TITLE-ABS-KEY ( "epicholesterol" ) OR TITLE-ABS-KEY ( "low density lipoprotein cholesterol" ) OR TITLE-ABS-KEY ( "beta-lipoprotein cholesterol" ) OR TITLE-ABS-KEY ( "beta lipoprotein cholesterol" ) OR TITLE-ABS-KEY ( "ldl cholesterol" ) OR TITLE-ABS-KEY ( "ldl cholesteryl linoleate" ) OR TITLE-ABS-KEY ( "alpha-lipoprotein cholesterol" ) OR TITLE-ABS-KEY ( "alpha lipoprotein cholesterol" ) OR TITLE-ABS-KEY ( "hdl cholesterol" ) OR TITLE-ABS-KEY ( "high density lipoprotein cholesterol" ) OR TITLE-ABS-KEY ( "hdl-c" ) OR TITLE-ABS-KEY ( "ldl-c" ) ) | 753,181 |
| #2 | ( TITLE-ABS-KEY ( "hba1c" ) ) OR ( ( TITLE-ABS-KEY ( "glucose" ) OR TITLE-ABS-KEY ( "insulin" ) OR TITLE-ABS-KEY ( "novolin" ) OR TITLE-ABS-KEY ( "iletin" ) OR TITLE-ABS-KEY ( "glycated hemoglobin" ) OR TITLE-ABS-KEY ( "hemoglobin a1c protein" ) OR TITLE-ABS-KEY ( "glycohemoglobin" ) OR TITLE-ABS-KEY ( "glycosylated hemoglobin" ) OR TITLE-ABS-KEY ( "hb a1" ) OR TITLE-ABS-KEY ( "hemoglobin a(1)" ) OR TITLE-ABS-KEY ( "glycosylated a1b hemoglobin" ) ) ) | 1,780,499 |
| #1 | ( TITLE-ABS-KEY ( "ubiquinone" ) OR TITLE-ABS-KEY ( "ubiquinol-10" ) OR TITLE-ABS-KEY ( "coenzyme q" ) OR TITLE-ABS-KEY ( "coq 10" ) OR TITLE-ABS-KEY ( "co-enzyme q10" ) OR TITLE-ABS-KEY ( "ubidecarenone" ) OR TITLE-ABS-KEY ( "bio-quinone q10" ) OR TITLE-ABS-KEY ( "coq10" ) OR TITLE-ABS-KEY ( "q-ter" ) OR TITLE-ABS-KEY ( "ubisemiquinone" ) OR TITLE-ABS-KEY ( "coenzyme q10" ) ) | 42,945 |

**Cochrane Library**

#1 MeSH descriptor: [Ubiquinone] explode all trees 719

#2 (Ubiquinone):ti,ab,kw OR (ubiquinol-10):ti,ab,kw OR ("Coenzyme Q"):ti,ab,kw OR ("CoQ 10"):ti,ab,kw OR ("co-enzyme Q10"):ti,ab,kw (Word variations have been searched) 939

#3 (ubidecarenone):ti,ab,kw OR ("Bio-Quinone Q10"):ti,ab,kw OR (CoQ10):ti,ab,kw OR (Q-ter):ti,ab,kw (Word variations have been searched) 788

#4 ("coenzyme Q10"):ti,ab,kw OR (ubisemiquinone):ti,ab,kw (Word variations have been searched) 1254

#5 #1 OR #2 OR #3 OR #4 1715

#6 MeSH descriptor: [Glucose] explode all trees 26031

#7 MeSH descriptor: [Insulins] explode all trees 18650

#8 MeSH descriptor: [Glycated Hemoglobin] explode all trees 8748

#9 (Glucose):ti,ab,kw OR (Insulin):ti,ab,kw OR (Novolin):ti,ab,kw OR (Iletin):ti,ab,kw OR ("Glycated Hemoglobin"):ti,ab,kw (Word variations have been searched) 122812

#10 ("hemoglobin a1c protein"):ti,ab,kw OR (Glycohemoglobin):ti,ab,kw OR ("Glycosylated Hemoglobin"):ti,ab,kw OR ("HbA1c"):ti,ab,kw OR (Hemoglobin A(1)):ti,ab,kw (Word variations have been searched) 35393

#11 ("Glycosylated A1b Hemoglobin"):ti,ab,kw (Word variations have been searched) 0

#12 #6 OR #7 OR #8 OR #9 OR #10 OR #11 142291

#13 MeSH descriptor: [Triglycerides] explode all trees 7935

#14 MeSH descriptor: [Cholesterol] explode all trees 12893

#15 ("LDL Cholesterol"):ti,ab,kw OR ("LDL Cholesteryl Linoleate"):ti,ab,kw OR ("alpha Lipoprotein Cholesterol"):ti,ab,kw OR ("HDL Cholesterol"):ti,ab,kw OR ("High Density Lipoprotein Cholesterol"):ti,ab,kw (Word variations have been searched) 20477

#16 ("HDL-C"):ti,ab,kw OR ("LDL-C"):ti,ab,kw (Word variations have been searched) 10958

#17 #13 OR #14 OR #15 OR #16 34555

#18 MeSH descriptor: [Inflammation] explode all trees 16991

#19 MeSH descriptor: [Interleukin-6] explode all trees 4484

#20 MeSH descriptor: [Tumor Necrosis Factor-alpha] explode all trees 4379

#21 MeSH descriptor: [C-Reactive Protein] explode all trees 6452

#22 (Inflammation):ti,ab,kw OR (Interleukin-6):ti,ab,kw OR ("Interleukin 6"):ti,ab,kw OR (IL6):ti,ab,kw OR (IL-6):ti,ab,kw (Word variations have been searched) 76606

#23 ("Tumor Necrosis Factor-alpha"):ti,ab,kw OR ("Tumor Necrosis Factor alpha"):ti,ab,kw OR (TNFalpha):ti,ab,kw OR (TNF-alpha):ti,ab,kw (Word variations have been searched) 10193

#24 ("High Sensitivity C Reactive Protein"):ti,ab,kw OR (hs-CRP):ti,ab,kw OR (CRP):ti,ab,kw (Word variations have been searched) 28406

#25 #18 OR #19 OR #20 OR #21 OR #22 OR #23 OR #24 103160

#26 #12 OR #17 OR #25 248720

#27 #5 AND #26 495

#28 #5 AND #26 in Trials 493

**Embase**

1 "Ubiquinone".mp. or exp ubiquinone/ 31682

2 "ubidecarenone".mp. or exp ubidecarenone/ 13038

3 ("Ubiquinone" or "ubiquinol-10" or "Coenzyme Q" or "CoQ 10" or "co-enzyme Q10" or "ubidecarenone" or "Bio-Quinone Q10" or "CoQ10" or "Q-ter" or "ubisemiquinone" or "coenzyme Q10").ab,ti. 18810

4 1 or 2 or 3 43609

5 "Glucose".mp. or exp glucose/ 1197860

6 exp insulin/ or exp human insulin/ or "Insulin".mp. 1126719

7 "Glycated Hemoglobin".mp. or exp glycosylated hemoglobin/ 230344

8 ("Glucose" or "Insulin" or "Novolin" or "Iletin" or "Glycated Hemoglobin" or "hemoglobin a1c protein" or "Glycohemoglobin" or "Glycosylated Hemoglobin" or "HbA1c" or "Hemoglobin A1" or "Glycosylated A1b Hemoglobin").ab,ti. 1245759

9 5 or 6 or 7 or 8 1868437

10 "Triglycerides".mp. or exp triacylglycerol/ 336873

11 exp low density lipoprotein cholesterol/ or exp cholesterol/ or "Cholesterol".mp. or exp high density lipoprotein cholesterol/ 601543

12 ("Triglyceride" or "Triacylglycerol" or "Triglycerides" or "Triacylglycerols" or "Cholesterol" or "Epicholesterol" or "Low Density Lipoprotein Cholesterol" or "beta-Lipoprotein Cholesterol" or "beta Lipoprotein Cholesterol" or "LDL Cholesterol" or "LDL Cholesteryl Linoleate" or "alpha-Lipoprotein Cholesterol" or "alpha Lipoprotein Cholesterol" or "HDL Cholesterol" or "High Density Lipoprotein Cholesterol" or "HDL-C" or "LDL-C").ab,ti. 529917

13 10 or 11 or 12 733543

14 exp inflammation/ or "Inflammation".mp. 6658379

15 "Tumor Necrosis Factor-alpha".mp. or exp tumor necrosis factor/ 528589

16 "C Reactive Protein".mp. or exp C reactive protein/ 347733

17 exp interleukin 6/ or "Interleukin 6".mp. 431700

18 ("Inflammation" or "Interleukin-6" or "Interleukin 6" or "IL6" or "IL-6" or "Tumor Necrosis Factor-alpha" or "Tumor Necrosis Factor alpha" or "TNFalpha" or "TNF-alpha" or "C-Reactive Protein" or "CRP protein" or "C Reactive Protein" or "hsCRP" or "High Sensitivity C-Reactive Protein" or "High Sensitivity C Reactive Protein" or "hs-CRP" or "CRP").ab,ti. 1526244

19 14 or 15 or 16 or 17 or 18 7075751

20 9 or 13 or 19 8446098

21 4 and 20 11937

22 limit 21 to (human and english language and randomized controlled trial) 447

**ClinicalTrials.gov**

Search Details: Viewing 1-40 out of 40 studies for: Ubiquinone OR ubiquinol OR Coenzyme Q OR CoQ 10 OR Q10 | Interventional studies | Studies with results
