# Supplementary material for: Effects of Coenzyme Q10 on Lipid, Glycemic, and Inflammatory Markers in Metabolic Disorders: A Systematic Review and Meta‐Analysis
Source: J Diabetes Res. 2026 May 26;2026:5587445. doi: 10.1155/jdr/5587445 (PMC13212042; doi:10.1155/jdr/5587445)
Supplement: Supplementary file 3 — Supporting Information 3 Supporting File S3: Univariate and interaction meta‐regression analyses to investigate the potential impact of various moderators on pooled estimates. [file JDR-2026-5587445-s002.docx]

**Supplementary file 3: Univariate and interaction meta-regression analyses to investigate the potential impact of various moderators on pooled estimates**

| **Outcome** | **Covariate** | **No. of effect sizes (k)** | **Coefficient**  **(β)** | **95% CI** | ***P*-value** | **I²_res**  **(%)** | **τ²**  **(REML)** | **Adj *R²***  **(%)** |
| --- | --- | --- | --- | --- | --- | --- | --- | --- |
| TG (mg/dL) | Dosage (mg/day) | 42 | 0.024 | (-0.047, 0.095) | 0.500 | 37.42 | 67.08 | -32.13 |
|  | Duration (weeks) | 42 | -0.839 | (-2.355, 0.677) | 0.270 | 39.79 | 51.65 | -1.73 |
|  | Age (years) | 42 | 0.060 | (-0.408, 0.528) | 0.797 | 40.16 | 59.47 | -17.13 |
|  | Baseline BMI (kg/m²) | 42 | -1.806 | (-3.767, 0.083) | 0.070 | 26.83 | 36.09 | 28.91 |
|  | Baseline TG (mg/dL) | 42 | -0.020 | (-0.124, 0.084) | 0.696 | 32.55 | 56.22 | -10.74 |
|  | Dosage × Duration | 42 | 0.007 | (-0.010, 0.024) | 0.433 | 38.73 | 73.08 | -43.94 |
| TC (mg/dL) | Dosage (mg/day) | 48 | 0.016 | (-0.037, 0.064) | 0.546 | 72.13 | 117.70 | -3.08 |
|  | Duration (weeks) | 48 | -0.178 | (-1.065, 0.710) | 0.689 | 70.86 | 117.60 | -3.00 |
|  | Age (years) | 48 | 0.105 | (-0.339, 0.548) | 0.637 | 71.20 | 119.20 | -4.46 |
|  | Baseline BMI (kg/m²) | 48 | -0.531 | (-2.576, 1.515) | 0.604 | 71.78 | 118.20 | -3.51 |
|  | Baseline TC (mg/dL) | 47 | -0.005 | (-0.119, 0.110) | 0.935 | 69.38 | 122.80 | -3.95 |
|  | Dosage × Duration | 48 | 0.001 | (-0.008, 0.009) | 0.867 | 71.98 | 126.10 | -10.48 |
| HDL-C (mg/dL) | Dosage (mg/day) | 48 | 0.006 | (-0.009, 0.020) | 0.426 | 81.12 | 7.73 | 0.15 |
|  | Duration (weeks) | 48 | -0.055 | (-0.310, 0.201) | 0.667 | 79.22 | 7.91 | -2.10 |
|  | Age (years) | 48 | 0.077 | (-0.032, 0.186) | 0.162 | 80.21 | 7.68 | 0.86 |
|  | Baseline BMI (kg/m²) | 48 | -0.095 | (-0.612, 0.422) | 0.712 | 80.26 | 7.86 | -1.44 |
|  | Baseline HDL-C (mg/dL) | 47 | 0.070 | (-0.062, 0.202) | 0.288 | 75.61 | 7.50 | 4.33 |
|  | Dosage × Duration | 48 | 0.001 | (-0.002, 0.003) | 0.613 | 80.04 | 8.06 | -3.97 |
| LDL-C (mg/dL) | Dosage (mg/day) | 50 | 0.019 | (-0.022, 0.059) | 0.365 | 78.29 | 145.00 | -2.32 |
|  | Duration (weeks) | 50 | 0.027 | (-0.878, 0.931) | 0.953 | 78.48 | 145.80 | -2.91 |
|  | Age (years) | 50 | 0.086 | (-0.327, 0.499) | 0.678 | 78.34 | 146.50 | -3.40 |
|  | Baseline BMI (kg/m²) | 50 | -0.273 | (-2.212, 1.667) | 0.779 | 78.46 | 145.90 | -3.01 |
|  | Baseline LDL-C (mg/dL) | 49 | -0.107 | (-0.239, 0.026) | 0.110 | 74.21 | 136.00 | 6.80 |
|  | Dosage × Duration | 50 | 0.006 | (-0.003, 0.014) | 0.197 | 79.06 | 146.70 | -3.57 |
| HbA1c (%) | Dosage (mg/day) | 31 | 0.002 | (-0.000, 0.004) | 0.074 | 68.14 | 0.11 | -0.57 |
|  | Duration (weeks) | 31 | 0.039 | (-0.002, 0.079) | 0.059 | 66.91 | 0.11 | 4.86 |
|  | Age (years) | 31 | -0.002 | (-0.023, 0.018) | 0.811 | 66.75 | 0.12 | -7.41 |
|  | Baseline BMI (kg/m²) | 31 | -0.067 | (-0.148, 0.015) | 0.105 | 62.56 | 0.10 | 7.50 |
|  | Dosage × Duration | 31 | 0.000 | (-0.000, 0.001) | 0.842 | 68.29 | 0.10 | 5.75 |
| Fasting glucose (mg/dL) | Dosage (mg/day) | 39 | 0.033 | (-0.018, 0.085) | 0.196 | 85.39 | 126.10 | 2.82 |
|  | Duration (weeks) | 39 | 0.191 | (-0.777, 1.159) | 0.692 | 83.22 | 134.70 | -3.76 |
|  | Age (years) | 39 | -0.209 | (-0.688, 0.270) | 0.383 | 87.90 | 133.00 | -2.44 |
|  | Baseline BMI (kg/m²) | 39 | 0.316 | (-1.613, 2.245) | 0.742 | 75.94 | 132.20 | -1.84 |
|  | Dosage × Duration | 39 | -0.007 | (-0.018, 0.003) | 0.159 | 79.04 | 125.80 | 3.04 |
| FINS (µIU/mL) | Dosage (mg/day) | 17 | 0.012 | (-0.020, 0.043) | 0.453 | 92.17 | 28.72 | -3.58 |
|  | Duration (weeks) | 17 | 0.117 | (-0.600, 0.833) | 0.733 | 90.94 | 29.76 | -7.33 |
|  | Age (years) | 17 | 0.025 | (-0.300, 0.349) | 0.873 | 91.12 | 29.80 | -7.49 |
|  | Baseline BMI (kg/m²) | 17 | 1.003 | (-0.025, 2.032) | 0.055 | 89.95 | 22.40 | 19.22 |
|  | Dosage × Duration | 17 | 0.004 | (-0.017, 0.026) | 0.680 | 92.07 | 32.78 | -18.23 |
| HOMA-IR | Dosage (mg/day) | 11 | -0.001 | (-0.011, 0.010) | 0.882 | 64.87 | 0.27 | -12.72 |
|  | Duration (weeks) | 11 | -0.037 | (-0.117, 0.043) | 0.321 | 71.67 | 0.21 | 9.17 |
|  | Age (years) | 11 | -0.028 | (-0.070, 0.014) | 0.164 | 79.24 | 0.19 | 17.81 |
|  | Baseline BMI (kg/m²) | 11 | 0.022 | (-0.218, 0.261) | 0.841 | 80.45 | 0.25 | -7.01 |
|  | Dosage × Duration | 11 | 0.000 | (-0.004, 0.005) | 0.879 | 59.67 | 0.33 | -39.38 |
| CRP (mg/L) | Dosage (mg/day) | 15 | 0.003 | (-0.001, 0.007) | 0.182 | 90.85 | 0.37 | 4.99 |
|  | Duration (weeks) | 15 | 0.007 | (-0.082, 0.096) | 0.865 | 91.32 | 0.43 | -10.51 |
|  | Age (years) | 15 | 0.008 | (-0.020, 0.036) | 0.544 | 90.56 | 0.42 | -5.49 |
|  | Baseline BMI (kg/m²) | 15 | -0.164 | (-0.428, 0.101) | 0.204 | 90.33 | 0.36 | 7.68 |
|  | Dosage × Duration | 15 | -0.000 | (-0.002, 0.002) | 0.969 | 92.15 | 0.45 | -16.93 |
| IL-6 | Dosage (mg/day) | 11 | 0.000 | (-0.005, 0.005) | 0.950 | 76.51 | 0.33 | -14.43 |
|  | Duration (weeks) | 11 | 0.051 | (-0.199, 0.302) | 0.653 | 76.11 | 0.33 | -11.80 |
|  | Age (years) | 11 | -0.007 | (-0.042, 0.029) | 0.680 | 75.75 | 0.33 | -11.29 |
|  | Baseline BMI (kg/m²) | 11 | -0.003 | (-0.235, 0.229) | 0.980 | 76.58 | 0.33 | -14.57 |
|  | Dosage × Duration | 11 | -0.002 | (-0.004, 0.000) | 0.094 | 70.27 | 0.25 | 14.57 |

**Notes**: In cases where the quantitative measurements for the mean age or baseline BMI were not reported, the values were imputed from the pooled median of the remaining trials. Dosage×Duration represents the interaction between daily dose and treatment duration.
