# Supplementary material for: Effects of Coenzyme Q10 on Lipid, Glycemic, and Inflammatory Markers in Metabolic Disorders: A Systematic Review and Meta‐Analysis
Source: J Diabetes Res. 2026 May 26;2026:5587445. doi: 10.1155/jdr/5587445 (PMC13212042; doi:10.1155/jdr/5587445)
Supplement: Supplementary file 5 — Supporting Information 5 Figure S2: Meta‐regressions to investigate the relationship between duration and estimated net changes in outcome indicators: (A) TG, (B) TC, (C) HDL‐C, (D) LDL‐C, (E) HbA1c, (F) fasting glucose, (G) FINS, (H) HOMA‐IR, (I) CPR, and (J) IL‐6. [file JDR-2026-5587445-s004.docx]

**Supplementary file 5: Meta-regressions to investigate the relationship between duration and estimated net changes in outcome indicators**

**Fig.S2.A TG Fig.S2.B TC**

**
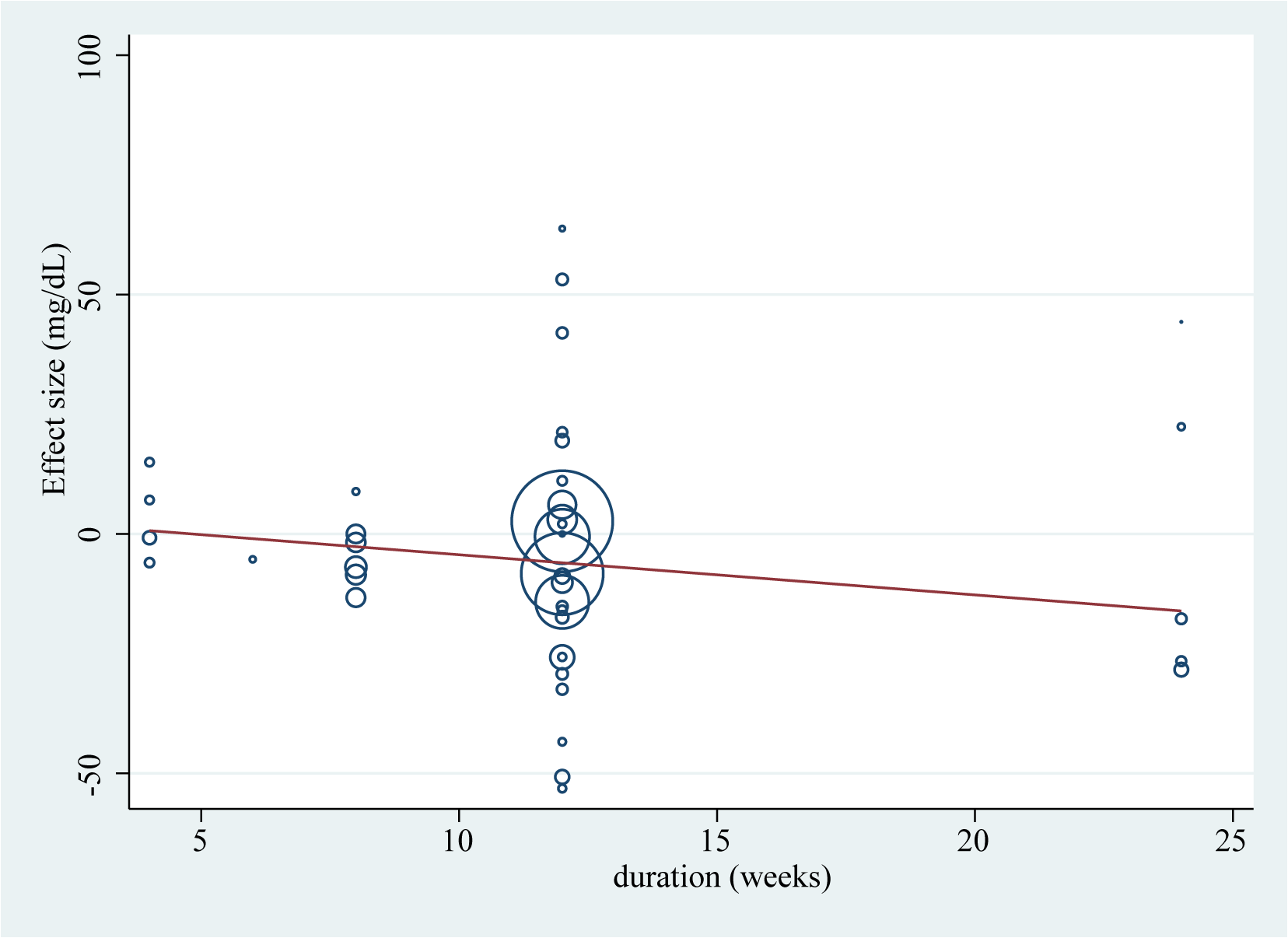

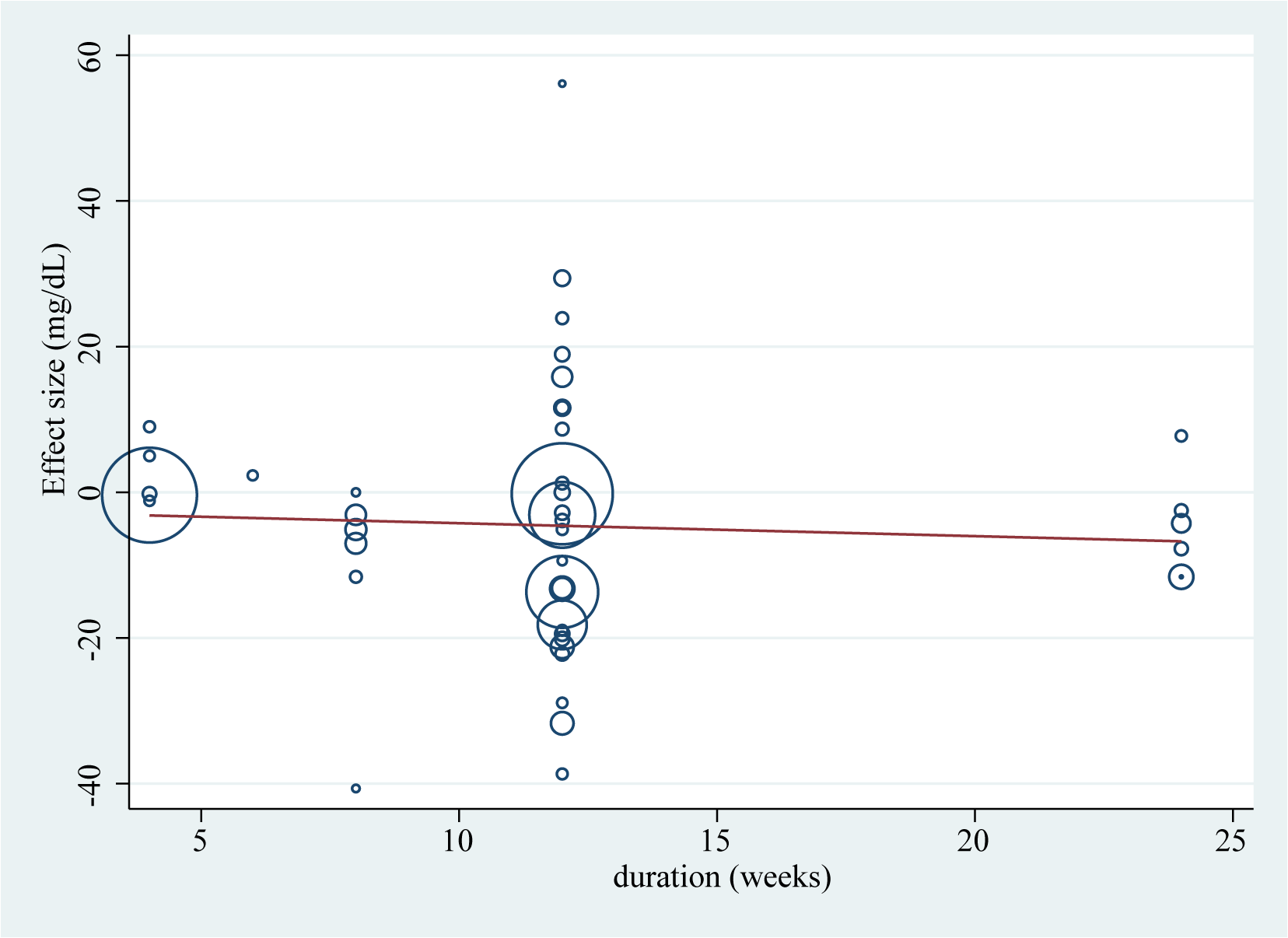
**

**Fig.S2.C HDL-C Fig.S2.D LDL-C**

**
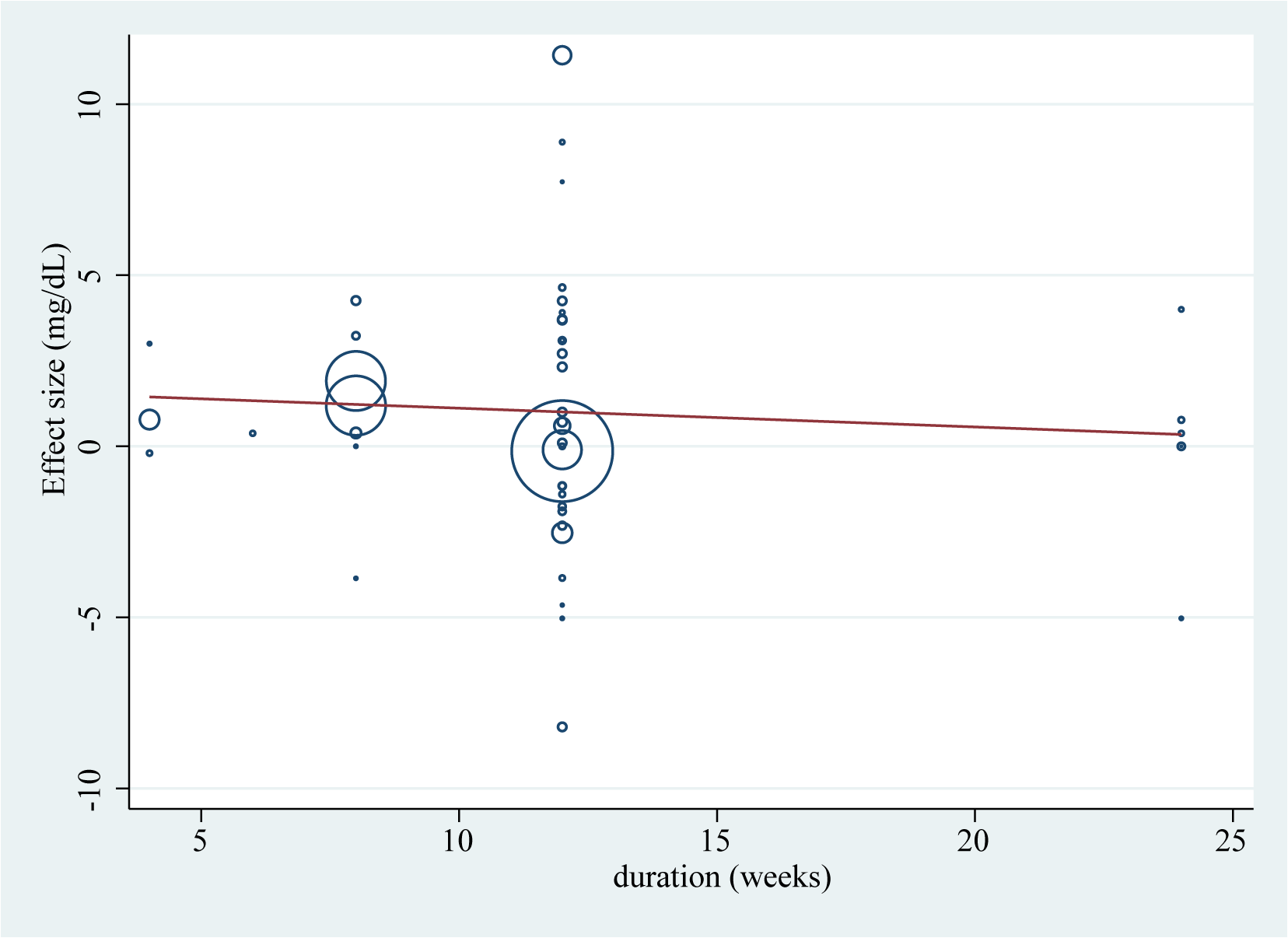

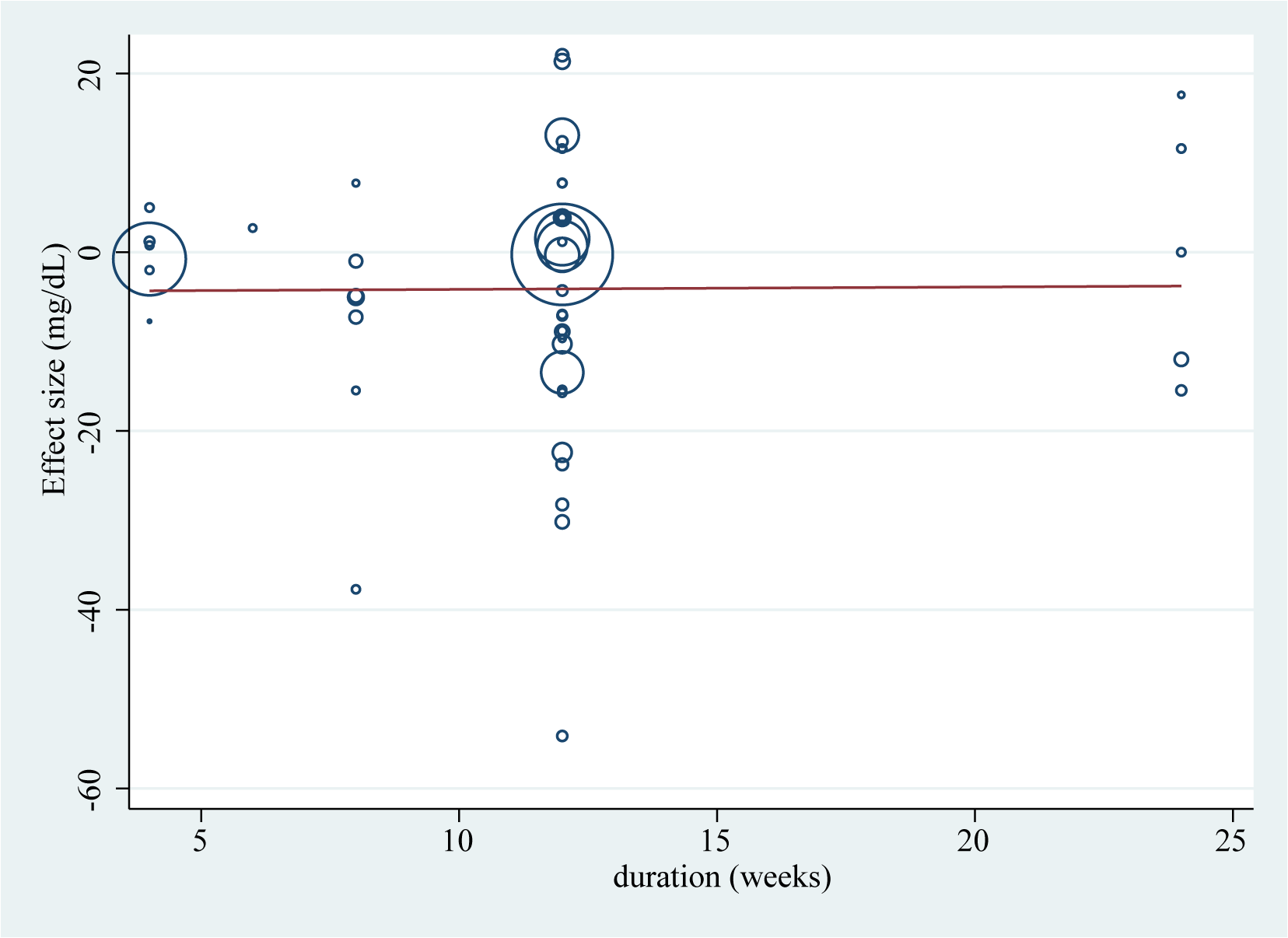
**

**Fig.S2.E HbA1c Fig.S2.F Fasting glucose**

**
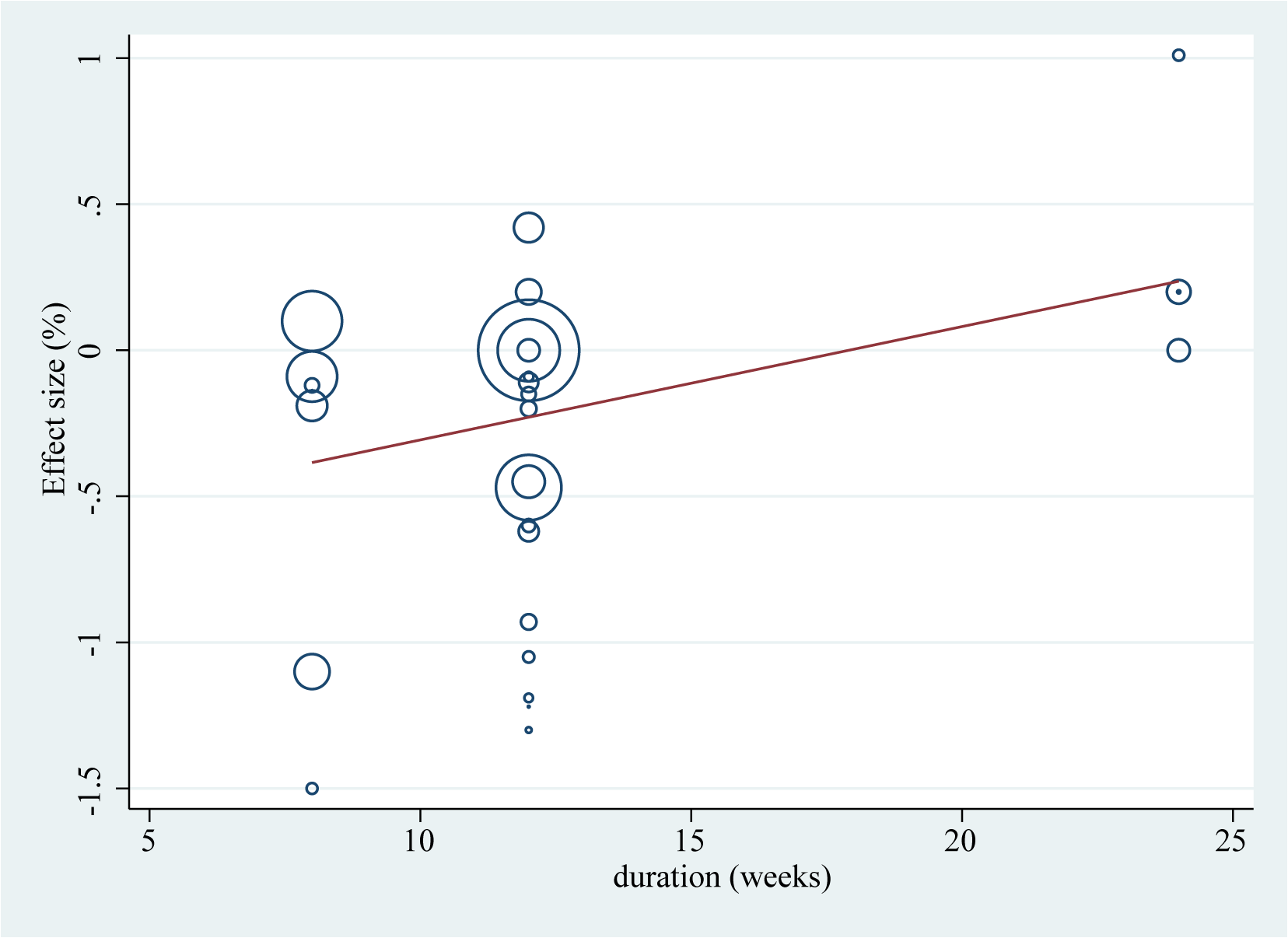

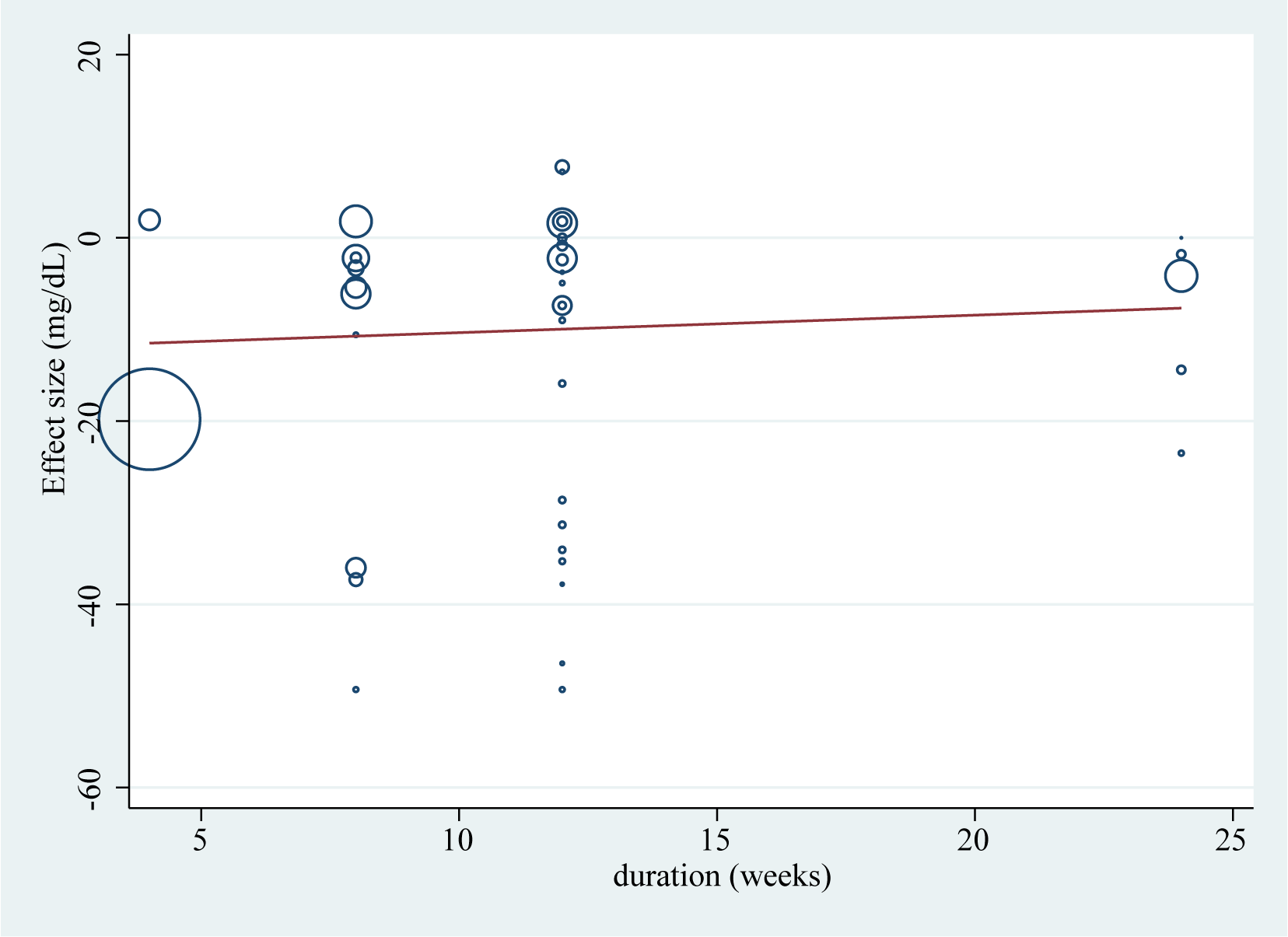
**

**Fig.S2.G FINS Fig.S2.H HOMA-IR**

**
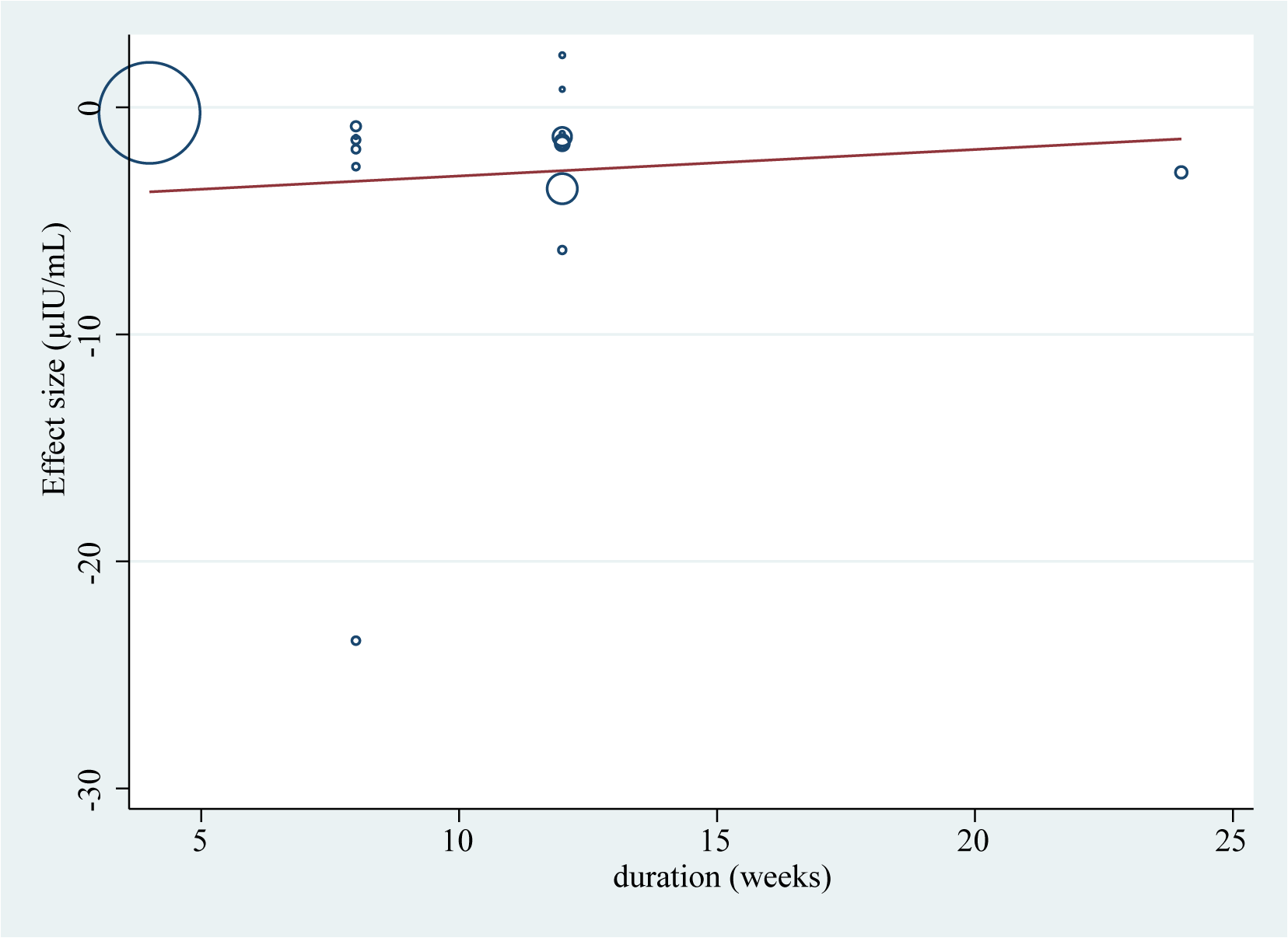

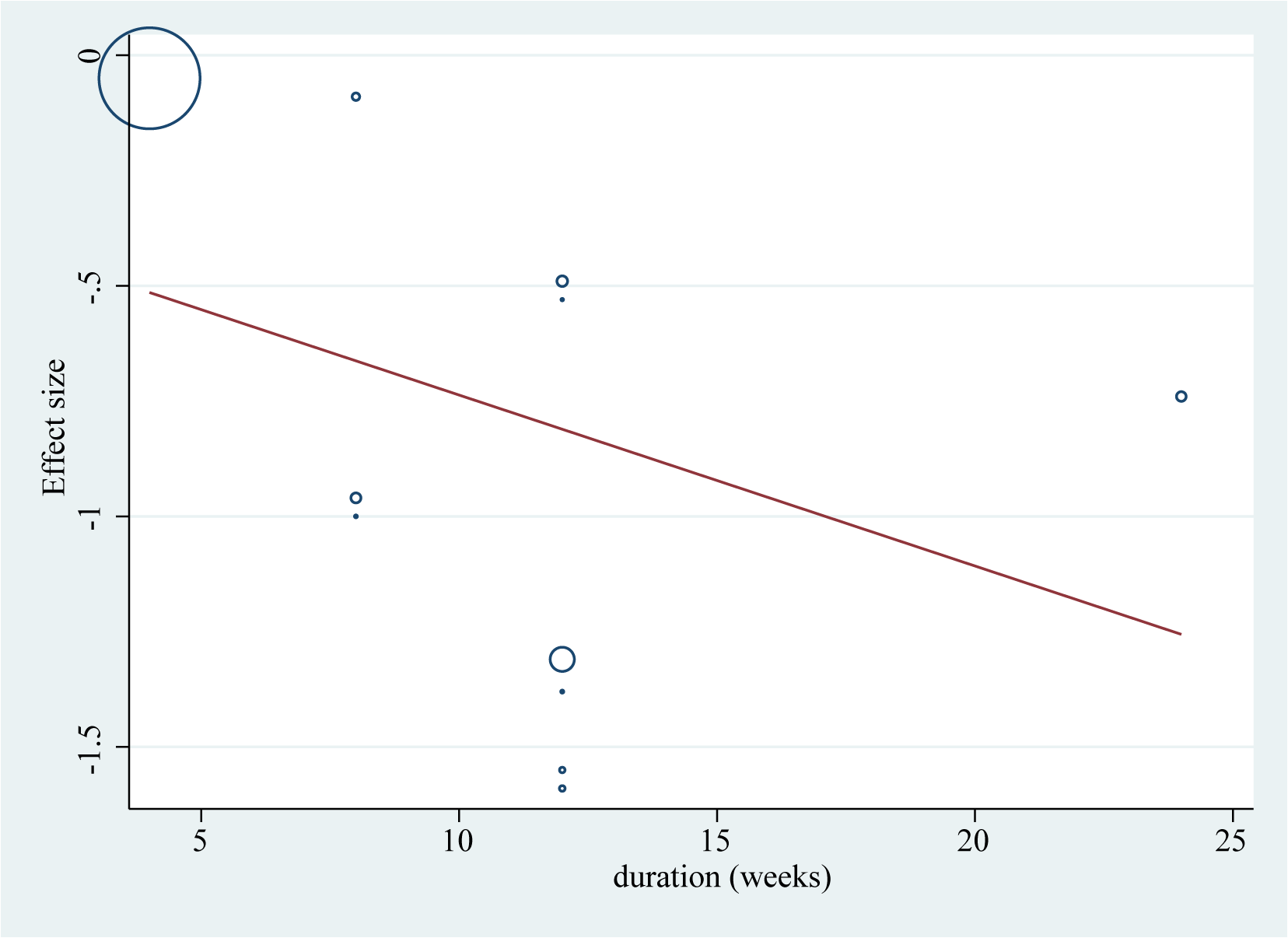
**

**Fig.S2.I CPR Fig.S2.J IL-6**

**
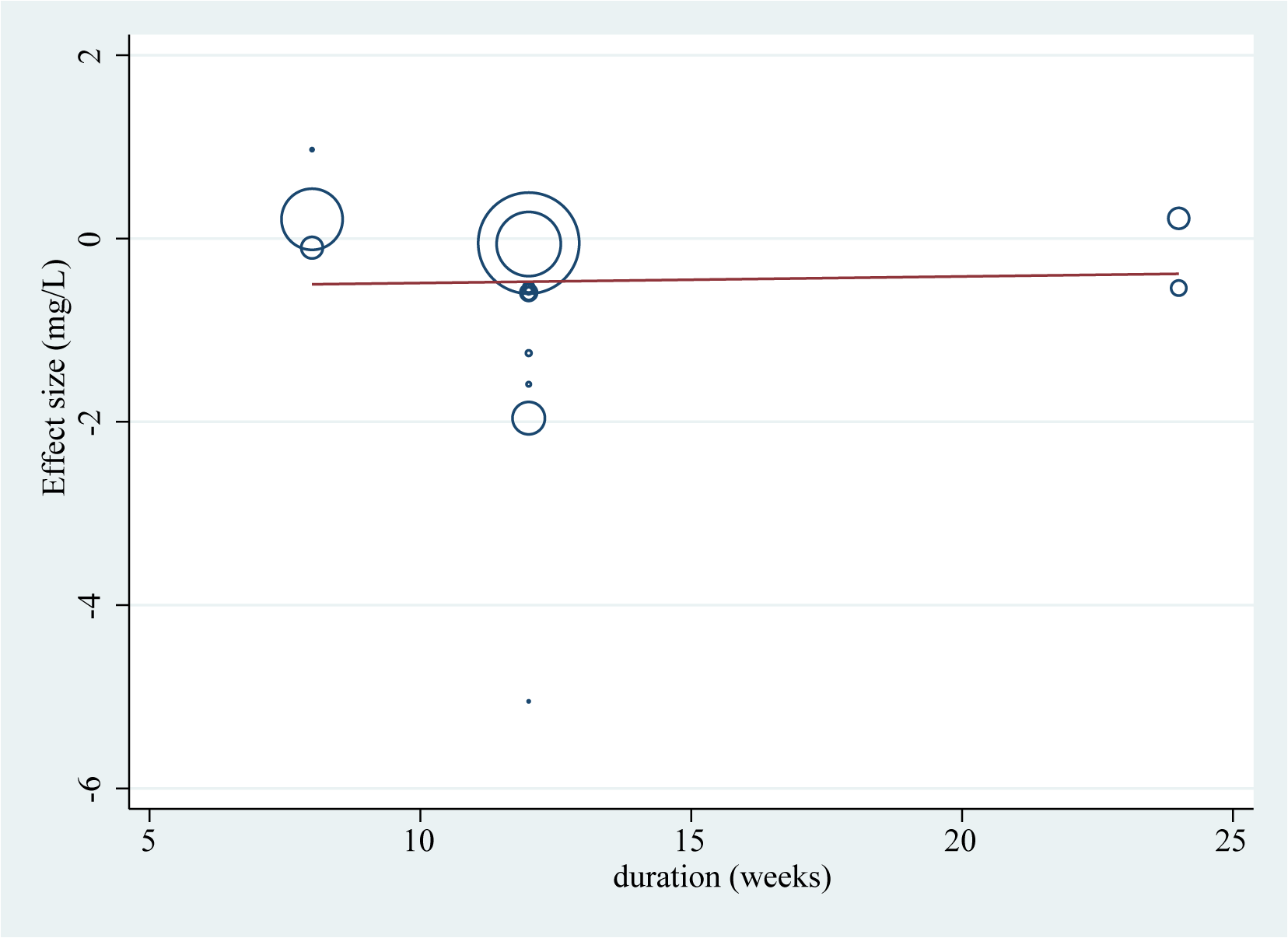

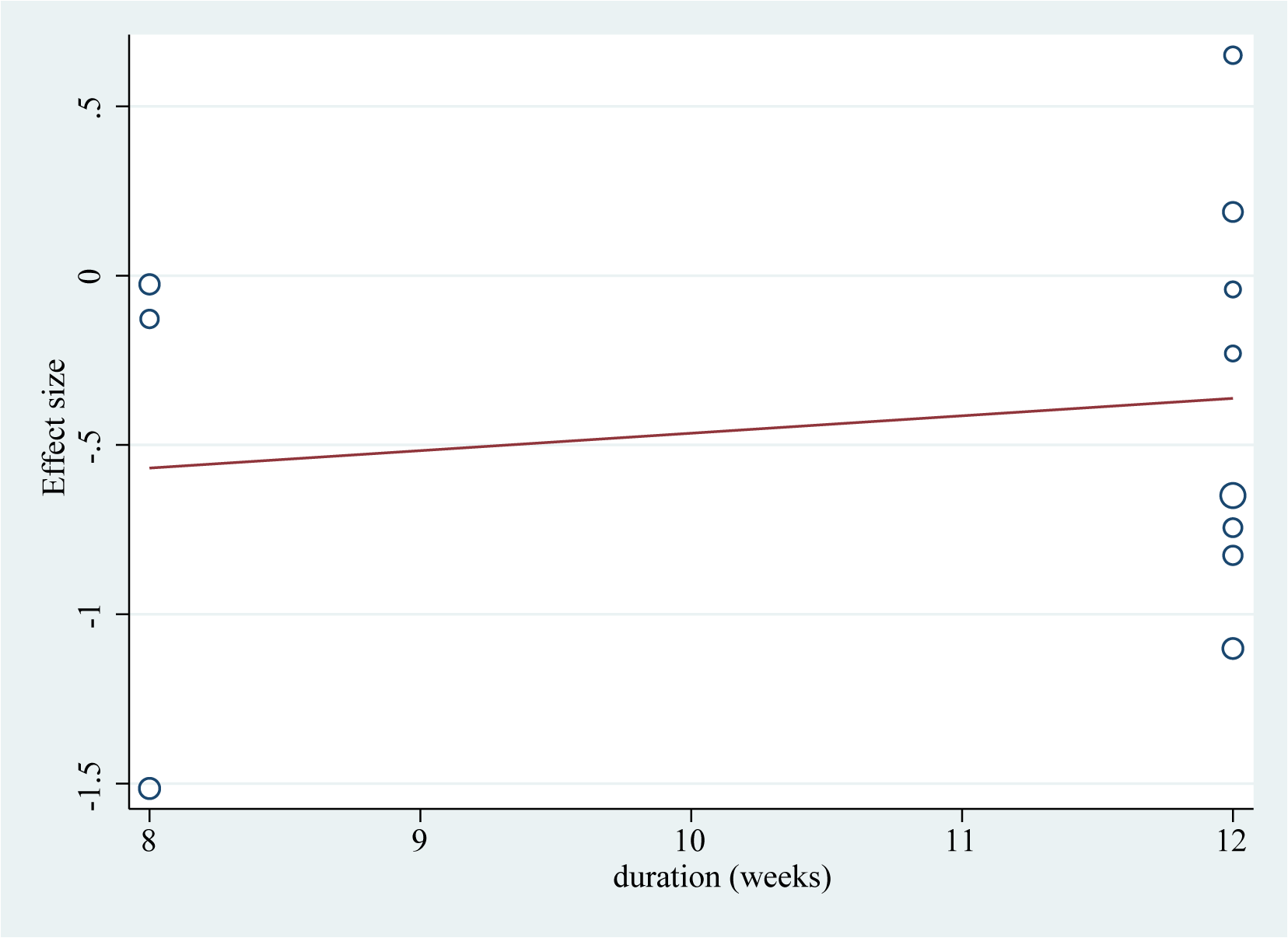
**

**Notes:** Random-effects meta-regression was performed using REML with Knapp–Hartung adjustment. Bubble size is proportional to the inverse-variance weight (1/SE²).
