# Supplementary material for: Effects of Coenzyme Q10 on Lipid, Glycemic, and Inflammatory Markers in Metabolic Disorders: A Systematic Review and Meta‐Analysis
Source: J Diabetes Res. 2026 May 26;2026:5587445. doi: 10.1155/jdr/5587445 (PMC13212042; doi:10.1155/jdr/5587445)

**Supplementary file 6: Funnel plots**

**Fig.S3.A TG**  **Fig.S3.B TC**


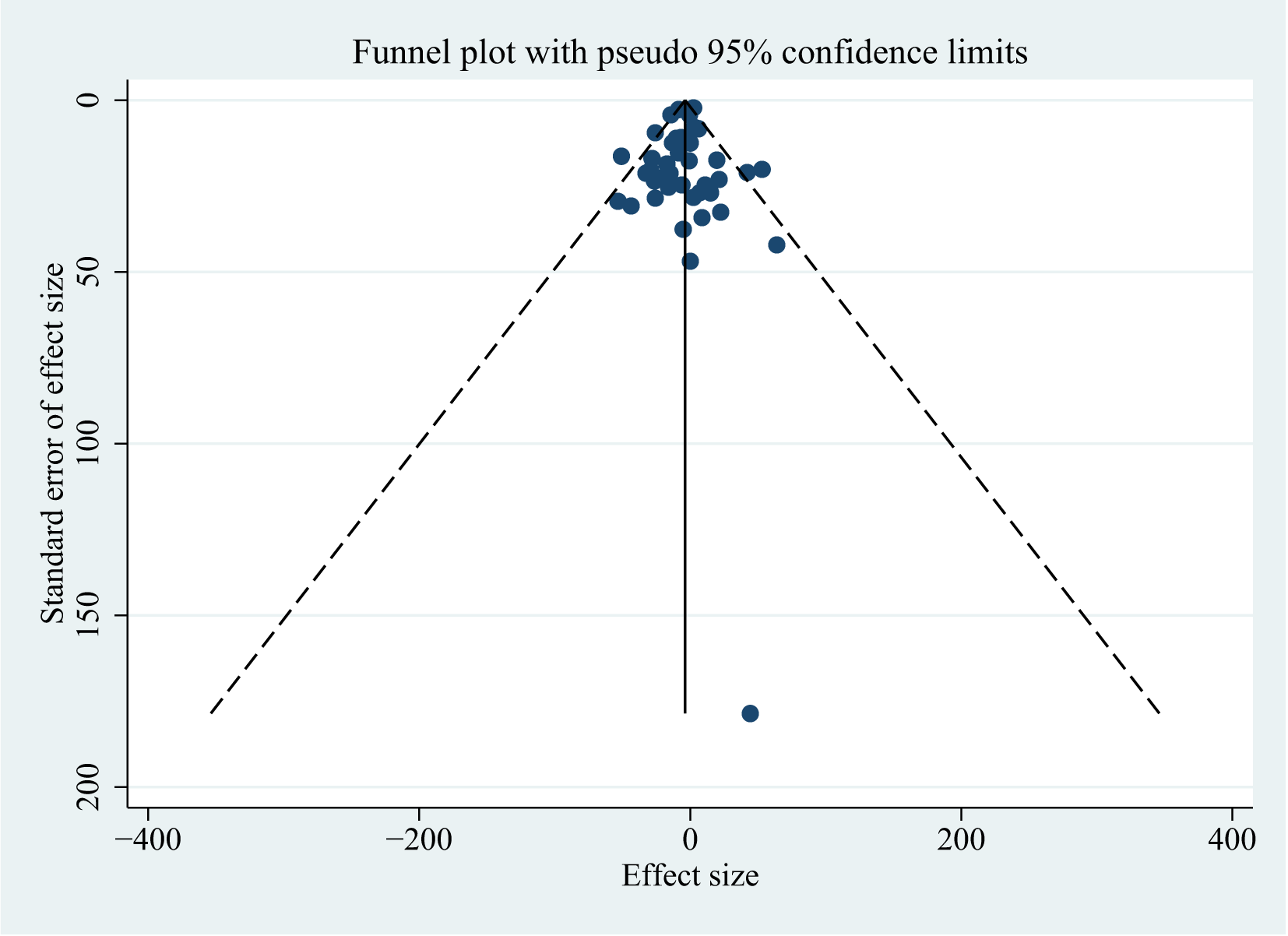

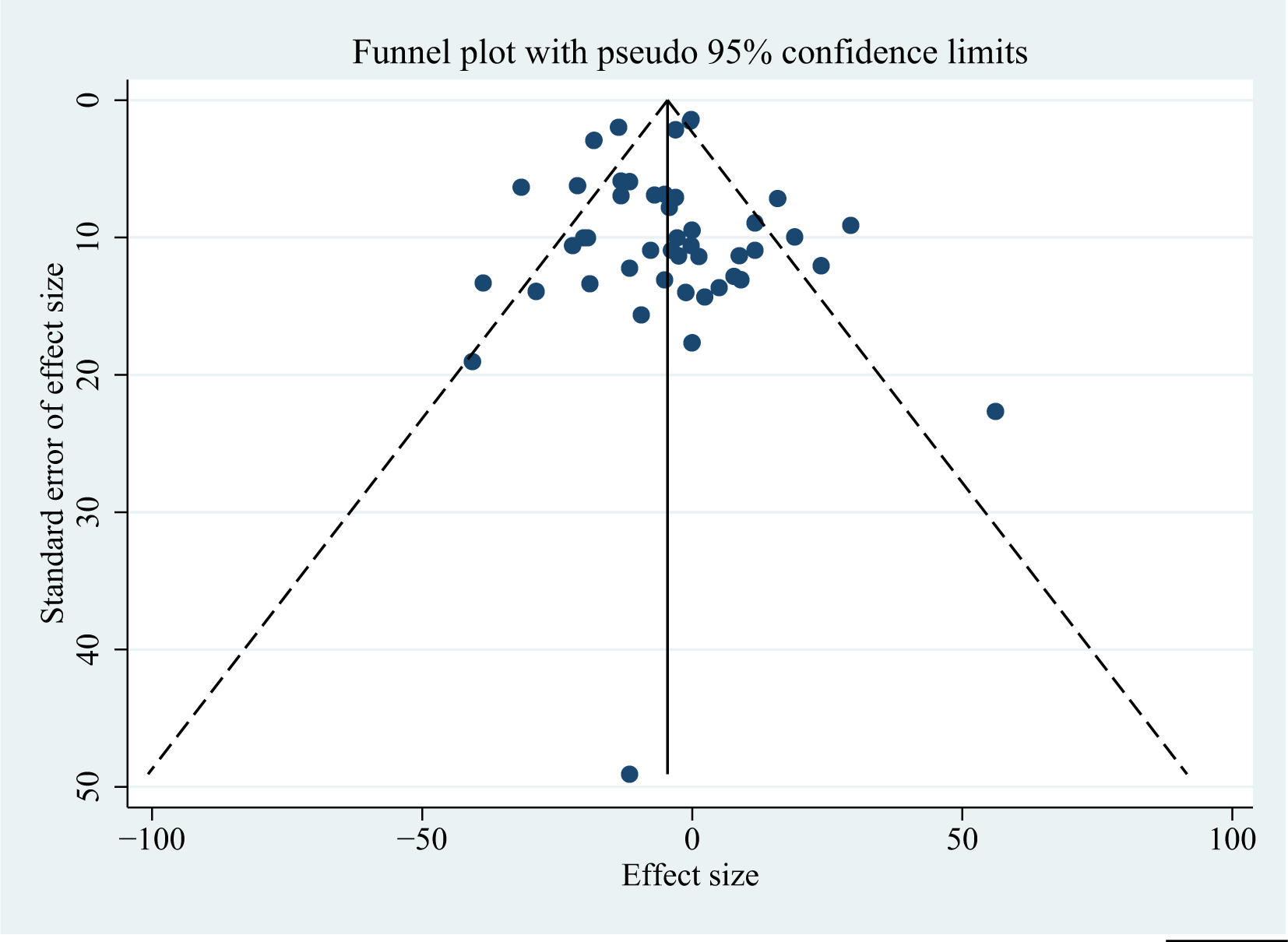


**Fig.S3.C HDL-C**  **Fig.S3.D LDL-C**


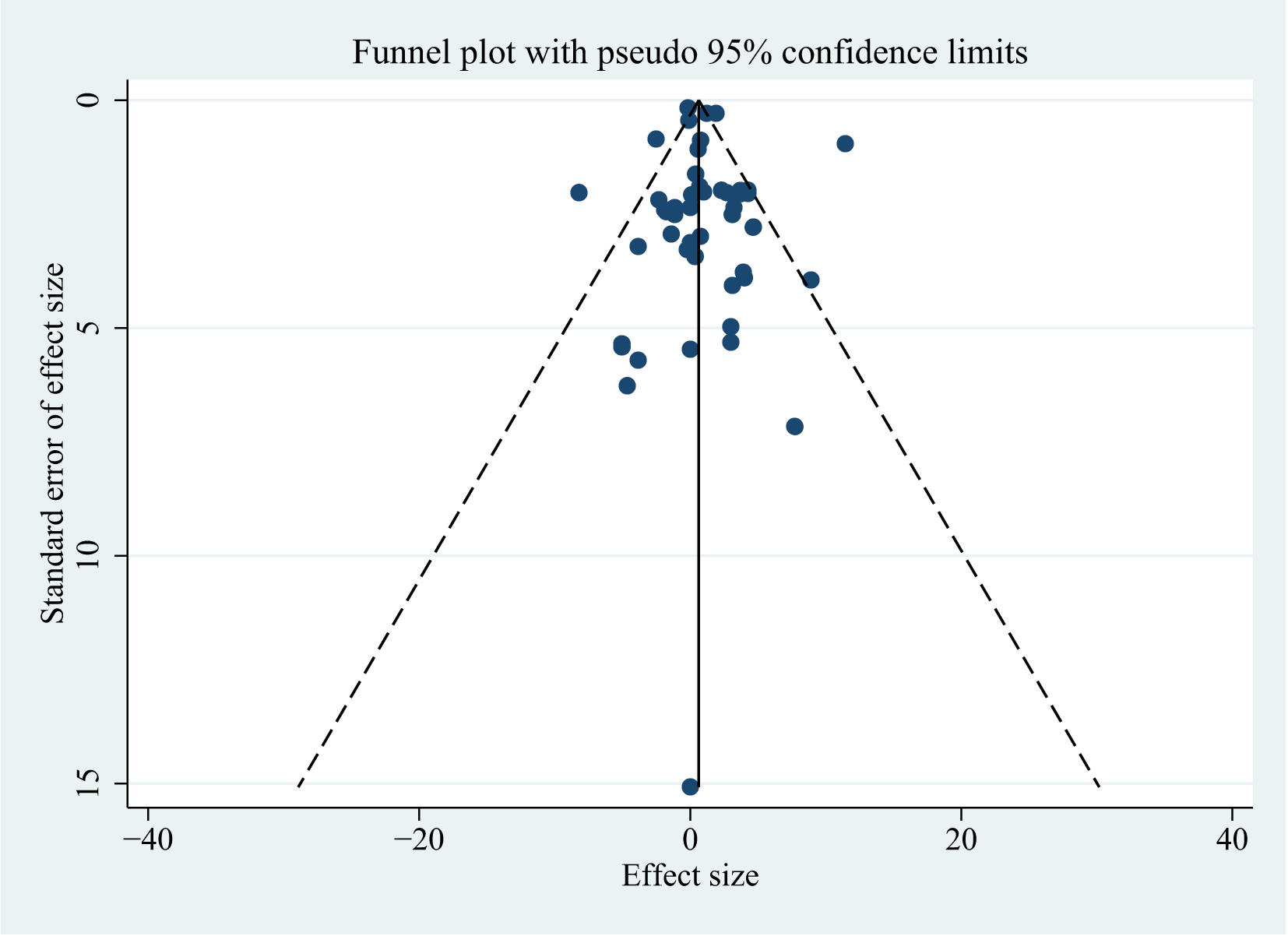

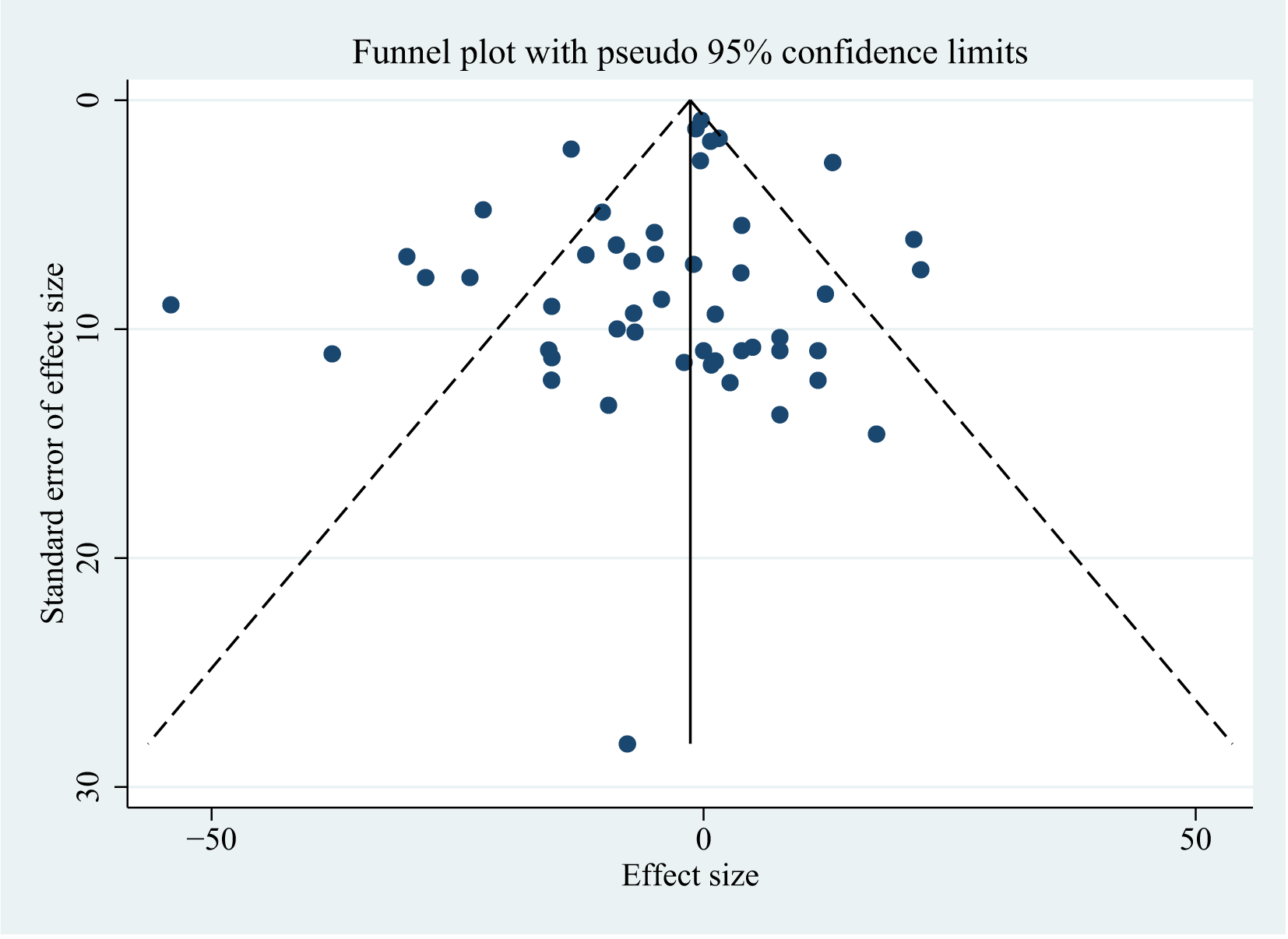


**Fig.S3.E HbA1c**  **Fig.S3.F Fasting glucose**


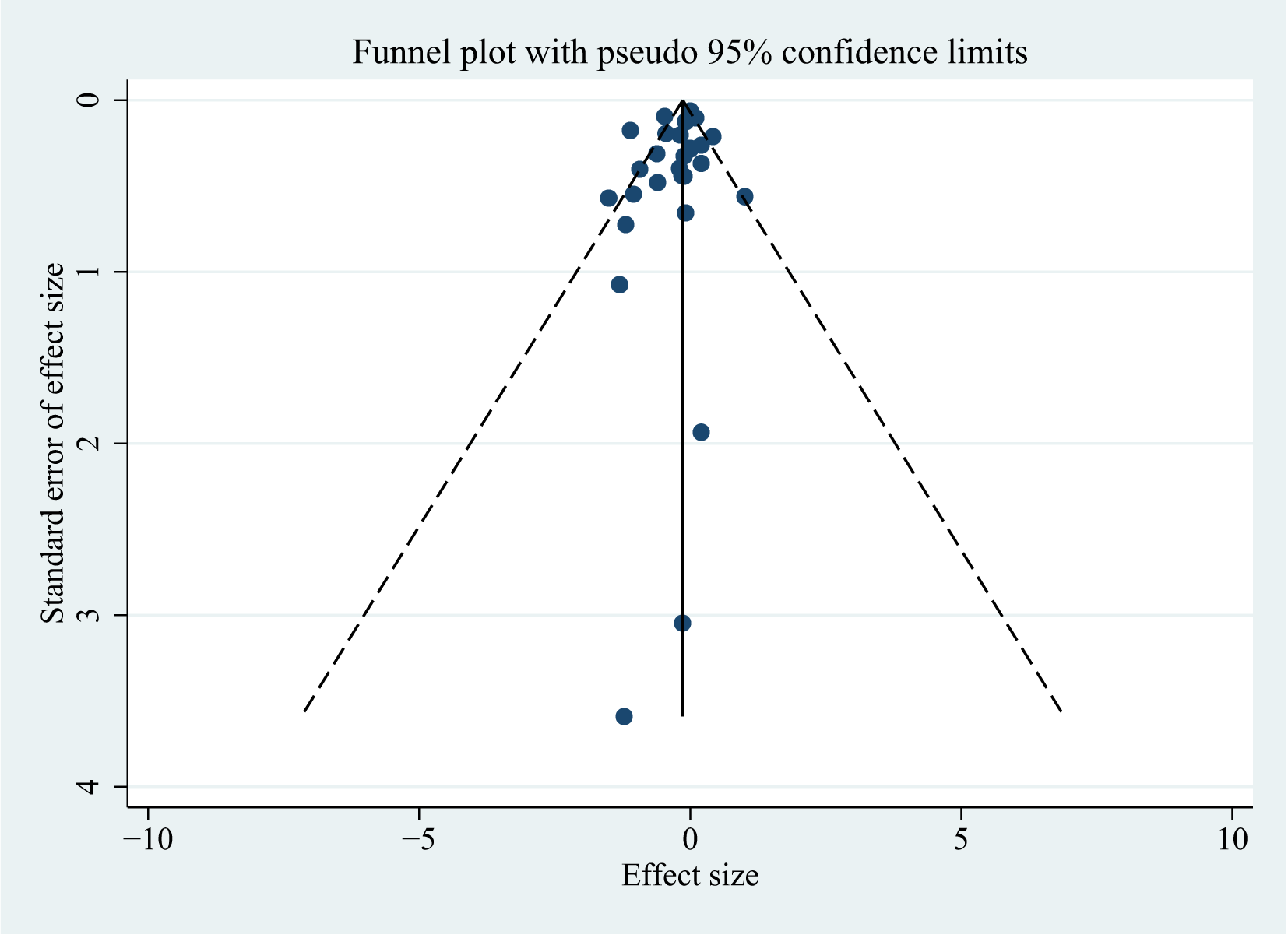

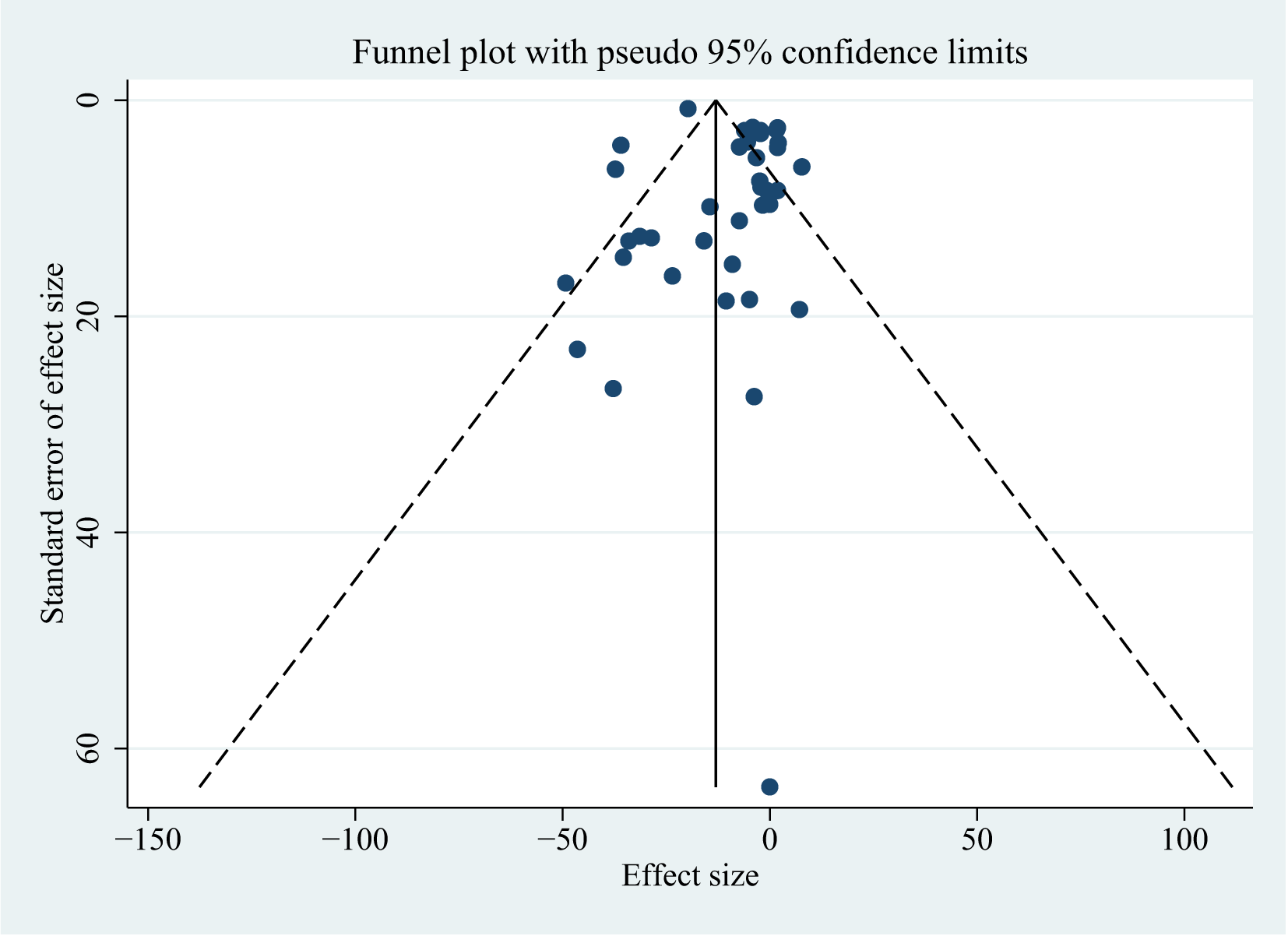


**Fig.S3.G FINS**  **Fig.S3.H HOMA-IR**


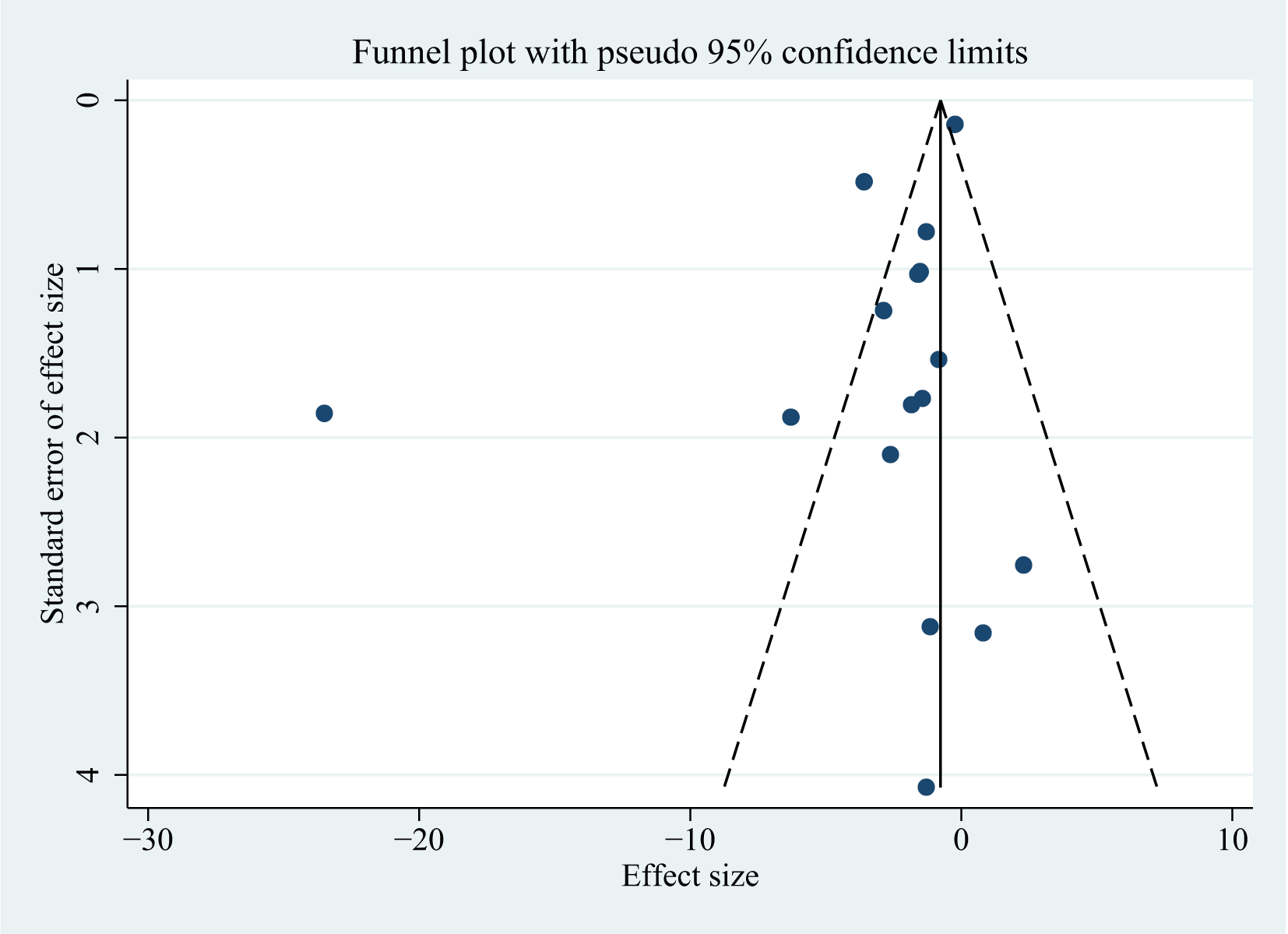

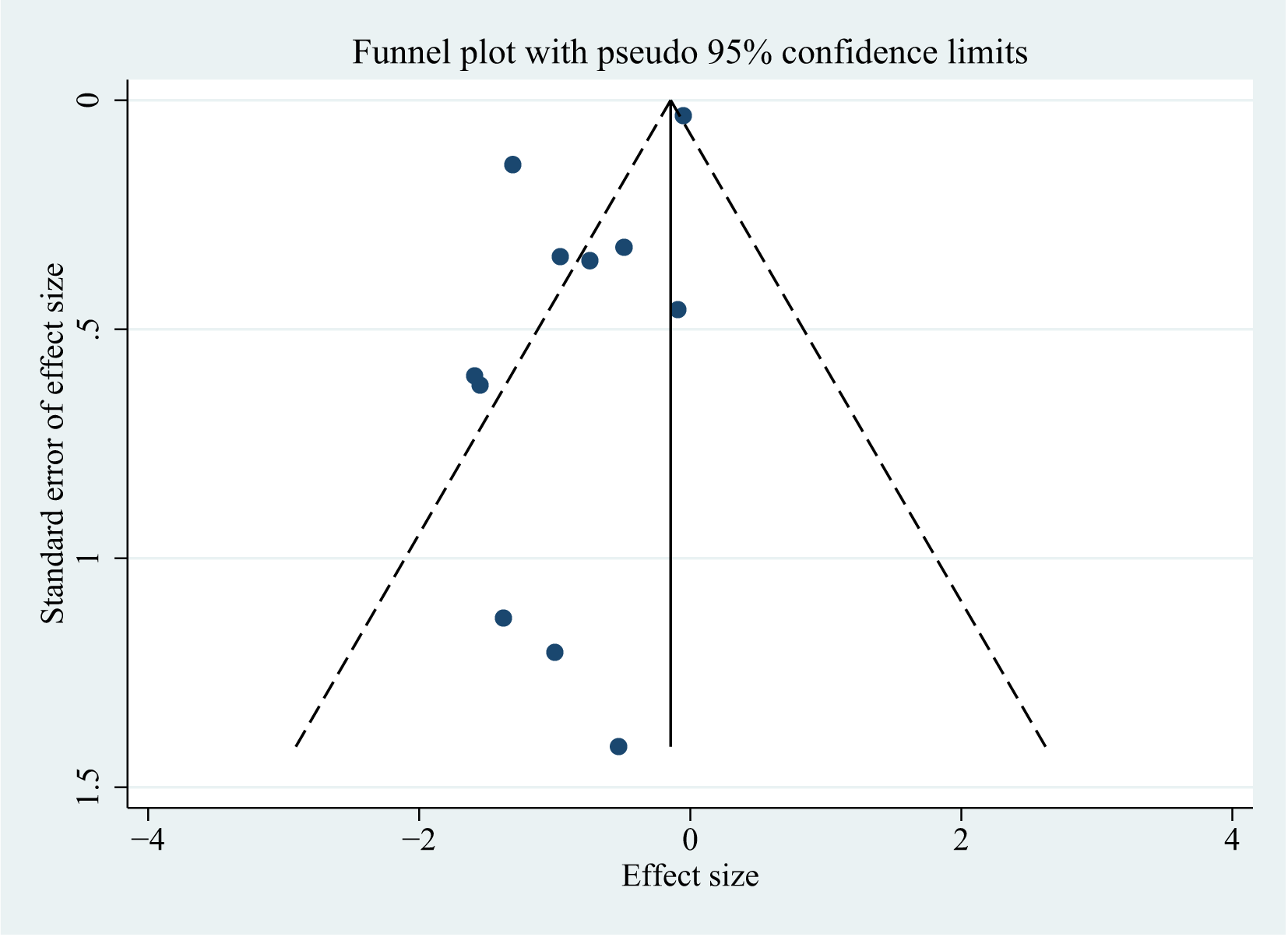


**Fig.S3.I CRP**  **Fig.S3.J IL-6**


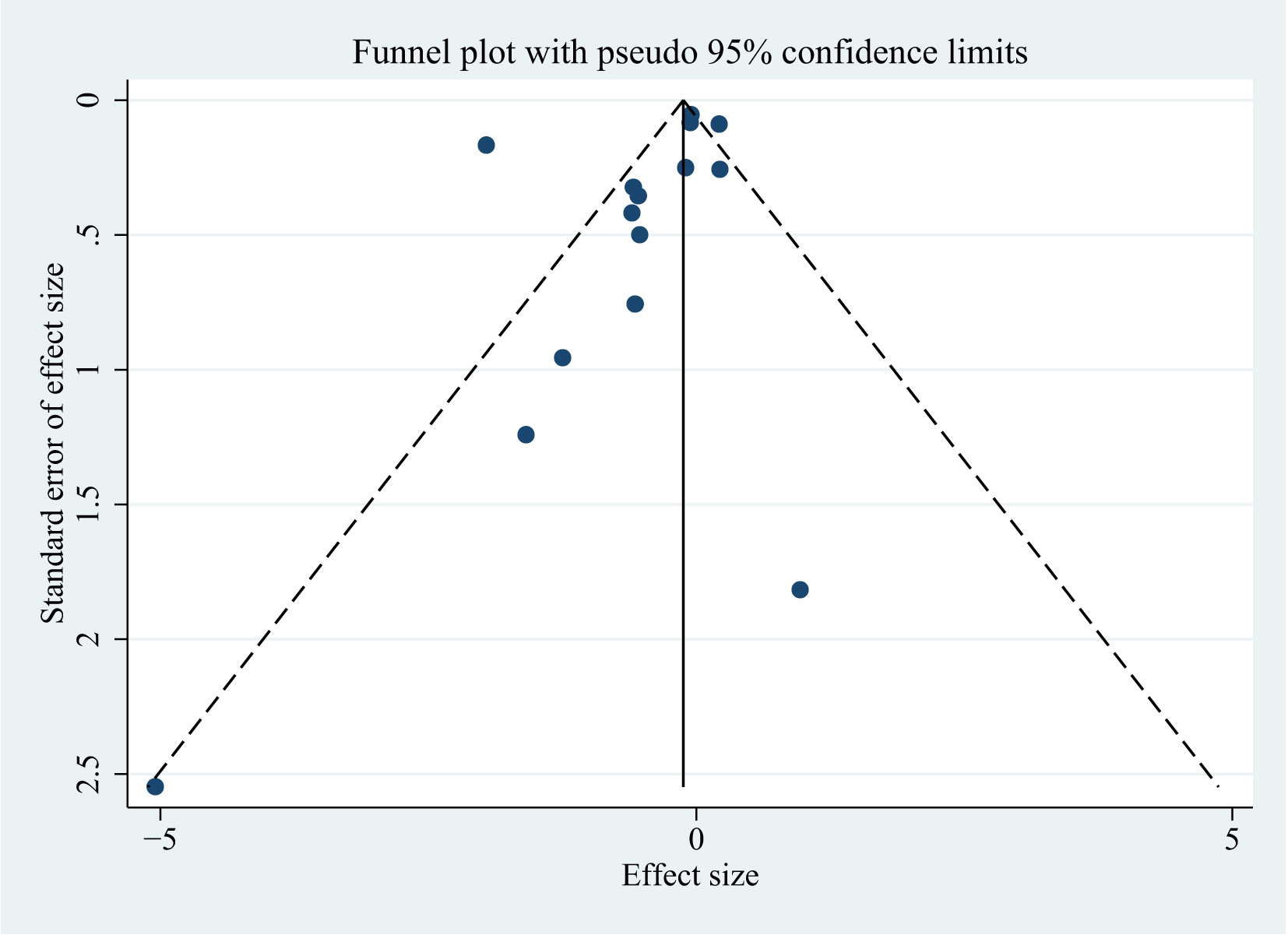

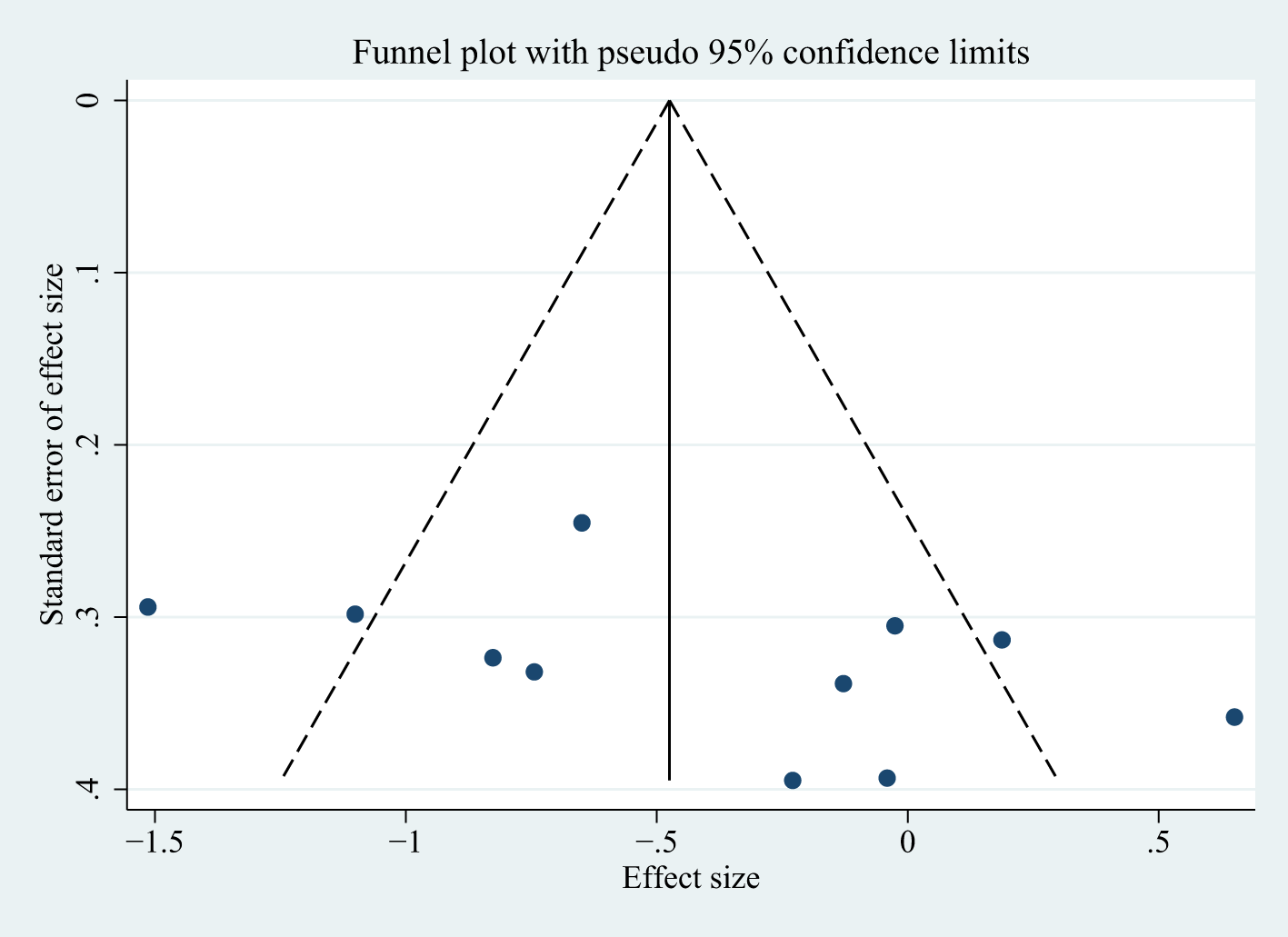

Supplement: Supplementary file 6 — Supporting Information 6 Figure S3: Funnel plots of (A) TG, (B) TC, (C) HDL‐C, (D) LDL‐C, (E) HbA1c, (F) fasting glucose, (G) FINS, (H) HOMA‐IR, (I) CPR, and (J) IL‐6. [file JDR-2026-5587445-s005.docx]
