# Supplementary material for: Effects of Coenzyme Q10 on Lipid, Glycemic, and Inflammatory Markers in Metabolic Disorders: A Systematic Review and Meta‐Analysis
Source: J Diabetes Res. 2026 May 26;2026:5587445. doi: 10.1155/jdr/5587445 (PMC13212042; doi:10.1155/jdr/5587445)
Supplement: Supplementary file 7 — Supporting Information 7 Figure S4: The trim‐and‐fill method for (A) fasting glucose and (B) HOMA‐IR. [file JDR-2026-5587445-s006.docx]

**Supplementary file 7: The trim and fill method for Fasting glucose, FINS and HOMA-IR**

**Fig.S4.A Fasting glucose Fig.S4.B HOMA-IR**

**
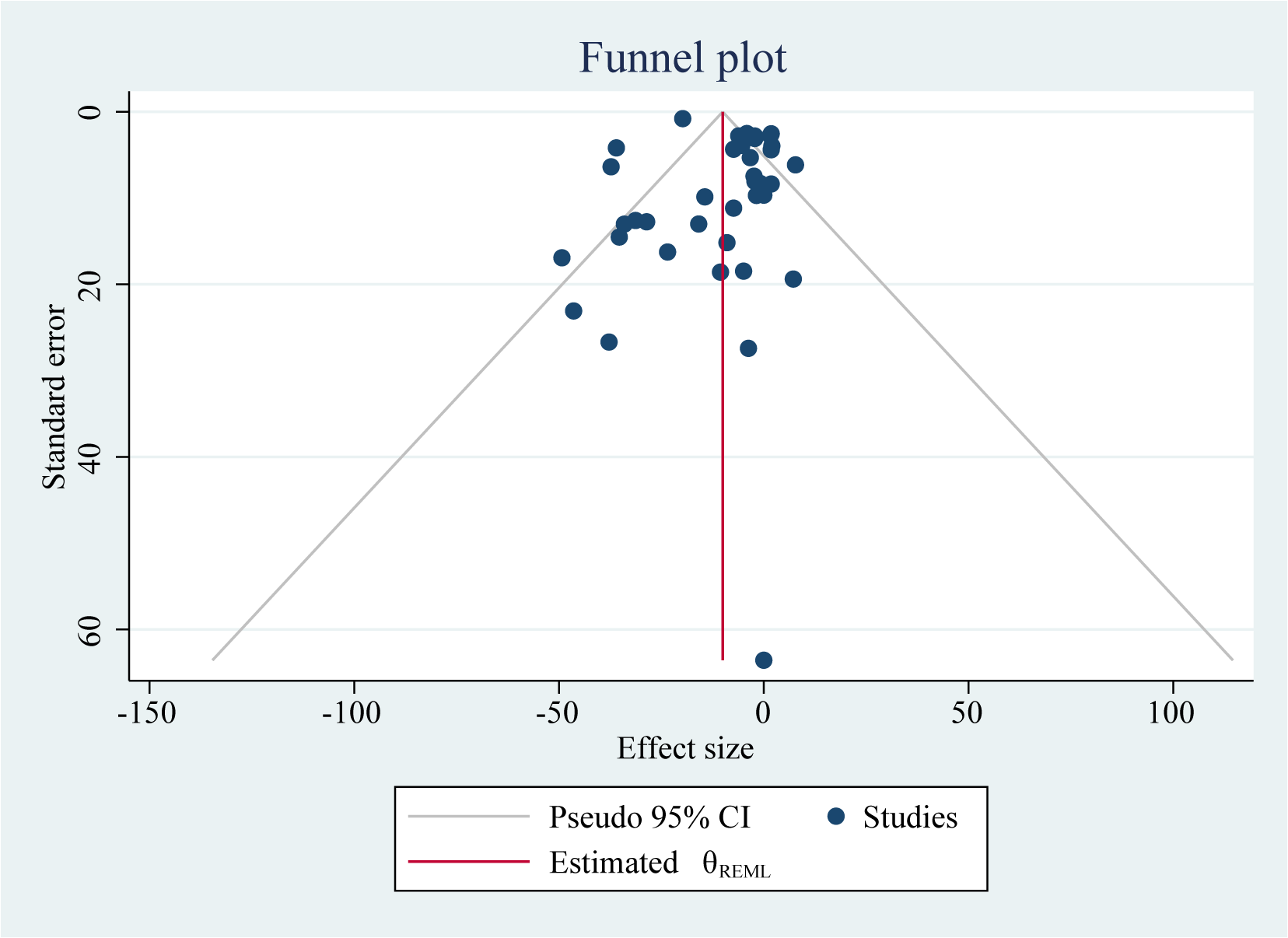

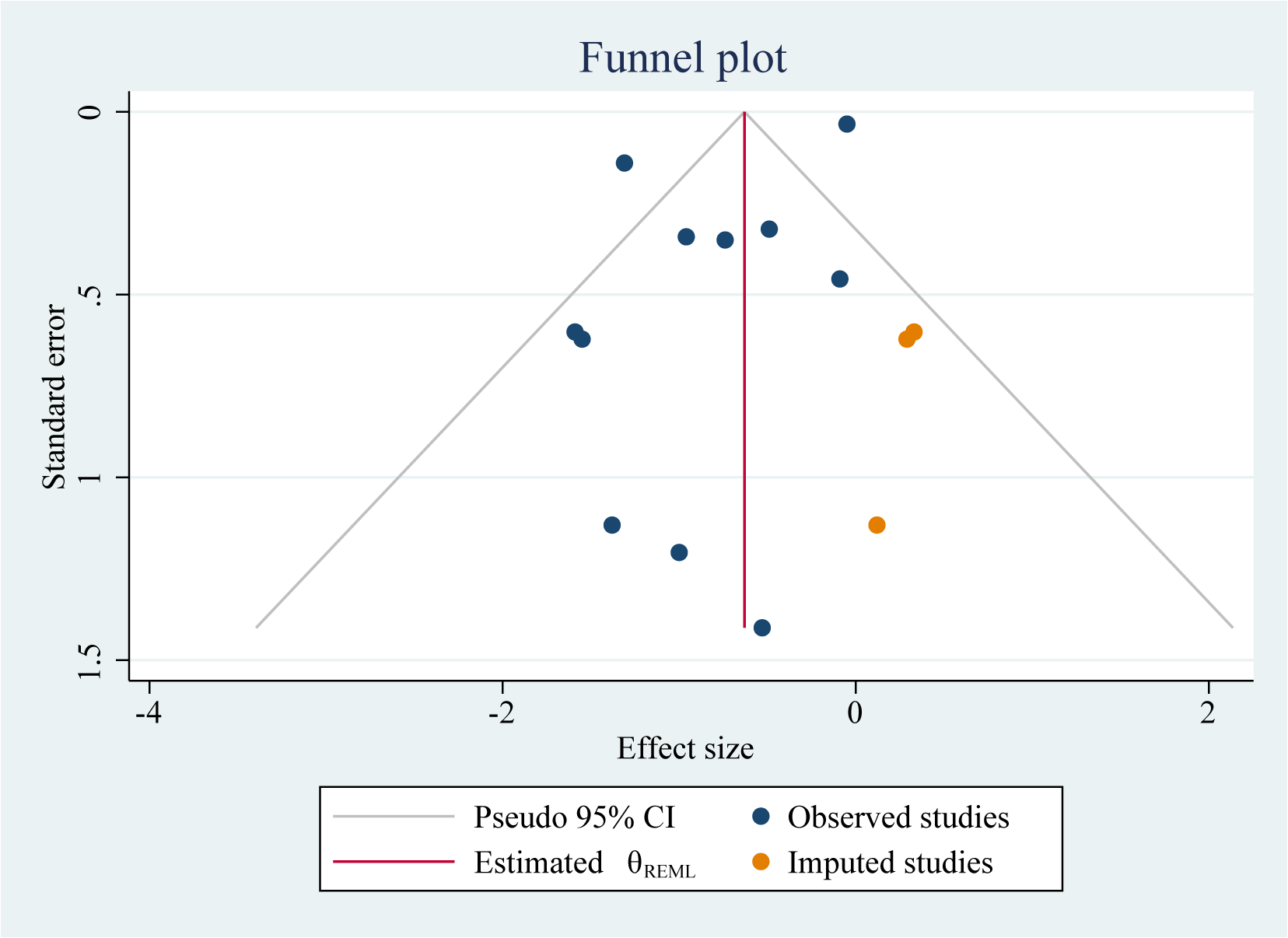
**
