# Supplementary figures and images for: Effects of Coenzyme Q10 on Lipid, Glycemic, and Inflammatory Markers in Metabolic Disorders: A Systematic Review and Meta‐Analysis
Source: J Diabetes Res. 2026 May 26;2026:5587445. doi: 10.1155/jdr/5587445 (PMC13212042; doi:10.1155/jdr/5587445)

# **Supplementary file 8: Individual study risk of bias assessments**


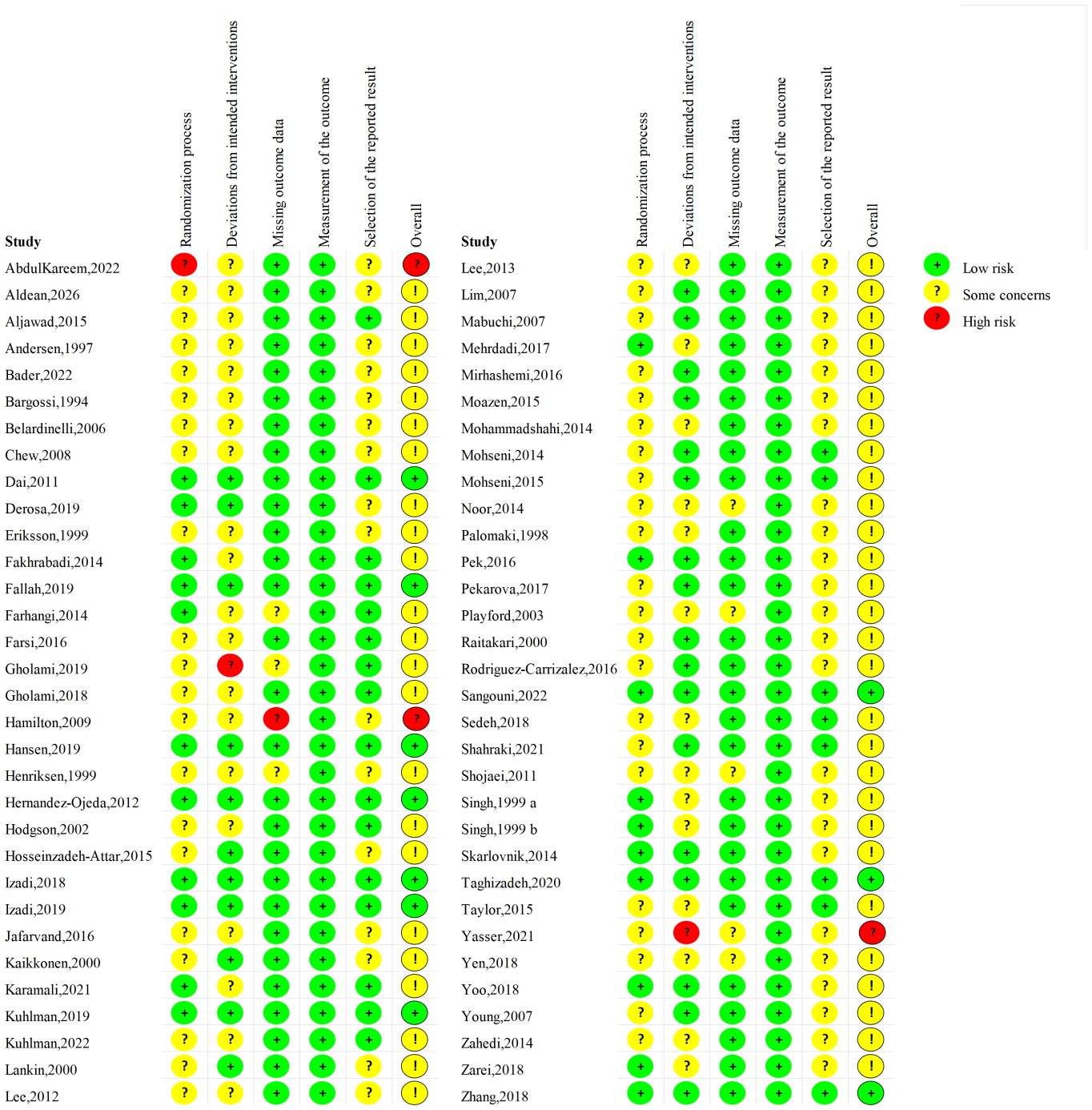

Supplement: Supplementary file 8 — Supporting Information 8 Supporting File S8: Individual study risk of bias assessments. [file JDR-2026-5587445-s003.docx]
